# Gene regulation could be attributed to *TCF3* and other key transcription factors in the muscle of pubertal heifers

**DOI:** 10.1002/vms3.278

**Published:** 2020-05-20

**Authors:** Li Yieng Lau, Loan T. Nguyen, Antonio Reverter, Stephen S. Moore, Aaron Lynn, Liam McBride‐Kelly, Louis Phillips‐Rose, Mackenzie Plath, Rhys Macfarlane, Vanisha Vasudivan, Lachlan Morton, Ryan Ardley, Yunan Ye, Marina R. S. Fortes

**Affiliations:** ^1^ School of Chemistry and Molecular Biology The University of Queensland Brisbane QLD Australia; ^2^ Queensland Alliance for Agriculture and Food Innovation The University of Queensland Brisbane QLD Australia; ^3^ CSIRO Agriculture and Food Queensland Biosciences Precinct Brisbane QLD Australia

**Keywords:** *Bos indicus*, gene network, muscle, puberty, RNA‐sequencing, *TCF3*

## Abstract

Puberty is a whole‐body event, driven by the hypothalamic integration of peripheral signals such as leptin or IGF‐1. In the process of puberty, reproductive development is simultaneous to growth, including muscle growth. To enhance our understanding of muscle function related to puberty, we performed transcriptome analyses of muscle samples from six pre‐ and six post‐pubertal Brahman heifers (*Bos indicus*). Our aims were to perform differential expression analyses and co‐expression analyses to derive a regulatory gene network associate with puberty. As a result, we identified 431 differentially expressed (DEx) transcripts (genes and non‐coding RNAs) when comparing pre‐ to post‐pubertal average gene expression. The DEx transcripts were compared with all expressed transcripts in our samples (over 14,000 transcripts) for functional enrichment analyses. The DEx transcripts were associated with “extracellular region,” “inflammatory response” and “hormone activity” (adjusted *p* < .05). Inflammatory response for muscle regeneration is a necessary aspect of muscle growth, which is accelerated during puberty. The term “hormone activity” may signal genes that respond to progesterone signalling in the muscle, as the presence of this hormone is an important difference between pre‐ and post‐pubertal heifers in our experimental design. The DEx transcript with the highest average expression difference was a mitochondrial gene, *ENSBTAG00000043574* that might be another important link between energy metabolism and puberty. In the derived co‐expression gene network, we identified six hub genes: *CDC5L, MYC, TCF3, RUNX2, ATF2* and *CREB1.* In the same network, 48 key regulators of DEx transcripts were identified, using a regulatory impact factor metric. The hub gene *TCF3* was also a key regulator. The majority of the key regulators (22 genes) are members of the zinc finger family, which has been implicated in bovine puberty in other tissues. In conclusion, we described how puberty may affect muscle gene expression in cattle.

## Graphical abstract

Puberty is a whole‐body event. In the process of puberty, reproductive development is simultaneous to muscle growth. To enhance our understanding of muscle function related to puberty, we performed whole‐genome gene expression analyses. As a result, we identified 431 differentially expressed genes and predicted a gene network. In the derived co‐expression gene network, *TCF3* was also a key regulator. In short, we described how puberty may affect muscle gene expression in cattle.

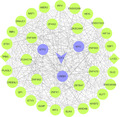

## 1 INTRODUCTION

Meat is an important end product of livestock production (Purslow, 2017). Skeletal muscle is the tissue sold as meat and its development from embryogenesis to maturation is a multistage process coordinating myogenesis, adipogenesis and fibrogenesis, and the subsequent growth of muscle, fat and connective tissues (Purslow, 2017). Growth starts from zygote formation and continues to the adult stage where mature weight or reproductive ability is achieved through puberty (Arango & Vleck, 2002). During puberty, muscle mass grows with the body and contributes to the pubertal growth spurt that results in adult height and body weight (Soliman, De Sanctis, Elalaily, & Bedair, 2014; Xu, Nicholson, Wang, Alén, & Cheng, 2009). Despite this fundamental change for muscle during puberty, this tissue has received relatively little consideration in the context of pubertal development.

To increase our understanding of the role of muscle tissue in puberty, changes to the muscle transcriptome were analysed by comparing pre‐ to post‐pubertal *Bos indicus* Brahman heifers. Brahman is a predominant breed in Northern Australia due to its ability in surviving the extreme heat, low‐quality dry pastures and being tick resistant (Abeygunawardena & Dematawewa, 2004; Prayaga et al., 2009). However, *Bos indicus* cattle reproduces at lower rates when compared with *Bos taurus* breeds, particularly when they have a suckling calf (Abeygunawardena & Dematawewa, 2004; Brito et al., 2004; Nogueira, 2004). The age of puberty of *Bos indicus* ranges from 10 to 40 months of age with an average value of 25 months in Brahman herds. In contrast, *Bos taurus* are pubertal at about 7–13 months (Gregory, Laster, Cundiff, Smith, & Koch, 1979; Morgan, 1981). Puberty, defined as age when the first *corpus luteum* was observed, is a highly heritable trait (h2 = 0.42). It is therefore possible to reduce the onset of puberty and improve the genetic gain (Johnston et al., 2014; MacNeil, Cundiff, Dinkel, & Koch, 1984; Vargas, Olson, Chase, Hammond, & Elzo, 1999).

In this study, we used transcriptomics (mRNA‐sequencing) to measure global gene expression of six pre‐ and six post‐pubertal Brahman heifers. Knowledge of specific genes, mutations and gene networks obtained could be beneficial to enhance genomic selection (Fortes, Porto‐Neto, et al., 2014; Perez‐Enciso, Rincon, & Legarra, 2015; Snelling et al., 2013). We performed functional enrichment analysis, derived and analysed a co‐expression network to improve our understanding of the differentially expressed genes that were associated with puberty.

## 2 MATERIALS AND METHODS

Animal handling, euthanasia and sampling were approved by the Animal Ethics Committee of The University of Queensland, Production and Companion Animal group (certificate number QAAFI/279/12). These animals are the same heifers used in previous studies that described gene expression results for other tissues, such as hypothalamus, pituitary gland, ovaries, liver and adipose tissue (Fortes, Nguyen, Weller, et al., 2016; Nguyen, Reverter, et al., 2018; Nguyen et al., 2017; Nguyen, Zacchi, Schulz, Moore, & Fortes, 2018). Here, we used data from all studied tissues to inform the linear models used to normalize gene expression counts, considering the effect of tissue. This normalization method was previously established as a rigorous approach for analysing microarray and transcriptome data (Canovas et al., 2014; Harper et al., 2004). After normalization, we focused only on muscle data. We analyse the muscle results and derive a gene co‐expression network, described for peri‐pubertal *Bos indicus* heifers for the first time.

### 2.1 Animals and muscle samples

Six pre‐ and six post‐pubertal heifers were euthanized for collection of muscle samples. Puberty was defined by the first observed *corpus luteum* (CL) with the use of ultrasound. In detail, post‐puberty heifers were in the luteal phase of their second cycle (Fortes, Nguyen, Weller, et al., 2016). Samples from *longissimus dorsi* muscle (LDM) were collected with a biopsy punch that cut approximately 1cm^3^ from the tissue of pre‐ and post‐pubertal heifers. LDM samples were snapped frozen in liquid nitrogen and stored in a freezer (−80°C) until RNA extraction. In total, 12 LDM samples were processed separately for RNA extraction and sequencing.

### 2.2 RNA extraction, purification and sequencing

Total RNA was isolated from fragmented and homogenized frozen LDM tissue, using a RNeasy mini kit (QIAGEN Pty Ltd., Melbourne, VIC, Australia) combined with Trizol methods (Life Technologies Inc.) as previously described by Fortes et al. (2016). Quality of the total RNA was evaluated using the RNA integrity number (RIN) measured with an Agilent Bioanalyzer 2,100 (Agilent Technologies) and all samples had RIN above 6.9. From total RNA, we used the TruSeq RNA sample preparation kit (Illumina Inc.), which includes a mRNA purification step for cDNA library preparation (performed according to the kit standard protocol). Six paired‐end libraries were sequenced per lane in an Illumina HiSeq 2000 analyser (Illumina Inc.). After sequencing, we used the CLC Genomics workbench software (CLC bio) to map the sequence reads to the reference bovine genome (UMD3.1, release annotation 77; ftp://ftp.ensembl.org/pub/release‐77/genbank/bos_taurus) and count the number of reads per gene, using the procedures and quality control steps described before (Canovas et al., 2014). Quality control analysis was performed with the CLC Genomics workbench, which takes into consideration read length, coverage and ambiguities, implementing FastQC tools (http://www.bioinformatics.babraham.ac.uk/projects/fastqc) as described by Cánovas et al. (2014). We then analysed the RPKM (reads per kilo base per million mapped reads) values for all genes that were expressed above a noise threshold of 0.2 RPKM, which is commonly used in bovine transcriptomics (Wickramasinghe et al., 2011). Only genes with average RPKM ≥ 0.2 in at least one tissue were considered expressed and had their data used in subsequent analyses. Using RPKM values for expressed genes, we performed a Principal Component Analysis (PCA) that we run for quality assurance, as means to check the overall profile of gene expression across all studied tissues and samples. One of the sequenced muscle pre‐pubertal samples clustered apart from the rest of the muscle samples in our PCA. We cannot be certain if poor sequencing quality or sampling error led to this mismatch between all eleven muscle samples that grouped together and one that was not in the same PCA cluster. Hence, the particular *faulty* sample was disregarded for further analysis, which continued with 11 samples: five pre‐puberty and six post‐puberty.

### 2.3 Identification of differentially expressed genes

As genes with low counts can be easily biased without transformation, the base‐2 log‐transformed RPKM values were used in this analysis of differential expression. Mixed‐model equations that consider the interaction between individual and tissue variation to further adjust gene expression data were used (Canovas et al., 2014; Harper et al., 2004; Reverter et al., 2006). In brief, the library was fitted as a fixed effect, the main effect of gene was fitted as a random effect and the effect of the tissue/gene/animal interaction was fitted as a random interaction. This study is part of a larger study where six tissues were sampled per animal. The VCE6 software (ftp://ftp.tzv.fal.de/pub.vce6) was used to estimate variance components and to obtain mixed‐model solutions. The model used compared gene expression of pre‐ versus post‐pubertal animals to test for each gene the hypothesis that expression was differential and the threshold *p*‐value of <.05 was used, in consideration of the strict normalization performed and subsequent analyses, which used the DEx genes for further perusal. This approach considers DEx genes as a step towards identifying regulators of gene expression in a systems biology approach, as established beforehand (Fortes, Suhaimi, et al., 2014; Nguyen, Reverter, et al., 2018; Nguyen et al., 2017).

### 2.4 Gene ontology and pathway enrichment analyses

To identify enriched gene ontology (GO) terms and pathways within the identified DEx genes, we used the Database for Annotation, Visualization and Integrated Discovery (DAVID), with the list of DEx genes used as the query list and all the genes expressed in muscle as the background list (Dennis et al., 2003; da Huang, Sherman, & Lempicki, 2009). DAVID functional annotation tools revealed overrepresented GO terms and pathways associated with the DEx genes, significant result after correction for multiple testing with Benjamini–Hochberg method are presented and discussed in this paper (adjusted *p*‐value <.05).

### 2.5 Gene network prediction

To further our understanding of the gene expression patterns in our samples, we constructed a co‐expression gene network by employing a partial correlation and information theory (PCIT) algorithm that was developed for this purpose (Reverter & Chan, 2008). In short, partial correlation is the correlation between two genes that is independent of a third gene and in PCIT. This is a data‐driven approach: all the correlations between possible triplets of genes were explored prior to the identification of significant correlations that are within the extremes of the distribution (Reverter & Chan, 2008). Our objective for the co‐expression analyses was to identify the interactions between all the 431 DEx genes and its possible regulators. We used expression values of all DEx genes and all expressed (i.e. DEx or not) transcription factors (TF) in our muscle samples. To identify which genes were classified as TF, we used the AnimalTFBS3.0 database as reference (http://bioinfo.life.hust.edu.cn/AnimalTFDB/#!/tf_summary?species=Bos_taurus; accessed September 18, 2018) (Jia et al., 2018). The inclusion of TF in the network could provide extra information to infer gene function, and gene regulation that might be particularly useful when a gene is unannotated. After identifying TF that were expressed but were not DEx, we merged this new list with all DEx to create an input list of 1,441 genes for PCIT analysis. The resulting gene co‐expression network was visualized and analysed with Cytoscape software (Shannon et al., 2003).

### 2.6 Identification of hub genes

In network theory, a gene is defined as a *hub* if it is highly connected: it is connected to a number of genes that is greater than the average connection across all genes in the network (Barabasi & Oltvai, 2004; Bi, Ning, Liu, Que, & Ding, 2015; Stumpf & Porter, 2012). Two networks were subjected to Network Analysis in Cytoscape; gene co‐expression network computed by PCIT and protein–protein interaction (PPI) network generated by Search Tool for the Retrieval of Interacting Genes/Proteins (STRING) (https://string‐db.org/cgi/input.pl) (Szklarczyk et al., 2018). We focused on the degree parameter as it represents the number of edges (connections) a node (gene) has, supporting the definition of a hub gene. Genes with a degree of more than two standard deviations (*SD*) in both the gene co‐expression and PPI networks were identified as hub genes.

### 2.7 Identification of key transcription factor (TF) as regulatory elements

Using a regulatory impact factor (RIF) metric, key regulators were identified among all the TF expressed in muscle tissue (Hudson, Reverter, Wang, Greenwood, & Dalrymple, 2009; Antonio Reverter, Hudson, Nagaraj, Pérez‐Enciso, & Dalrymple, 2010). The RIF metric was explored in two measures: RIF1 and RIF2 that were computed as per the original publications that applied RIF to gene expression datasets (Hudson et al., 2009; Antonio Reverter et al., 2010). The RIF metrics are based on the interactions between TF and target genes; the DEx genes were the target genes in this study (Reverter et al., 2010). RIF1 represents TF that are constantly most differentially co‐expressed with the highly abundant and highly DEx genes, whereas RIF2 represents TF with the highest ability as predictors of change in the abundance of DEx genes (Reverter et al., 2010). For the purpose of comparison between RIF1 and RIF2 as well as comparison across datasets, RIF measures were further transformed to a z‐score by subtracting the mean and dividing it by the standard deviation (*SD*). Using *p* < .05 as a cut‐off, a TF was considered a key regulator if any of the RIF scores was higher than 1.96 *SD* units (Nguyen, Reverter, et al., 2018).

### 2.8 Promoter region analysis

Promoter regions corresponding to the DEx genes were retrieved and used to predict associated transcription factors from annotated affinities using the web‐based tool known as PASTAA (http://trap.molgen.mpg.de/cgi‐bin/pastaa.cgi) (Roider, Manke, O'Keeffe, Vingron, & Haas, 2009). Significant TFs matrix (*p*‐value < .05) associated with the DEx genes were selected for further analyses.

## 3 RESULTS

**FIGURE 1:**
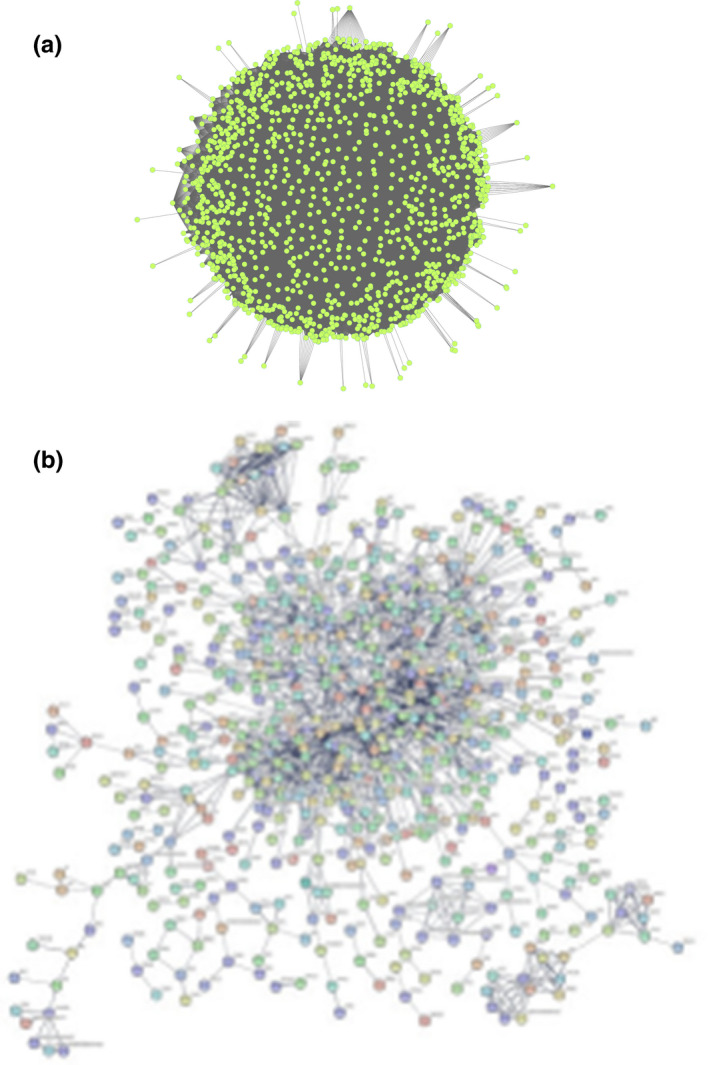
(a) Gene co‐expression network computed by Partial Correlation and Information Theory (PCIT) algorithm and illustrated with Cytoscape. (b) Protein–protein interaction (PPI) network analysed by STRING database (https://string‐db.org/cgi/input.pl)

A total of 14,437 transcripts were expressed in the muscle samples of Brahman heifers (RPKM value greater than 0.2 on average). Among these transcripts, 431 were differentially expressed (DEx, *p*‐value < .01) in the pre‐ versus post‐pubertal comparison. Of the 431 DEx transcripts (genes and non‐coding RNAs), 238 transcripts were upregulated and 193 transcripts were downregulated after puberty (Figure 1, Table S1). Among all the DEx transcripts, 175 transcripts increased or decreased at least twofold after puberty (Table S1). Table 1 summarizes the transcripts with high average expression difference (POST – PRE > 4) in the muscle tissue of Brahman heifers. Note that the top two transcripts with highest average expression difference were *ENSBTAG00000043574* and *ENSBTAG00000025485* and both of them are novel genes in terms of the bovine genome functional annotation. According to Ensembl, the gene *ENSBTAG00000043574* is a mitochondrial transfer ribonucleic acid (Mt‐tRNA) and *ENSBTAG00000025485* is a member of the glutathione S‐transferase (GSTA) family. Some DEx transcripts were previously identified to be involved in puberty (Table S1). For example *IGFBP2* that belongs to the IGF1 pathway, *OXT* that codes for oxytocin and *PRL* that codes for the prolactin hormone, all have known roles in reproductive biology. In short, the functions of some of the DEx transcripts are easier than others to interpret in the context of puberty. The functions of 8 top DEx transcript (POST ‐ PRE > 4|) will be further explored in the discussion of this manuscript.

**TABLE 1:** Top differentially expressed (DEx) transcripts in the muscle tissue of pre‐ and post‐pubertal Brahman heifers (|FC > 4|)

ENSB tag^a^	Gene^b^	POST^c^	PRE^c^	Average expression difference	FC^d^
ENSBTAG00000043574	*ENS43574*	7.19	1.91	5.28	3.76
ENSBTAG00000025485	*ENS25485*	1.95	7.12	−5.17	0.27
ENSBTAG00000042678	*SNORA71*	2.01	6.94	−4.93	0.29
ENSBTAG00000045039	*SCARNA17*	7.40	2.55	4.85	2.90
ENSBTAG00000044778	*U6*	2.14	6.91	−4.77	0.31
ENSBTAG00000043695	*bta‐mir−450b*	8.59	3.88	4.70	2.21
ENSBTAG00000031600	*ENS31600*	2.51	7.06	−4.55	0.36
ENSBTAG00000048516	*ENS48156*	8.61	4.26	4.35	2.02

Abbreviations: FC, Fold change.

^a^ ENSB tag: Ensembl gene identifier according to http://www.ensembl.org/.

^b^ Gene symbol.

^c^ POST is the average gene expression after puberty and PRE is the average gene expression before puberty.

^d^ FC (fold change) describes the ratio of expression between pre‐ and post‐puberty; FC = POST/PRE. FC between 0 and 0.5 means post‐pubertal expression has decreased more than 2 folds in comparison to pre‐pubertal expression, FC between 0.5 to 1 means no difference in expression in both pubertal stages, whereas FC of more than 1 indicates increase in expression at post‐pubertal.

Functional enrichment analyses identified “extracellular region,” “extracellular space” and “extracellular region part” as the most overrepresented GO terms (significantly enriched) in the cellular component category, indicating that most DEx transcripts are located in extracellular region and may be associated with the extracellular matrix (Table 2, *p* < .05). In the biological processes category, the GO terms “inflammatory response” and “defence response” were significantly enriched, indicating that DEx transcripts could be involved in these processes (Table 2). As for the molecular function category, the GO term “hormone activity” was the only term that was significant (Table 2, adjusted *p*‐value < .05). We were able to group some DEx transcripts into three KEGG pathways, although no pathway was significantly enriched (Table 3, adjusted *p*‐value < .05). Nevertheless, two of the pathways identified, namely the retinol metabolism and steroid hormone biosynthesis, are known to be related to puberty. The DEx transcripts *AKR1D1*, *CYP1B1*, *ENS40337*, *ENS37890*, *RDH16* and *CYP1A2* were all downregulated after puberty.

**TABLE 2:** GO (gene ontology) terms that were significantly enriched in the muscle samples

Category	GO Term	Description^a^	Count^b^	%^c^	Adjusted *p*‐value
GOTERM_CC_FAT	GO:0005576	Extracellular region	39	11.1	3 × 10^−9^
GOTERM_CC_FAT	GO:0005615	Extracellular space	16	4.6	1 × 10^−3^
GOTERM_CC_FAT	GO:0044421	Extracellular region part	17	4.9	4 × 10^−2^
GOTERM_BP_FAT	GO:0006952	Defence response	13	3.7	4 × 10^−2^
GOTERM_BP_FAT	GO:0006954	Inflammatory response	10	2.9	2 × 10^−2^
GOTERM_MF_FAT	GO:0005179	Hormone activity	6	1.7	2 × 10^−2^

Note

Results obtained using DAVID enrichment analyses (https://david‐d.ncifcrf.gov/). Only GO terms with *p* < .05 after correction of multiple testing were considered significant and included in this table. CC: Cellular component, MF: Molecular function, BP: Biological process.

^a^ Description: the function related to the group of genes.

^b^ Count: number of genes involved in the respective category.

^c^ %: fraction of genes involved in the category as compared with all the genes listed.

**TABLE 3:** Three KEGG (Kyoto Encyclopedia of Genes and Genomes) pathways enriched in the muscle samples

Entry^a^	Pathway	Count^b^	%^c^	Adjusted *p*‐value
bta04610	Complement and coagulation cascades	7	2	9.20 × 10^−2^
bta00830	Retinol metabolism	4	1.1	6.50 × 10^−1^
bta00140	Steroid hormone biosynthesis	3	0.9	8.80 × 10^−1^

Note

Results obtained using DAVID enrichment analyses (https://david‐d.ncifcrf.gov/). Pathway with corrected *p*‐ value < .05 is considered significantly enriched.

^a^ Entry: unique KEGG pathway identifier representing specific pathway.

^b^ Count: number of genes involved in the pathway.

^c^ %: fraction of genes that are involved in the pathway.

The PCIT derived co‐expression gene network comprised of 1,441 transcripts with 55,091 connections (Figure 1), whereas the PPI network contained 646 interconnected proteins forming 1,706 connections. Cytoscape network analyses of both the gene co‐expression and the PPI networks revealed six hub genes, as these genes fulfilled our selection criteria of more than 2 *SD* in both networks. Our hub genes were *MYC*, *CREB1*, *TCF3*, *ATF2, CDC5L* and *RUNX2* and their roles will be individually explored in the discussion. Another pathway enrichment analysis was performed with the 1,441 connected transcripts in the network and resulted in significant enrichment of “extracellular region” and “extracellular space,” further confirming the importance of these GO terms in muscle samples of Brahman heifers, in the context of puberty.

To identify key regulatory genes in the muscle sample, RIF metric was applied to all 1,452 TFs expressed in the muscle samples, DEx or not (Table S2). The RIF analyses identified 48 key TFs (*SD* > 1.96 units) where nearly half of them (22 key TFs) belong to the zinc finger family (Table 4). Other major family of TFs include the bHLH (basic helix‐loop‐helix) family (*TAL1, HES4, SREBF2, MXD4* and *TCF3*) and the homeobox family (*DLX5, EMX2, IRX2* and *HOXA6*) as listed in Table 4. Of note, other than being a key TF, *HES4* is also a DEx gene and *TCF3* is also a hub gene. The key TFs with high RIFs (RIF1 or RIF2 > 3 *SD* units) were *ZNF419*, *ENSBTAG00000038926*, *DLX5* and *EMX2.* Interestingly, *ZNF419* and *ENSBTAG00000038926* are members of the zinc finger family, whereas *DLX5* and *EMX2* are TFs from the homeobox family. All the top key TFs had a lower pre‐pubertal expression, with one exception: *EMX2* demonstrated a higher pre‐pubertal expression level. Other noteworthy key TFs, were *ZIC4* and members of the *GATA* family, *GATA2* and *GATA6,* due to their relevant role in previous studies (see the Discussion). An enrichment analysis revealed no significant pathway related to the key TFs.

**TABLE 4:** Transcription factors that are important regulators according to the regulatory impact factor (RIF) metric in muscle tissues (RIF > 1.96)

ENSB tag^a^	Gene^b^	Family^c^	RIF1^d^	RIF2^d^
ENSBTAG00000017613	*ZNF419*	zf‐C2H2	4.10	1.50
ENSBTAG00000018645	*DLX5*	Homeobox	4.05	1.00
ENSBTAG00000003027	*EMX2*	Homeobox	3.42	1.40
ENSBTAG00000038926	*ENSBTAG00000038926*	zf‐C2H2	3.35	1.38
ENSBTAG00000018131	*ATF1*	TF_bZIP	2.01	2.68
ENSBTAG00000002291	*ZBTB41*	ZBTB	2.51	1.59
ENSBTAG00000002690	*BLZF1*	Others	2.29	1.76
ENSBTAG00000017651	*ENSBTAG00000017651*	zf‐C2H2	2.33	1.70
ENSBTAG00000024115	*FOXO6*	Fork_head	2.49	1.38
ENSBTAG00000005572	*ZNF205*	zf‐C2H2	2.19	−1.68
ENSBTAG00000030939	*ZNF575*	zf‐C2H2	2.54	1.33
ENSBTAG00000014749	*ZIC4*	zf‐C2H2	2.24	1.61
ENSBTAG00000037440	*ENSBTAG00000037440*	zf‐C2H2	2.20	1.43
ENSBTAG00000026307	*ZNF629*	zf‐C2H2	2.49	1.09
ENSBTAG00000019885	*ZNF280C*	zf‐C2H2	2.62	0.89
ENSBTAG00000002201	*NFXL1*	zf‐NF‐X1	2.75	0.67
ENSBTAG00000012385	*NFX1*	zf‐NF‐X1	2.43	−0.99
ENSBTAG00000026408	*ZNF213*	zf‐C2H2	2.06	1.36
ENSBTAG00000012074	*MYB*	MYB	2.20	1.13
ENSBTAG00000040585	*NR6A1*	GCNF‐like	2.20	−1.10
ENSBTAG00000000054	*SNAPC4*	MYB	2.27	−0.98
ENSBTAG00000047405	*ENSBTAG00000047405*	zf‐C2H2	−2.08	−1.14
ENSBTAG00000023938	*CENPA*	Others	2.51	0.70
ENSBTAG00000047499	*IRX2*	Homeobox	2.07	1.14
ENSBTAG00000005734	*GATA6*	zf‐GATA	2.50	0.53
ENSBTAG00000005029	*TAL1*	bHLH	2.27	−0.75
ENSBTAG00000019707	*GATA2*	zf‐GATA	2.45	−0.56
ENSBTAG00000014705	*HES4*	bHLH	2.16	0.83
ENSBTAG00000002336	*RCOR1*	MYB	2.42	−0.56
ENSBTAG00000033563	*ZNF529*	zf‐C2H2	2.67	−0.30
ENSBTAG00000037906	*ENSBTAG00000037906*	zf‐C2H2	2.07	−0.88
ENSBTAG00000015879	*TULP3*	Tub	2.64	0.28
ENSBTAG00000008132	*SOX13*	HMG	2.81	0.09
ENSBTAG00000000195	*ENSBTAG00000000195*	zf‐C2H2	2.62	−0.26
ENSBTAG00000014265	*SREBF2*	bHLH	2.25	−0.63
ENSBTAG00000018270	*NFATC2*	RHD	2.32	0.55
ENSBTAG00000027442	*NFIB*	CTF/NFI	2.86	0.00
ENSBTAG00000011957	*CREBL2*	Others	2.45	0.36
ENSBTAG00000024341	*HOXA6*	Homeobox	2.14	−0.60
ENSBTAG00000000625	*SMAD6*	MH1	2.69	−0.03
ENSBTAG00000015209	*MXD4*	bHLH	2.33	0.28
ENSBTAG00000010568	*ZBED5*	zf‐BED	2.51	0.02
ENSBTAG00000003687	*FOXK2*	Fork_head	−2.00	0.43
ENSBTAG00000010130	*ZNF335*	zf‐C2H2	1.99	−0.42
ENSBTAG00000007983	*ZNF775*	zf‐C2H2	2.28	0.08
ENSBTAG00000038034	*ZNF628*	zf‐C2H2	2.05	0.30
ENSBTAG00000008695	*TCF3*	bHLH	−2.07	−0.26
ENSBTAG00000003457	*ATF5*	TF_bZIP	2.16	0.16

^a^ ENSB Tag: Ensembl gene identifier according to http://www.ensembl.org/.

^b^ Gene symbol.

^c^ Family: Transcription factor family.

^d^ RIF1 = regulatory impact factor 1; RIF2 = regulatory impact factor 2.

As a mean of determining the TFs binding sites (TFBS) in our samples, PASTAA analysis was performed with the list of DEx transcripts. A total of 57 matrixes (TFBS) were found to be significantly associated with our DEx transcripts (*p*‐value < .05) (Table S3). Specific TFBS were identified for *TCF3*. Therefore, *TCF3* was a hub gene, a key TF and a TF that could be identified in the promoter region analyses, that is three methods suggest its role as a regulator of gene expression in muscle, particularly important in the context of puberty.

## 4 DISCUSSION

The Hypothalamus‐Pituitary‐Gonadal (HPG) axis is central to puberty and reproduction, and yet it may not be the full story. There have been increasing evidence for the link between whole‐body function, metabolism and the establishment of an active reproductive system through puberty. For example, liver, a highly metabolic organ was found to be strongly correlated with reproduction where genes related to growth regulation and puberty onset were identified (Fontana & Della Torre, 2016; Montagner et al., 2016; Nguyen, Reverter, et al., 2018). Proteomic studies of adipose tissue identified proteins enriched in pathways that are known integrators of energy metabolism and reproduction (Nguyen, Reverter, et al., 2018). IGF1 and Leptin are accepted as peripheral signals that feedback to the HPG axis in a manner that is relevant to cattle puberty (Fortes, Li, Collis, Zhang, & Hawken, 2013; Narro, Thomas, Silver, Rozeboom, & Keisler, 2003; Nogueira & Paula Beltran, 2008). The role of peripheral tissues in the context of puberty is under ongoing investigation.

In our study, we found some consistent results with a puberty study in Brangus (*Bos indicus* crossed with *Bos taurus*) heifers (Canovas et al., 2014). For instance both studies found OXT gene to be downregulated between pre‐ and post‐puberty heifers, in the muscle. The GO term “hormone activity” was significantly enriched in DEx transcripts for both studies, which we will discussed in one of the following paragraphs (Canovas et al., 2014). Most importantly, our study adds evidence for the whole‐body theory by sampling the muscle of pre‐ and post‐pubertal heifers and identifying 431 DEx transcripts. Out of 431 DEx transcripts, 8 DEx transcripts demonstrated high average expression difference (POST – PRE > 4) between pre‐ and post‐puberty. They were *ENSBTAG00000043574*, *ENSBTAG00000025485, SNORA71, SCARNA17, U6, bta‐mir‐450b, ENSBTAG00000031600* and *ENSBTAG00000048156*. We elaborated on these transcripts in the following paragraphs.

Regulation of energy metabolism largely depends on mitochondria, an eukaryote organelle generating most energy in the cell through oxidative phosphorylation (OXPHOS) (Suzuki, Nagao, & Suzuki, 2011). In this study, the mt‐tRNA, *ENSBTAG00000043574* had the highest average expression difference between pre‐ and post‐puberty in muscle samples. A human obesity study demonstrated mitochondrial change in function during mid‐pubertal, suggesting its role in alteration of glucose metabolism during normal pubertal development (Fleischman, Kron, Systrom, Hrovat, & Grinspoon, 2009). As such, the high average expression difference in *ENSBTAG00000043574* could be an effect of increased glucose metabolism for energy consuming processes related to muscle growth and puberty. In addition, mitochondria functions in various cellular activities include steroid synthesis, storage of intracellular calcium ions as well as the regulation of cellular proliferation and differentiation, all processes that are involved in energy metabolism and reproduction (Wallace, 2005).

The DEx transcript with the second highest average expression difference was *ENSBTAG00000025485,* a gene of the glutathione S‐transferase (GSTA) gene family that encodes genes for processes such as detoxification and toxification mechanism (Nebert & Vasiliou, 2004). GSTA genes have been reported to be upregulated in response to oxidative stress and in many tumours (Nebert & Vasiliou, 2004). During puberty where there is an increased growth and metabolism, oxidative stress can occur due to the imbalance between antioxidant and reactive oxygen species (ROS), a by‐product of cellular metabolism (Mangel & Munch, 2005; Yu, 1994). Hence, with a fold change (FC) of 0.27, the higher pre‐pubertal expression of *ENSBTAG00000025485* could be an indication of oxidative stress. Alternatively, *ENSBTAG00000025485* could be a transcriptional repressing gene where the high pre‐pubertal expression delays the onset of puberty (Fortes, Nguyen, Porto Neto, et al., 2016). Further studies are required to investigate this hypothesis.

Majority of our top DEx transcripts are small nucleolar RNAs (snoRNAs); the *SNORA71*, *SCARNA17* and *U6*. These snoRNAs are non‐coding genes guiding the modifications of other small nuclear RNAs (Matera, Terns, & Terns, 2007). The snoRNA families are known to be involved in site‐specific RNA modification, DNA methylation and modulation of protein function (Matera et al., 2007). However, there is limited knowledge for the role of snoRNAs in muscle cells, during cattle puberty. A deletion of a snoRNA, *SNORD116* in mice resulted in post‐natal growth retardation, delayed sexual maturation, motor learning deficit and hyperphagia (Ding et al., 2008). Deletion of another class of snoRNA (HB11‐85) in humans revealed its role in energy homeostasis, growth and reproduction (de Smith et al., 2009). In vivo studies revealed the role of snoRNA U17 in regulating trafficking of cellular cholesterol, an obligate precursor for steroid hormone synthesis (Jinn et al., 2015). Altogether, snoRNAs harbour an important link between energy metabolism and reproduction but the exact mechanism in muscle during cattle puberty is not well understood. Among the three most DEx snoRNAs, only *SNORA71* had been previously discussed in literature for its additional roles in metabolic stress, a physiological process that is capable of initiating anabolic signalling for muscle growth and adaptations on energy metabolism (Ozaki, Loenneke, Buckner, & Abe, 2016; Youssef et al., 2015). The extremely high pre‐pubertal expression (FC = 0.29) could be due to an increased metabolism occurring just before the first ovulation, which is paramount for muscle growth leading to the onset of reproduction. The significant changes in the expression of *SCARNA17* (FC = 2.90) and *U6* (FC = 0.31) in muscle puberty warrant further studies.

Another non‐coding molecule, *bta‐mir‐450b* is also among our top DEx transcripts. It was significantly upregulated in the muscle tissue of Brahman heifers (FC = 2.21) after puberty. While hundreds of miRNAs have been discovered, only a few were studied in detail for their specific biological functions. There is no detailed study of *bta‐mir‐450b*. However, a previous bovine study revealed *bta‐mir‐450b* to be among the set of differentially upregulated miRNAs in the granulosa cells of pre‐ovulatory dominant follicles, suggesting this miRNA has an important role in post‐transcriptional regulation of genes involved in follicular development, during the late follicular phase of the oestrous cycle (Gebremedhn et al., 2015). Evidently, follicular development is important in the context of puberty, but there is limited information regarding the role that *bta‐mir‐450b* may play in muscle cells.

The gene *ENSBTAG00000031600* is a novel bovine gene with limited information. Another gene with high average expression difference, *ENSBTAG00000048156* is a pseudogene, which may lack protein‐coding ability. However, recent studies had revealed the regulation potential of pseudogenes on their protein‐coding counterparts (Sisu et al., 2014). The high FC of 2.02 warrants further study to explore the potential regulatory role of *ENSBTAG00000048156* in muscle puberty.

Functional enrichment analyses found significant enrichment of “extracellular region”, “extracellular space” and “extracellular region part”. These terms are generally used to describe proteins that exist outside of the cell but remains associated with it, though the term “extracellular region” can also be used to describe material that is secreted into the blood or interstitial fluid (Ashburner et al., 2000). The contraction and relaxation of skeletal muscle requires the entry of extracellular calcium (Ca^2+^) into the cytosol to activate or deactivate a series of contractile proteins (Cho, Woo, Perez, & Lee, 2017). Besides Ca^2+^ molecule, the term “extracellular region” was found to be related to amino acid transport where extracellular amino acid is required for skeletal muscle protein synthesis affecting muscle size and strength (Dickinson & Rasmussen, 2013). Protein synthesis can occur through cell surface receptors such as integrin, where binding of ECM proteins collagen induces cell proliferation and differentiation that are imperative for the growth and maintenance of muscle in livestock and humans (Allen, Merkel, & Young, 1979; Briquez, Hubbell, & Martino, 2015). The ECM components also contribute directly to the regenerative potential of muscle. In fact, it has been reported that a fibronectin‐rich fibrosis is essential to activate the proliferation of muscle satellite cells that occurs during the initial step of regeneration (Bentzinger et al., 2013). The accelerated muscle development conferred during, and continuing after puberty might involve altered gene expression to support stabilizing the ECM (Venken et al., 2007). In addition, ECM is a reservoir of growth factors and various cytokines that modulates intracellular signalling by releasing growth factors in a specific spatio‐temporal manner (Briquez et al., 2015; Kirkpatrick & Selleck, 2007). The GO term “extracellular space” applies to activin, inhibin, interleukin and insulin‐like growth factor (IGF), molecules that may interact with ECM as growth factors. Activin and inhibin are antagonists of each other their feedback to FSH release, which may contribute to puberty processes just like interleukin and IGF do (Casazza, Hanks, & Alvarez, 2010; Cole, Ahmed, Preece, Hindmarsh, & Dunger, 2015; Oksbjerg, Gondret, & Vestergaard, 2004). In short, DEx transcripts in muscle were associated with extracellular components in various aspects that may contribute to puberty.

With GO terms such as “defence response” and “inflammatory response” being significantly enriched, DEx transcripts are likely to be involved in the immune system. As concluded by previous studies, immune system is closely relevant to muscle regeneration (Yang & Hu, 2018). After a muscle injury, muscles regenerate through the activation of both innate and adaptive immune system by secreting cytokines, growth factors and other factors (Yang & Hu, 2018). The enrichment of the “complement and coagulation pathway” could be due the crosstalk between the complement system and homeostasis that are activated simultaneously during inflammation, a vital process for muscle regeneration (Markiewski, Nilsson, Nilsson Ekdahl, Mollnes, & Lambris, 2007). Besides, the increased ROS during puberty triggers the production of antioxidant, a cellular defence against the damaging effect of ROS also supports the claim that DEx transcripts have immune functions (Yu, 1994). Puberty is a phase of rapid growth (height, weight or bone size), where tissues are susceptible to changes and heightened immunity. Several studies across species reported trade‐offs between immune function and growth, implying that growth has a significant impact on the immune system (Bayyari et al., 1997; Cotter, Myatt, Benskin, & Wilson, 2008; Vijendravarma, Kraaijeveld, & Godfray, 2009). The immune system plays an important role in skeletal muscle growth, justifying the significant enrichments for terms such as “defence response” and “inflammatory response” in our samples.

It is important to note that muscle function is not confined to locomotion and posture. Skeletal muscle has been firmly established as an endocrine organ in recent years, and the cytokines or proteins released are called myokines (Lightfoot & Cooper, 2016). Subsequent to its release, myokines exert auto‐, para‐ and/or endocrine effects on various organs. For instance the myokine interleukin‐6 (IL‐6) have been reported to enhance the intestinal L‐cells and pancreatic α‐cells insulin secretion on exercise (Ellingsgaard et al., 2011). Again, under exercise, the increased mRNA and protein level of brain‐derived neurotrophic factor (BDNF) in skeletal muscle enhances fat oxidation through activation of AMPK (AMP‐dependent kinase) signalling (Matthews et al., 2009). Adding to that, physical exercises have been proven to increase the release of adrenaline, growth hormone and prolactin from skeletal muscles (Nieman, 2003). While there is limited finding regarding the release of puberty‐related hormone from skeletal muscle, at this point, the role of skeletal muscle as an endocrine organ is reaffirmed, explaining the significant enrichment of “hormone activity.” Our result is further validated by a puberty study involving LDM from Brangus bull, where the GO term “hormone activity” is also significantly enriched (Canovas et al., 2014). Our DEx transcripts that were associated with “hormone activity” include apelin, inhibin, natriuretic peptide precursor C and oxytocin (these were downregulated); and prolactin and neurotensin, which were upregulated post‐puberty. These transcripts probably respond to progesterone signalling in the muscle, as the presence of this hormone is an important difference between pre‐ and post‐pubertal heifers in our experimental design.

It is worth noticing that transcripts in “retinol metabolism” and “steroid hormone synthesis” pathways were DEx in our samples. Retinol, commonly known as Vitamin A, has been reported as essential for both male and female reproduction as its interference with steroid hormone synthesis can cause impairment in reproduction, growth and development (Clagett‐Dame & DeLuca, 2002; Sanderson, 2006). The direct role of these pathways to muscle puberty is yet to be identified.

In the light of identifying important genes in our muscle samples, a gene co‐expression network was predicted by PCIT as well as a PPI network analysed by STRING (Figure 1). We identified six hub genes; *CDC5L, MYC, TCF3, RUNX2, ATF2* and *CREB1* based on their high connectivity*.* The role of each hub gene is discussed the following paragraphs.

Cell cycle and cell proliferation is obviously important to the growth and development of complex organisms. One of our hub genes, *CDC5L* has a crucial role in cell cycle by regulating G2 progression and mitotic entry as well as gene transcription (Lei, Shen, Xu, & Bernstein, 2000). Higher levels of *CDC5L* ensure correct cell division by checking that DNA is correctly replicated and not damaged (G2/M checkpoint) (Mourot et al., 2006). It is also responsible for mRNA splicing with a high conservation across species (Ajuh & Lamond, 2003). Therefore, this gene might be regulating its predicted targets in the derived network, influencing cell cycle progression and therefore muscle growth during puberty.

Likewise*, MYC* also plays a significant role in cell cycle regulation. Commonly known as a proto‐oncogene, *MYC* is responsible for cell cycle control and cellular transformation (Bretones, Delgado, & Leon, 2015). This gene can regulate the mitochondrial metabolic network transcriptome enabling a rapid cell cycle entry, presumably during high metabolic periods such as puberty (Morrish, Neretti, Sedivy, & Hockenbery, 2008). A mice study suggested that *MYC* promotes muscle mass through the maintenance of myoblast in a proliferative state and also plays a role in differentiation and growth of muscle tissue through androgen receptors (Rana, Lee, Zajac, & MacLean, 2014). It is likely that *MYC* also functions in a similar pattern in our bovine muscle samples. In addition, *MYC* was identified as a gene with pleiotropic effects related to fertility traits, production and health in beef cattle (Fonseca et al., 2018)*.* It regulates genes associated with the lactation cycle, meat and carcass traits, as well as reproductive biology (Bionaz & Loor, 2008; Oh, Kim, Lee, & Song, 2015; Zhang, Chu, et al., 2017). In short, the *MYC* gene plays an important regulatory role in cell cycle, metabolism, growth and fertility traits in beef cattle that together may contribute to how puberty regulates to muscular growth.

**FIGURE 2:**
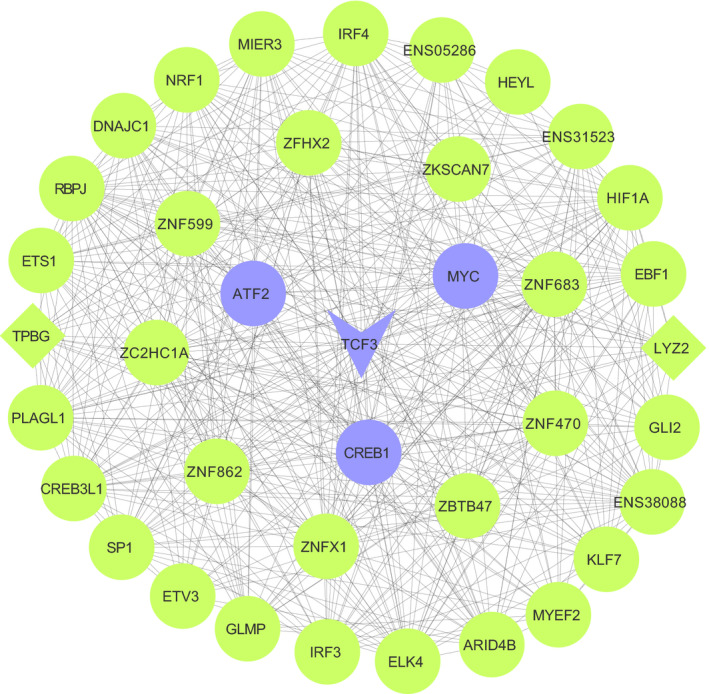
Gene co‐expression subnetwork showing all direct neighbours of *TCF3*. V‐shape represents key TF, diamond shape represents DEx genes, whereas round shape are other genes. Genes in purple are hub genes whereas genes in green are non‐hub genes. Note that all the genes in the subnetwork are TFs. Gene subnetwork created by Cytoscape. TF, transcription factor

A recent study revealed that *TCF3*, another hub gene identified in our muscle samples, regulates *MYC* (Shah, Rennoll, Raup‐Konsavage, & Yochum, 2015). *TCF3* represses *MYC* expression in colorectal cancer (Shah et al., 2015). Many genetic studies revealed *TCF3* as a transcriptional repressor across systems (Cole, Johnstone, Newman, Kagey, & Young, 2008; Merrill et al., 2004; Yi et al., 2011). For example *TCF3* acts as a transcriptional repressor during the development of hypothalamic‐pituitary axis in mice and humans (Gaston‐Massuet et al., 2016). *TCF7L1* is the promoter for *TCF3* and the term *TCF7L1* is commonly used to refer to the gene *TCF3* (Cadigan & Waterman, 2012). With evidence supporting its repressor effect on the hypothalamic‐pituitary axis that is integral to puberty onset, *TCF3* is likely to be pubertal repressive either by directly impacting the HPG axis or through the regulation of puberty‐related genes. The higher pre‐pubertal expression of *TCF3* (FC = 0.94) in our samples could be repressing the HPG axis delaying puberty onset. On top of being identified as a hub gene, *TCF3* is also a key TF with the list of DEx transcripts enriched for binding sites specific to *TCF3*, which further validates its importance in our muscle samples. Furthermore, *TCF3* has three other hub genes as direct targets; *ATF2, MYC* and *CREB1,* emphasizing its vast influence in the gene network *(*Figure 2)*.* As mentioned previously, *TCF3* can also be a gene activator and this is evidenced by its role in enhancing osteogenesis both in vitro and in vivo, making it a positive regulator of osteogenesis (Liu et al., 2013), while it could be an activator for the HPG axis.

Osteogenesis is the formation of osteoblast that is responsible for synthesis and mineralization of bone. With growth in height being one of the prominent changes in puberty, osteogenesis‐related genes are therefore important in our study. In fact, one of our hub genes, *RUNX2* is a well‐established TF in multiple stages of skeletal development (Komori, 2002). During osteogenesis, *RUNX2* determines the lineage of osteoblasts from multipotent mesenchymal cells, enhances osteoblast differentiation at an early stage, and inhibits osteoblast differentiation at a late stage (Komori, 2002). The longitudinal growth of long bones occurs at epiphyseal plate and *RUNX2* was reported to be responsible for the epiphyseal maturation, particularly during puberty (Emons et al., 2011; Milz, Boszczyk, & Putz, 2002). In short, *RUNX2* is an important TF for osteoblasts. The higher pre‐pubertal expression of *RUNX2* (FC = 0.85) in our samples could be an upregulation for osteoblast formation required for bone growth during puberty, although this key TF is not a DEx gene. Although the skeletal and muscular systems are closely related, it remains unclear whether *RUNX2* has a role in myogenesis during puberty onset. It is possible to hypothesize that *RUNX2* produced by muscle cells is perceived by bone cells and influences skeletal growth as well as epiphyseal plate ossification during puberty.

Two of our hub genes, *ATF2* and *CREB1*, are members of the ATF/CREB family with diverse functions in controlling cell proliferation and apoptosis (Persengiev & Green, 2003). In a previous study, *ATF2* was highly expressed in bovine embryonic cells suggesting its possible role in transcriptional regulation during early genome activation (Vigneault, McGraw, & Sirard, 2009). *ATF2* is an important regulator of homeostasis and cell‐fate decisions as well as a positive regulator of adipocyte differentiation and fat storage, implying it is important for development and metabolism (Gozdecka & Breitwieser, 2012; Maekawa, Jin, & Ishii, 2010; Okamura, Shimizu, Nagao, Ueda, & Ishii, 2007). The slight change in expression of *ATF2* (FC = 0.99) could implies its role in homeostasis regulation in muscle, regardless of puberty.

The gene *CREB1* is also metabolically important as it mediates transcriptional responses to different stimuli such as hormones, growth factors and neurotransmitters (Steven et al., 2017). Adipose tissue coordinates the systemic insulin and modulates energy balance through *CREB1*, implying *CREB1* plays an important role in glucose homeostasis and energy metabolism (Qi et al., 2009). *CREB1* was identified as one of the most critical genes in bovine intramuscular fats (IMF), likely to be coordinating glucose homeostasis and metabolism‐related genes (Zhang, Wang, et al., 2017). Additionally, *CREB* transcription factors also induce genes that regulate inflammation and vascular remodelling that are integral for skeletal muscle regeneration (Ichiki, 2006). Similar to *ATF2,* the change in the expression of *CREB1* (FC = 1.12) could implies its role in homeostasis that occurs throughout an organism life. Taken together, *CREB1* could be regulating the glucose homeostasis and energy metabolism in bovine IMF as well as inflammation in our muscle samples: all processes that are important for muscle growth during puberty as discussed earlier.

Despite their important roles in controlling gene expression, TFs are often lowly expressed and/or not DEx (Vaquerizas, Kummerfeld, Teichmann, & Luscombe, 2009). The interactions between TFs are paramount for tissue remodelling and temporal changes in gene expression but these vital changes are sometimes overlooked in differential expression analyses (Ravasi et al., 2010). RIF analyses of our muscle samples revealed 48 key regulators and nearly half of them (46%) belong to the zinc finger family (ZNF). This is a high percentage when compared with our previous findings; 26% ZNF in the hypothalamus, 28% in ovaries, 22% in the pituitary gland and 23% in liver samples (Fortes, Nguyen, Weller, et al., 2016; Nguyen, Reverter, et al., 2018; Nguyen et al., 2017). Although there is limited evidence regarding the role of ZNF in skeletal muscle, the ZNF members have well established role in puberty onset. Many studies in women revealed the association between single‐nucleotide polymorphism located near *ZNF462* and *ZNF483* and age of puberty (Chen et al., 2012; Perry et al., 2009). An elevation of *ZNF217* expression was observed in pre‐pubertal mice, which then declined abruptly before puberty and remained low after puberty (Abreu et al., 2013). Another study reported a decrease in *ZNF573* in peri‐pubertal female monkeys (Lomniczi et al., 2015). Two high RIF genes, *ZNF419* and *ENSBTAG00000038926* had a lower pre‐pubertal expression, raising the possibility that their reduced expression releases the puberty “brake” rendering the pulsatile release of GnRH. This possibility is in frame with the theoretical puberty “brake” proposed by Ojeda and his team (Lomniczi et al., 2015). According to this theory, ZNFs epigenetically repress a gene network that controls the pulsatile release of GnRH (Lomniczi et al., 2015). Our study not only contributes to the growing body of evidence supporting the influence of ZNF gene on puberty onset, it also indicates that more considerations should be given to skeletal muscle for its role in puberty.

Aside from the ZNF genes, the bHLH family is another major group of TFs. From our analysis, five key bHLH TFs were identified; *TCF3, HES4, TAL1, SREBF2* and *MXD4*. In general, the bHLH genes are important in regulating Sertoli cells for normal testicular function and spermatogenesis (Chaudhary, Cupp, & Skinner, 1997; Tang et al., 2014). Loss of function of a bHLH (*Nhlh2*) lead to a disruption to the hypothalamic‐pituitary axis in mice, implying its role in the onset of puberty and regulation of body metabolism (Good et al., 1997). The role of these genes in muscle merits further discussion (see below) and future work.

In addition to classifying as a key TF, *HES4* is a DEx gene in our muscle samples. Signalling by genes of the *Hes* family is important to mediate cell‐cell communication, differentiation and steroidogenesis in ovarian granulosa cells (Kageyama, Ohtsuka, & Kobayashi, 2007; Prasasya & Mayo, 2017). Overexpression of *HES4* leads to increased expression of *RUNX2*, one of our hub genes, which is critical to osteoblast differentiation (McManus et al., 2017). In our muscle samples, *HES4* is upregulated after puberty and this change in expression might be a response to steroidogenesis and progesterone signalling, post‐puberty, and this change could be vital for regulating muscle and bone growth during puberty.

The *TAL1* gene is more commonly known as *SCL/TAL1* (stem cell leukaemia/T‐cell acute lymphoblastic leukaemia) and it is an essential TF in normal and malignant hematopoiesis, (i.e. the production of blood cells) (Porcher, Chagraoui, & Kristiansen, 2017). It also has a distinct role in the formation of intracellular junctions and the maintenance of heart muscle (Schumacher, Bloomekatz, Garavito‐Aguilar, & Yelon, 2013). Bone marrow is an active site for blood production and the entire skeleton remains hematopoietically active until puberty, when only a few sites remain active (Prabhakar, Ershler, & Longo, 2009). Therefore, the upregulation of *TAL1* (FC = 1.65) in our samples could be imperative for maintaining the changing hematopoietic ability in bones as well as the identity of growing skeletal muscle during puberty. These emerging hypotheses need to be validated in future studies.

The SREBF (sterol regulatory element binding) family regulates cholesterogenic and lipogenic gene expression in the bovine liver and mammary glands. The isoform *SREBF2* is a TF for cholesterol biosynthesis in both dairy and beef cattle (Bommer & MacDougald, 2011; Romao, Jin, He, McAllister, & Guan, 2014). The pre‐pubertal upregulation of *SREBF2* (FC = 0.96) is likely to regulate increased lipid metabolism and biosynthesis associated with steroidogenesis and puberty.

A previous bovine study identified *MXD4* as a candidate gene associated with heart girth and hip height in Chinese Holstein cattle (Zhang, Chu, et al., 2017). A rodent study found *MXD4* to be DEx in skull and limb bones and likely to contribute to site‐specific mineralization and specification of bone development (Rawlinson et al., 2009). In short, *MXD4* may contribute to the growth spurt in puberty as it impacts on skeletal and muscle growth as well as energy metabolism.

We also identified four members of the homeobox family, *DLX5* and *EMX2, IRX2* and *HOXA6.* Homeobox genes have been well recognized as sequence‐specific TFs related to embryonic differentiation and limb development (Fjose, McGinnis, & Gehring, 1985). Certain homeobox genes, particularly the hox genes were proposed to be regulating muscle‐specific genes in mature muscle tissues (Capovilla, Kambris, & Botas, 2001). Specifically, members of the HoxA and HoxC clusters may regulate a universal aspect of myogenesis such as differentiation and region‐specific patterning during tissue remodelling in adult musculature (Goff & Tabin, 1997; Houghton & Rosenthal, 1999). In our sample, we identified *HOXA6* as a key TF that was previously reported for its involvement in skeletal morphogenesis and fundamental processes of hemopoietic progenitor cell development (Dickson, Kwasniewska, Mills, Lappin, & Thompson, 2009; Wellik, 2007). Similarly, *IRX2* is also involved in region‐specific patterning where *IRX1* and *IRX2* are co‐expressed and regulated during embryo hindlimb development in chickens (Diaz‐Hernandez, Bustamante, Galvan‐Hernandez, & Chimal‐Monroy, 2013). It is also known that *DLX5* and *EMX2* are responsible for osteoblast formation and differentiation. For instance mice lacking *DLX5* demonstrated severe craniofacial abnormalities with a delayed ossification of the cranium and abnormal osteogenesis (Acampora et al., 1999). Interestingly, *DLX5* was also reported as an indispensable regulator of *RUNX2*, one of our hub genes that was previously discussed (Lee et al., 2003; Shirakabe, Terasawa, Miyama, Shibuya, & Nishida, 2001). However, no significant correlation was observed between *DLX5* and *RUNX2* in our gene network. More recently, *DLX5* was identified for its involvement in myogenic differentiation, where overexpression of *DLX5* increased myogenic differentiation and knocking it down inhibited myogenesis (Qadir et al., 2014). It seems that the regulatory role of *DLX5* impacts on both skeletal and muscular systems. Similarly, *EMX2* plays a role in both skeletal and muscular systems. The upregulation of *EMX2* by the Wnt and BMP signalling pathways leads to differentiation of mesenchymal stem cells (MSCs) into osteoblast (Wei, Chen, Fu, & Zhang, 2019). The higher pre‐pubertal expression of *EMX2* in our samples could be an upregulation for osteoblast formation required for bone growth during puberty. Our study adds evidence to the importance of homeobox TFs in puberty, presumably in the context of osteogenesis and myogenesis.

One of our key TFs, *ZIC4* was also a significant TF in both pituitary gland and ovarian samples from the same heifers, as published before (Nguyen et al., 2017). Results from these three distinct tissues suggest that *ZIC4* could have a global regulatory role in puberty, perhaps in response to progesterone levels. A recent study in Hanwoo cattle associated *ZIC4* with differentiation of *longissimus dorsi* and *semi‐membranous* muscles, presumably through modulating muscle fate of satellite cells during myogenesis (de Las Heras‐Saldana, Chung, Lee, & Gondro, 2019). As such, it is tempting to assume that *ZIC4* is also regulating myogenesis during puberty in our LDM samples.

Promoter sequence analyses identified as overrepresented the TFBS that is specific to *TCF3*. The evidence for *TCF3* in regulating muscle gene expression that is relevant to puberty of Brahman heifers was reinforced across our different analyses: *TCF3* was a hub gene, and a significant TF according to RIF and to promoter region analyses. Despite being concluded as a gene repressor in multiple studies, *TCF3* was also reported as a positive regulator of osteogenesis. It is therefore imperative to investigate if *TCF3* has a repressive effect on the HPG axis and/or an activating effect on osteogenesis and muscular growth, all related aspects of pubertal development.

## 5 CONCLUSION

Our study highlights a few candidate genes that emerged from our differential expression and co‐expression analyses as major regulators of muscular growth and homeostasis during puberty, such as *CDC5L, MYC, TCF3, RUNX2, ATF2* and *CREB1.* The strongest evidence was for *TCF3,* a TF that was downregulated post‐puberty, was a hub gene in the co‐expression network, and a key TF in both RIF and promoter region analyses. These analyses also identified that zinc fingers, bHLH and homeobox families of TF contribute to gene regulation in muscle cells of per‐pubertal Brahman heifers. Many of these regulators are known as important for osteoblast differentiation and function, suggesting a regulatory link between muscle cells and osteoblasts, which might have a coordinated response to the hormonal stimulus that drive puberty. Despite the need of further investigation into the specific roles for the identified candidate genes, our study reveals the muscular tissue does respond to hormonal and developmental cues during puberty. The skeletal muscle should be taken into consideration as an active organ in future studies of cattle puberty.

## AUTHOR CONTRIBUTION

**Li Yieng Lau:** Data curation; Formal analysis; Investigation; Writing‐original draft. **Loan To Nguyen:** Formal analysis; Investigation. **Antonio Reverter:** Formal analysis; Supervision. **Stephen S. Moore:** Funding acquisition; Supervision. **Aaron Lynn:** Writing‐original draft. **Liam Mcbride‐Kelly:** Writing‐original draft. **Louis Phillips‐Rose:** Writing‐original draft. **Vanisha Vasudivan:** Writing‐original draft. **Yunan Ye:** Writing‐original draft. **Mackenzie Plath:** Writing‐original draft. **Rhys MacFarlane:** Writing‐original draft. **Marina R. S. Fortes:** Formal analysis; Resources; Supervision; Writing‐review & editing.

## Supporting information

Supplementary Material

## Data Availability

The data that support the findings of this study are available in FAANG repository http://data.faang.org/home, reference code for files related to this trial: UQMoore.
