# Supplementary material for: Gene regulation could be attributed to TCF3 and other key transcription factors in the muscle of pubertal heifers
Source: Vet Med Sci. 2020 May 20;6(4):695–710. doi: 10.1002/vms3.278 (PMC7738712; doi:10.1002/vms3.278)
Supplement: Supplementary file 1 — Supplementary Material [file VMS3-6-695-s001.docx]

Table S1 Summary of 431 differentially expressed (DEx) transcripts in the muscle tissue of Bos indicus Brahman heifers (P < 0.05); a comparison between the average gene expression from six post-pubertal (POST) and five pre-pubertal (PRE) Brahman heifers. Genes are ranked in ascending order of average expression difference.

| ENSB Tag^†^ | Gene |  | TF^‡^ |  | POST^§^ | PRE^§^ | Average expression difference | FC^¶^ |  |
| --- | --- | --- | --- | --- | --- | --- | --- | --- | --- |
| ENSBTAG00000025485 | ENS25485 |  | - |  | 1.95 | 7.12 | -5.17 | 0.27 |  |
| ENSBTAG00000042678 | SNORA71 |  | - |  | 2.01 | 6.94 | -4.93 | 0.29 |  |
| ENSBTAG00000044778 | U6 |  | - |  | 2.14 | 6.91 | -4.77 | 0.31 |  |
| ENSBTAG00000031600 | ENS31600 |  | - |  | 2.51 | 7.06 | -4.55 | 0.36 |  |
| ENSBTAG00000045753 | ENS45753 |  | - |  | 3.35 | 7.13 | -3.79 | 0.47 |  |
| ENSBTAG00000000644 | S100A5 |  | - |  | 3.41 | 7.18 | -3.76 | 0.47 |  |
| ENSBTAG00000045080 | SNORD5 |  | - |  | 3.25 | 7.01 | -3.76 | 0.46 |  |
| ENSBTAG00000045836 | ENS45836 |  | - |  | 4.91 | 8.63 | -3.71 | 0.57 |  |
| ENSBTAG00000045967 | LENEP |  | - |  | 2.27 | 5.97 | -3.70 | 0.38 |  |
| ENSBTAG00000007043 | ENS7043 |  | - |  | 2.12 | 5.82 | -3.70 | 0.36 |  |
| ENSBTAG00000005722 | ENS5722 |  | - |  | 2.03 | 5.70 | -3.67 | 0.36 |  |
| ENSBTAG00000044962 | ENS44962 |  | - |  | 4.91 | 8.49 | -3.59 | 0.58 |  |
| ENSBTAG00000002642 | SLC17A2 |  | - |  | 2.43 | 5.92 | -3.49 | 0.41 |  |
| ENSBTAG00000012692 | ENS12692 |  | - |  | 2.50 | 5.98 | -3.48 | 0.42 |  |
| ENSBTAG00000012540 | ENS12540 |  | - |  | 2.28 | 5.73 | -3.46 | 0.40 |  |
| ENSBTAG00000046885 | RXFP4 |  | - |  | 3.26 | 6.66 | -3.41 | 0.49 |  |
| ENSBTAG00000046521 | ENS46521 |  | - |  | 2.50 | 5.86 | -3.36 | 0.43 |  |
| ENSBTAG00000026882 | ENS26882 |  | - |  | 2.55 | 5.88 | -3.33 | 0.43 |  |
| ENSBTAG00000015836 | EXOC3L2 |  | - |  | 3.41 | 6.69 | -3.28 | 0.51 |  |
| ENSBTAG00000000229 | ENS229 |  | - |  | 3.10 | 6.37 | -3.28 | 0.49 |  |
| ENSBTAG00000042723 | U11 |  | - |  | 0.92 | 4.19 | -3.27 | 0.22 |  |
| ENSBTAG00000020685 | ENS20685 |  | TF |  | 2.92 | 6.15 | -3.23 | 0.47 |  |
| ENSBTAG00000033740 | ENS33740 |  | - |  | 0.87 | 4.08 | -3.21 | 0.21 |  |
| ENSBTAG00000014899 | TTC36 |  | - |  | 2.27 | 5.38 | -3.11 | 0.42 |  |
| ENSBTAG00000042758 | SNORD29 |  | - |  | 0.61 | 3.68 | -3.07 | 0.17 |  |
| ENSBTAG00000003515 | SNCG |  | - |  | 1.99 | 5.01 | -3.01 | 0.40 |  |
| ENSBTAG00000011655 | SAMD10 |  | - |  | 1.86 | 4.87 | -3.01 | 0.38 |  |
| ENSBTAG00000047240 | ENS47240 |  | - |  | 3.21 | 6.18 | -2.97 | 0.52 |  |
| ENSBTAG00000001392 | RDH16 |  | - |  | 2.21 | 5.17 | -2.96 | 0.43 |  |
| ENSBTAG00000033225 | CNTN4 |  | - |  | 4.81 | 7.73 | -2.92 | 0.62 |  |
| ENSBTAG00000020076 | CATHL5 |  | - |  | 5.95 | 8.86 | -2.91 | 0.67 |  |
| ENSBTAG00000021887 | DPYS |  | - |  | 2.27 | 5.16 | -2.89 | 0.44 |  |
| ENSBTAG00000009350 | PLA2G12B |  | - |  | 3.05 | 5.92 | -2.87 | 0.52 |  |
| ENSBTAG00000023411 | ENS23411 |  | - |  | 2.17 | 5.03 | -2.86 | 0.43 |  |
| ENSBTAG00000024272 | BT.37579 |  | - |  | 1.99 | 4.81 | -2.82 | 0.41 |  |
| ENSBTAG00000032198 | ENS32198 |  | - |  | 1.64 | 4.42 | -2.79 | 0.37 |  |
| ENSBTAG00000010177 | GOLT1A |  | - |  | 1.10 | 3.86 | -2.77 | 0.28 |  |
| ENSBTAG00000048226 | ENS48226 |  | - |  | 1.85 | 4.61 | -2.76 | 0.40 |  |
| ENSBTAG00000047866 | ENS47866 |  | - |  | 3.20 | 5.96 | -2.76 | 0.54 |  |
| ENSBTAG00000008026 | OXT |  | - |  | 1.79 | 4.54 | -2.75 | 0.39 |  |
| ENSBTAG00000015347 | WNT10B |  | - |  | 4.87 | 7.61 | -2.74 | 0.64 |  |
| ENSBTAG00000018634 | SH3BGRL2 |  | - |  | 1.59 | 4.33 | -2.74 | 0.37 |  |
| ENSBTAG00000043400 | U6 |  | - |  | 5.79 | 8.49 | -2.70 | 0.68 |  |
| ENSBTAG00000036061 | ENS36061 |  | - |  | 1.74 | 4.43 | -2.68 | 0.39 |  |
| ENSBTAG00000003236 | ENS3236 |  | - |  | 2.41 | 5.05 | -2.64 | 0.48 |  |
| ENSBTAG00000007816 | ENS7816 |  | - |  | 0.92 | 3.56 | -2.64 | 0.26 |  |
| ENSBTAG00000008259 | IRG1 |  | - |  | 3.40 | 6.04 | -2.64 | 0.56 |  |
| ENSBTAG00000038361 | SERPINA11 |  | - |  | 2.58 | 5.21 | -2.63 | 0.50 |  |
| ENSBTAG00000043519 | SNORD93 |  | - |  | 4.46 | 7.04 | -2.58 | 0.63 |  |
| ENSBTAG00000023007 | ENS23007 |  | - |  | 4.92 | 7.48 | -2.56 | 0.66 |  |
| ENSBTAG00000007720 | NKG2A |  | - |  | 4.91 | 7.45 | -2.55 | 0.66 |  |
| ENSBTAG00000008900 | CELA1 |  | - |  | 3.08 | 5.62 | -2.55 | 0.55 |  |
| ENSBTAG00000037856 | MGC152281 |  | - |  | 2.85 | 5.38 | -2.53 | 0.53 |  |
| ENSBTAG00000015880 | PRIMA1 |  | - |  | 6.10 | 8.62 | -2.52 | 0.71 |  |
| ENSBTAG00000008323 | SNAP25 |  | - |  | 4.49 | 7.01 | -2.52 | 0.64 |  |
| ENSBTAG00000018869 | IGSF6 |  | - |  | 2.36 | 4.87 | -2.51 | 0.48 |  |
| ENSBTAG00000047302 | ENS47302 |  | - |  | 5.25 | 7.74 | -2.50 | 0.68 |  |
| ENSBTAG00000008384 | SLC25A47 |  | - |  | 4.49 | 6.98 | -2.49 | 0.64 |  |
| ENSBTAG00000038171 | ENS38171 |  | - |  | 3.63 | 6.12 | -2.49 | 0.59 |  |
| ENSBTAG00000024545 | DCHS2 |  | - |  | 4.73 | 7.22 | -2.48 | 0.66 |  |
| ENSBTAG00000042447 | SNORD49 |  | - |  | 4.44 | 6.92 | -2.47 | 0.64 |  |
| ENSBTAG00000020790 | ENS20790 |  | - |  | 2.32 | 4.79 | -2.47 | 0.48 |  |
| ENSBTAG00000024851 | TRIM14 |  | - |  | 3.16 | 5.61 | -2.46 | 0.56 |  |
| ENSBTAG00000038073 | bta-mir-1247 |  | - |  | 6.05 | 8.50 | -2.45 | 0.71 |  |
| ENSBTAG00000045271 | ENS45271 |  | - |  | 4.64 | 7.09 | -2.45 | 0.65 |  |
| ENSBTAG00000007514 | CPNE5 |  | - |  | 5.45 | 7.89 | -2.44 | 0.69 |  |
| ENSBTAG00000044712 | bta-mir-2383 |  | - |  | 4.73 | 7.14 | -2.41 | 0.66 |  |
| ENSBTAG00000045955 | TSSK1B |  | - |  | 3.84 | 6.21 | -2.37 | 0.62 |  |
| ENSBTAG00000013973 | SERPIND1 |  | - |  | 1.96 | 4.33 | -2.36 | 0.45 |  |
| ENSBTAG00000009501 | KLKB1 |  | - |  | 2.18 | 4.54 | -2.36 | 0.48 |  |
| ENSBTAG00000017343 | COLEC10 |  | - |  | 3.72 | 6.08 | -2.36 | 0.61 |  |
| ENSBTAG00000017335 | HGFAC |  | - |  | 2.63 | 4.99 | -2.36 | 0.53 |  |
| ENSBTAG00000042234 | SNORA70 |  | - |  | 6.13 | 8.45 | -2.33 | 0.73 |  |
| ENSBTAG00000046744 | PALM3 |  | - |  | 2.65 | 4.93 | -2.28 | 0.54 |  |
| ENSBTAG00000022246 | C29H11orf86 |  | - |  | 2.58 | 4.85 | -2.27 | 0.53 |  |
| ENSBTAG00000019533 | C23H6ORF105 |  | - |  | 1.90 | 4.16 | -2.26 | 0.46 |  |
| ENSBTAG00000040337 | ENS40337 |  | - |  | 1.48 | 3.73 | -2.25 | 0.40 |  |
| ENSBTAG00000039550 | U2 |  | - |  | 2.37 | 4.61 | -2.24 | 0.51 |  |
| ENSBTAG00000000259 | CHIA |  | - |  | 1.87 | 4.11 | -2.24 | 0.45 |  |
| ENSBTAG00000046062 | ENS46062 |  | - |  | 2.41 | 4.64 | -2.23 | 0.52 |  |
| ENSBTAG00000004303 | SLC27A2 |  | - |  | 2.47 | 4.67 | -2.20 | 0.53 |  |
| ENSBTAG00000035572 | ENS35572 |  | - |  | 2.59 | 4.75 | -2.16 | 0.55 |  |
| ENSBTAG00000040070 | SCGB1D |  | - |  | 1.76 | 3.91 | -2.15 | 0.45 |  |
| ENSBTAG00000035975 | SCIMP |  | - |  | 2.64 | 4.77 | -2.13 | 0.55 |  |
| ENSBTAG00000019764 | APOA5 |  | - |  | 1.83 | 3.96 | -2.13 | 0.46 |  |
| ENSBTAG00000000070 | F13B |  | - |  | 1.84 | 3.97 | -2.12 | 0.46 |  |
| ENSBTAG00000002963 | SAA4 |  | - |  | 2.28 | 4.39 | -2.12 | 0.52 |  |
| ENSBTAG00000000085 | CYP1A2 |  | - |  | 1.74 | 3.83 | -2.09 | 0.45 |  |
| ENSBTAG00000020512 | GJB1 |  | - |  | 1.72 | 3.79 | -2.07 | 0.45 |  |
| ENSBTAG00000046506 | ENS46506 |  | - |  | 1.85 | 3.92 | -2.07 | 0.47 |  |
| ENSBTAG00000004384 | APOF |  | - |  | 1.65 | 3.71 | -2.06 | 0.44 |  |
| ENSBTAG00000001836 | ENS1836 |  | - |  | 1.65 | 3.65 | -2.01 | 0.45 |  |
| ENSBTAG00000047547 | ENS47547 |  | - |  | 0.53 | 2.53 | -2.00 | 0.21 |  |
| ENSBTAG00000017714 | C4BPB |  | - |  | 1.67 | 3.67 | -2.00 | 0.46 |  |
| ENSBTAG00000018481 | ENS18481 |  | - |  | 2.18 | 4.17 | -2.00 | 0.52 |  |
| ENSBTAG00000018069 | TNFSF15 |  | - |  | 2.04 | 4.02 | -1.99 | 0.51 |  |
| ENSBTAG00000045327 | ACA64 |  | - |  | 2.36 | 4.35 | -1.99 | 0.54 |  |
| ENSBTAG00000015047 | ENS15047 |  | - |  | 1.90 | 3.88 | -1.98 | 0.49 |  |
| ENSBTAG00000042504 | SNORD78 |  | - |  | 1.93 | 3.90 | -1.97 | 0.49 |  |
| ENSBTAG00000013429 | CLEC4G |  | - |  | 1.57 | 3.52 | -1.95 | 0.45 |  |
| ENSBTAG00000020558 | APOC2 |  | - |  | 1.45 | 3.38 | -1.93 | 0.43 |  |
| ENSBTAG00000048080 | ENS48080 |  | - |  | 1.50 | 3.38 | -1.88 | 0.44 |  |
| ENSBTAG00000013598 | RSPO2 |  | - |  | 1.75 | 3.61 | -1.87 | 0.48 |  |
| ENSBTAG00000008127 | RBP5 |  | - |  | 2.01 | 3.87 | -1.87 | 0.52 |  |
| ENSBTAG00000020049 | CABP1 |  | - |  | 2.76 | 4.62 | -1.86 | 0.60 |  |
| ENSBTAG00000042217 | SNORD77 |  | - |  | 2.03 | 3.88 | -1.85 | 0.52 |  |
| ENSBTAG00000002255 | BHMT |  | - |  | 2.07 | 3.89 | -1.83 | 0.53 |  |
| ENSBTAG00000024874 | ENS24874 |  | - |  | 1.11 | 2.91 | -1.81 | 0.38 |  |
| ENSBTAG00000037890 | ENS37890 |  | - |  | 1.86 | 3.65 | -1.80 | 0.51 |  |
| ENSBTAG00000010641 | APH1B |  | - |  | 0.84 | 2.64 | -1.79 | 0.32 |  |
| ENSBTAG00000048206 | ENS48206 |  | - |  | 1.85 | 3.64 | -1.78 | 0.51 |  |
| ENSBTAG00000000123 | HAO2 |  | - |  | 1.93 | 3.71 | -1.77 | 0.52 |  |
| ENSBTAG00000018926 | LEAP2 |  | - |  | 1.24 | 2.99 | -1.75 | 0.41 |  |
| ENSBTAG00000013134 | SOST |  | - |  | 2.23 | 3.97 | -1.74 | 0.56 |  |
| ENSBTAG00000043580 | ENS43580 |  | - |  | 0.93 | 2.66 | -1.73 | 0.35 |  |
| ENSBTAG00000012640 | S100A8 |  | - |  | 1.11 | 2.83 | -1.72 | 0.39 |  |
| ENSBTAG00000004224 | C19orf38 |  | - |  | 1.89 | 3.61 | -1.72 | 0.52 |  |
| ENSBTAG00000005251 | ENS5251 |  | - |  | 2.14 | 3.85 | -1.72 | 0.56 |  |
| ENSBTAG00000042180 | SNORD47 |  | - |  | 0.35 | 2.06 | -1.71 | 0.17 |  |
| ENSBTAG00000013736 | PROM1 |  | - |  | 2.90 | 4.61 | -1.71 | 0.63 |  |
| ENSBTAG00000012164 | CP |  | - |  | 2.20 | 3.90 | -1.70 | 0.56 |  |
| ENSBTAG00000046666 | TTC9B |  | - |  | 2.29 | 3.98 | -1.69 | 0.58 |  |
| ENSBTAG00000042950 | SNORD45 |  | - |  | 2.28 | 3.97 | -1.69 | 0.57 |  |
| ENSBTAG00000037634 | ENS37634 |  | - |  | 2.05 | 3.74 | -1.69 | 0.55 |  |
| ENSBTAG00000047342 | ENS47342 |  | - |  | 2.71 | 4.38 | -1.67 | 0.62 |  |
| ENSBTAG00000021649 | ENS21649 |  | - |  | 1.48 | 3.13 | -1.65 | 0.47 |  |
| ENSBTAG00000001152 | TLE6 |  | - |  | 2.28 | 3.92 | -1.64 | 0.58 |  |
| ENSBTAG00000046386 | SERTM1 |  | - |  | 0.90 | 2.54 | -1.63 | 0.35 |  |
| ENSBTAG00000005647 | GPR132 |  | - |  | 2.88 | 4.51 | -1.63 | 0.64 |  |
| ENSBTAG00000023032 | SFTPA1 |  | - |  | 1.81 | 3.43 | -1.62 | 0.53 |  |
| ENSBTAG00000014250 | ZC3HAV1L |  | - |  | 1.61 | 3.22 | -1.60 | 0.50 |  |
| ENSBTAG00000001981 | SLC6A17 |  | - |  | 2.46 | 4.06 | -1.60 | 0.61 |  |
| ENSBTAG00000048287 | ENS48287 |  | - |  | 1.00 | 2.59 | -1.59 | 0.39 |  |
| ENSBTAG00000006231 | ACSBG1 |  | - |  | 2.74 | 4.30 | -1.56 | 0.64 |  |
| ENSBTAG00000013333 | GYS2 |  | - |  | 2.06 | 3.62 | -1.56 | 0.57 |  |
| ENSBTAG00000000011 | TDH |  | - |  | 1.76 | 3.31 | -1.55 | 0.53 |  |
| ENSBTAG00000010531 | CYP1B1 |  | - |  | 2.34 | 3.89 | -1.55 | 0.60 |  |
| ENSBTAG00000001517 | KRT18 |  | - |  | 1.20 | 2.74 | -1.54 | 0.44 |  |
| ENSBTAG00000014517 | KLB |  | - |  | 2.65 | 4.18 | -1.53 | 0.63 |  |
| ENSBTAG00000046277 | RGS4 |  | - |  | 2.78 | 4.31 | -1.53 | 0.65 |  |
| ENSBTAG00000004170 | ENS4170 |  | - |  | 2.60 | 4.13 | -1.52 | 0.63 |  |
| ENSBTAG00000046628 | LYZ3 |  | - |  | 3.03 | 4.55 | -1.52 | 0.67 |  |
| ENSBTAG00000043000 | SNORA1 |  | - |  | 2.22 | 3.74 | -1.52 | 0.59 |  |
| ENSBTAG00000001658 | AKR1D1 |  | - |  | 2.63 | 4.14 | -1.51 | 0.64 |  |
| ENSBTAG00000039477 | TPBG |  | - |  | 1.83 | 3.33 | -1.50 | 0.55 |  |
| ENSBTAG00000022471 | TTPA |  | - |  | 2.80 | 4.30 | -1.50 | 0.65 |  |
| ENSBTAG00000042963 | SNORA8 |  | - |  | 0.94 | 2.43 | -1.49 | 0.39 |  |
| ENSBTAG00000010236 | CIDEB |  | - |  | 1.32 | 2.80 | -1.48 | 0.47 |  |
| ENSBTAG00000047030 | ENS47030 |  | - |  | 0.93 | 2.40 | -1.48 | 0.39 |  |
| ENSBTAG00000042974 | SNORD24 |  | - |  | 0.68 | 2.15 | -1.47 | 0.32 |  |
| ENSBTAG00000019993 | APLN |  | - |  | 1.00 | 2.46 | -1.45 | 0.41 |  |
| ENSBTAG00000047795 | ENS47795 |  | - |  | 0.77 | 2.18 | -1.41 | 0.35 |  |
| ENSBTAG00000043086 | Y_RNA |  | - |  | 1.41 | 2.78 | -1.37 | 0.51 |  |
| ENSBTAG00000043378 | SNORD81 |  | - |  | 0.82 | 2.18 | -1.35 | 0.38 |  |
| ENSBTAG00000048120 | 7SK |  | - |  | 0.99 | 2.32 | -1.33 | 0.43 |  |
| ENSBTAG00000003898 | HMGCS2 |  | - |  | 1.62 | 2.95 | -1.32 | 0.55 |  |
| ENSBTAG00000047431 | SNORA42 |  | - |  | 0.69 | 1.96 | -1.27 | 0.35 |  |
| ENSBTAG00000024490 | ULBP27 |  | - |  | 0.93 | 2.19 | -1.26 | 0.42 |  |
| ENSBTAG00000022372 | ENS22372 |  | - |  | 1.24 | 2.49 | -1.26 | 0.50 |  |
| ENSBTAG00000047772 | FBXO48 |  | - |  | 0.68 | 1.92 | -1.24 | 0.35 |  |
| ENSBTAG00000035654 | DYNLT1 |  | - |  | 1.65 | 2.87 | -1.22 | 0.57 |  |
| ENSBTAG00000032350 | ENS32350 |  | - |  | 1.61 | 2.76 | -1.15 | 0.58 |  |
| ENSBTAG00000005099 | ENS5099 |  | - |  | 1.63 | 2.77 | -1.14 | 0.59 |  |
| ENSBTAG00000001852 | BREH1 |  | - |  | 0.93 | 2.05 | -1.12 | 0.45 |  |
| ENSBTAG00000029828 | bta-mir-421 |  | - |  | 0.96 | 2.08 | -1.11 | 0.46 |  |
| ENSBTAG00000031647 | LRG1 |  | - |  | 1.39 | 2.50 | -1.11 | 0.56 |  |
| ENSBTAG00000022394 | SAA1 |  | - |  | 1.66 | 2.75 | -1.08 | 0.60 |  |
| ENSBTAG00000017155 | TRIM32 |  | - |  | 1.30 | 2.37 | -1.08 | 0.55 |  |
| ENSBTAG00000043969 | CALN1 |  | - |  | 1.82 | 2.89 | -1.07 | 0.63 |  |
| ENSBTAG00000004003 | F9 |  | - |  | 1.64 | 2.70 | -1.07 | 0.61 |  |
| ENSBTAG00000007049 | MBL |  | - |  | 1.78 | 2.84 | -1.05 | 0.63 |  |
| ENSBTAG00000008238 | S100A7 |  | - |  | 1.35 | 2.38 | -1.03 | 0.57 |  |
| ENSBTAG00000005033 | FAM57B |  | - |  | 1.77 | 2.80 | -1.02 | 0.63 |  |
| ENSBTAG00000047040 | SERPINA3-6 |  | - |  | 1.55 | 2.56 | -1.02 | 0.61 |  |
| ENSBTAG00000005479 | LIPC |  | - |  | 1.84 | 2.85 | -1.01 | 0.65 |  |
| ENSBTAG00000002799 | SLC39A5 |  | - |  | 1.82 | 2.82 | -1.00 | 0.65 |  |
| ENSBTAG00000013020 | ENS13020 |  | - |  | 1.60 | 2.59 | -0.99 | 0.62 |  |
| ENSBTAG00000022329 | ENS22329 |  | - |  | 1.77 | 2.75 | -0.98 | 0.64 |  |
| ENSBTAG00000003774 | C8A |  | - |  | 1.55 | 2.53 | -0.98 | 0.61 |  |
| ENSBTAG00000027610 | RPL36A |  | - |  | 0.78 | 1.72 | -0.94 | 0.45 |  |
| ENSBTAG00000015402 | GREB1 |  | - |  | 1.23 | 2.15 | -0.92 | 0.57 |  |
| ENSBTAG00000047317 | CL43 |  | - |  | 1.49 | 2.41 | -0.91 | 0.62 |  |
| ENSBTAG00000031388 | DYX1C1 |  | - |  | 1.46 | 2.37 | -0.91 | 0.62 |  |
| ENSBTAG00000026848 | ENS26848 |  | - |  | 1.07 | 1.98 | -0.91 | 0.54 |  |
| ENSBTAG00000032217 | ENS32217 |  | - |  | 0.89 | 1.79 | -0.91 | 0.50 |  |
| ENSBTAG00000013907 | CRP |  | - |  | 1.73 | 2.60 | -0.87 | 0.67 |  |
| ENSBTAG00000009735 | A1BG |  | - |  | 1.42 | 2.29 | -0.87 | 0.62 |  |
| ENSBTAG00000019616 | APCS |  | - |  | 1.23 | 2.08 | -0.86 | 0.59 |  |
| ENSBTAG00000032017 | INHBB |  | - |  | 1.09 | 1.94 | -0.85 | 0.56 |  |
| ENSBTAG00000000859 | SLC38A1 |  | - |  | 0.57 | 1.37 | -0.81 | 0.42 |  |
| ENSBTAG00000024983 | FBXO47 |  | - |  | 0.80 | 1.61 | -0.81 | 0.50 |  |
| ENSBTAG00000036111 | ENS36111 |  | - |  | 0.67 | 1.46 | -0.80 | 0.46 |  |
| ENSBTAG00000005596 | IGFBP2 |  | - |  | 1.17 | 1.94 | -0.77 | 0.60 |  |
| ENSBTAG00000047902 | ENS47902 |  | - |  | 0.82 | 1.56 | -0.74 | 0.53 |  |
| ENSBTAG00000000396 | PIM1 |  | - |  | 0.67 | 1.41 | -0.74 | 0.48 |  |
| ENSBTAG00000018161 | TBX18 |  | TF |  | 0.73 | 1.45 | -0.72 | 0.50 |  |
| ENSBTAG00000047449 | ENS47449 |  | - |  | 1.18 | 1.90 | -0.72 | 0.62 |  |
| ENSBTAG00000037452 | ENS37452 |  | - |  | 1.16 | 1.88 | -0.72 | 0.62 |  |
| ENSBTAG00000003253 | NPPC |  | - |  | 1.11 | 1.80 | -0.70 | 0.62 |  |
| ENSBTAG00000015164 | SLC27A5 |  | - |  | 1.14 | 1.83 | -0.70 | 0.62 |  |
| ENSBTAG00000016391 | ENS16391 |  | - |  | 1.09 | 1.77 | -0.68 | 0.62 |  |
| ENSBTAG00000038888 | ZBTB9 |  | - |  | 0.32 | 0.99 | -0.68 | 0.32 |  |
| ENSBTAG00000000533 | ZNF684 |  | TF |  | 1.13 | 1.80 | -0.68 | 0.63 |  |
| ENSBTAG00000040167 | ENS40167 |  | - |  | 0.58 | 1.25 | -0.67 | 0.46 |  |
| ENSBTAG00000038698 | ENS38698 |  | - |  | 1.14 | 1.80 | -0.65 | 0.63 |  |
| ENSBTAG00000005382 | CCDC85B |  | - |  | 0.95 | 1.59 | -0.65 | 0.60 |  |
| ENSBTAG00000017242 | FADS6 |  | - |  | 1.01 | 1.65 | -0.64 | 0.61 |  |
| ENSBTAG00000020597 | FMO3 |  | - |  | 1.07 | 1.69 | -0.63 | 0.63 |  |
| ENSBTAG00000015387 | RAB40B |  | - |  | 1.13 | 1.76 | -0.63 | 0.64 |  |
| ENSBTAG00000006606 | FAM101B |  | - |  | 1.23 | 1.85 | -0.62 | 0.66 |  |
| ENSBTAG00000032151 | CCDC73 |  | - |  | 1.19 | 1.81 | -0.62 | 0.66 |  |
| ENSBTAG00000006424 | TNNI3 |  | - |  | 0.96 | 1.58 | -0.62 | 0.61 |  |
| ENSBTAG00000014217 | HHEX |  | TF |  | 0.62 | 1.22 | -0.60 | 0.51 |  |
| ENSBTAG00000024675 | CYSLTR1 |  | - |  | 0.70 | 1.27 | -0.57 | 0.55 |  |
| ENSBTAG00000045948 | CSF2RA |  | - |  | 0.43 | 1.00 | -0.57 | 0.43 |  |
| ENSBTAG00000013294 | DYRK3 |  | - |  | 0.55 | 1.11 | -0.57 | 0.50 |  |
| ENSBTAG00000008441 | SOCS3 |  | - |  | 0.56 | 1.12 | -0.56 | 0.50 |  |
| ENSBTAG00000017405 | RORC |  | TF |  | 0.69 | 1.24 | -0.55 | 0.56 |  |
| ENSBTAG00000008222 | FAM86A |  | - |  | 0.29 | 0.82 | -0.53 | 0.35 |  |
| ENSBTAG00000002914 | GALNTL4 |  | - |  | 0.67 | 1.18 | -0.51 | 0.57 |  |
| ENSBTAG00000008913 | TMEM98 |  | - |  | 0.61 | 1.10 | -0.49 | 0.55 |  |
| ENSBTAG00000000061 | PCDH7 |  | - |  | 0.15 | 0.63 | -0.49 | 0.24 |  |
| ENSBTAG00000016494 | RELT |  | - |  | 0.59 | 1.08 | -0.49 | 0.55 |  |
| ENSBTAG00000003894 | NDRG4 |  | - |  | 0.49 | 0.97 | -0.49 | 0.51 |  |
| ENSBTAG00000007507 | FAAH |  | - |  | 0.59 | 1.07 | -0.48 | 0.55 |  |
| ENSBTAG00000046544 | SLC7A10 |  | - |  | 0.36 | 0.83 | -0.47 | 0.43 |  |
| ENSBTAG00000009573 | C11orf49 |  | - |  | 0.11 | 0.58 | -0.47 | 0.19 |  |
| ENSBTAG00000033365 | C9H6orf186 |  | - |  | 0.56 | 1.03 | -0.47 | 0.54 |  |
| ENSBTAG00000014135 | WDR17 |  | - |  | 0.70 | 1.16 | -0.45 | 0.60 |  |
| ENSBTAG00000007402 | ZFAND2A |  | - |  | 0.46 | 0.91 | -0.45 | 0.51 |  |
| ENSBTAG00000009493 | BCL3 |  | - |  | 0.40 | 0.84 | -0.44 | 0.48 |  |
| ENSBTAG00000020209 | TIGD7 |  | - |  | 0.68 | 1.12 | -0.44 | 0.61 |  |
| ENSBTAG00000047361 | ENS47361 |  | - |  | 0.59 | 1.03 | -0.44 | 0.57 |  |
| ENSBTAG00000002878 | CHRD |  | - |  | 0.67 | 1.11 | -0.44 | 0.60 |  |
| ENSBTAG00000001886 | FAM122B |  | - |  | 0.71 | 1.14 | -0.43 | 0.62 |  |
| ENSBTAG00000016987 | C2orf44 |  | - |  | 0.60 | 1.03 | -0.43 | 0.58 |  |
| ENSBTAG00000030540 | ZDHHC12 |  | - |  | 0.31 | 0.74 | -0.43 | 0.42 |  |
| ENSBTAG00000008711 | BOK |  | - |  | 0.54 | 0.97 | -0.43 | 0.56 |  |
| ENSBTAG00000038710 | 0.039 |  | TF |  | 0.84 | 0.34 | 0.50 | 2.47 |  |
| ENSBTAG00000003200 | 0.029 |  | - |  | 1.18 | 0.67 | 0.50 | 1.76 |  |
| ENSBTAG00000007834 | 0.138 |  | - |  | 1.09 | 0.58 | 0.51 | 1.88 |  |
| ENSBTAG00000021219 | 0.409 |  | - |  | 1.21 | 0.70 | 0.51 | 1.73 |  |
| ENSBTAG00000017670 | 0.139 |  | - |  | 1.13 | 0.61 | 0.51 | 1.85 |  |
| ENSBTAG00000008726 | 0.124 |  | - |  | 1.19 | 0.67 | 0.52 | 1.78 |  |
| ENSBTAG00000047357 | 0.163 |  | - |  | 1.24 | 0.71 | 0.53 | 1.75 |  |
| ENSBTAG00000014252 | 0.055 |  | - |  | 0.95 | 0.42 | 0.53 | 2.26 |  |
| ENSBTAG00000007881 | 0.161 |  | - |  | 1.13 | 0.58 | 0.55 | 1.95 |  |
| ENSBTAG00000013131 | 0.032 |  | - |  | 0.92 | 0.36 | 0.56 | 2.56 |  |
| ENSBTAG00000001346 | 0.152 |  | - |  | 1.02 | 0.45 | 0.57 | 2.27 |  |
| ENSBTAG00000012184 | 0.011 |  | - |  | 1.00 | 0.41 | 0.59 | 2.44 |  |
| ENSBTAG00000048049 | 0.126 |  | - |  | 1.22 | 0.62 | 0.60 | 1.97 |  |
| ENSBTAG00000035129 | 0.017 |  | - |  | 0.96 | 0.35 | 0.61 | 2.74 |  |
| ENSBTAG00000016430 | 0.066 |  | - |  | 1.12 | 0.50 | 0.62 | 2.24 |  |
| ENSBTAG00000008551 | 0.127 |  | - |  | 1.23 | 0.60 | 0.63 | 2.05 |  |
| ENSBTAG00000021437 | 0.265 |  | - |  | 1.79 | 1.14 | 0.65 | 1.57 |  |
| ENSBTAG00000018806 | 0.085 |  | - |  | 1.68 | 1.03 | 0.65 | 1.63 |  |
| ENSBTAG00000020803 | 0.062 |  | - |  | 1.56 | 0.91 | 0.65 | 1.71 |  |
| ENSBTAG00000017020 | 0.261 |  | - |  | 1.82 | 1.17 | 0.65 | 1.56 |  |
| ENSBTAG00000000128 | 0.381 |  | - |  | 1.41 | 0.75 | 0.66 | 1.88 |  |
| ENSBTAG00000031774 | 0.202 |  | - |  | 1.21 | 0.55 | 0.66 | 2.20 |  |
| ENSBTAG00000005244 | 0.289 |  | - |  | 1.57 | 0.90 | 0.66 | 1.74 |  |
| ENSBTAG00000020099 | 0.280 |  | - |  | 1.76 | 1.10 | 0.67 | 1.60 |  |
| ENSBTAG00000003212 | 0.150 |  | - |  | 1.67 | 1.00 | 0.67 | 1.67 |  |
| ENSBTAG00000001079 | 0.328 |  | - |  | 1.49 | 0.82 | 0.67 | 1.82 |  |
| ENSBTAG00000017333 | 0.378 |  | - |  | 1.88 | 1.21 | 0.68 | 1.55 |  |
| ENSBTAG00000014705 | 0.136 |  | TF |  | 1.73 | 1.05 | 0.68 | 1.65 |  |
| ENSBTAG00000009002 | 0.014 |  | - |  | 1.63 | 0.94 | 0.69 | 1.73 |  |
| ENSBTAG00000017529 | 0.188 |  | - |  | 1.75 | 1.06 | 0.69 | 1.65 |  |
| ENSBTAG00000000835 | 0.271 |  | - |  | 1.92 | 1.22 | 0.70 | 1.57 |  |
| ENSBTAG00000026070 | 0.441 |  | - |  | 1.67 | 0.97 | 0.70 | 1.72 |  |
| ENSBTAG00000001139 | 0.310 |  | - |  | 1.86 | 1.15 | 0.71 | 1.62 |  |
| ENSBTAG00000010161 | 0.113 |  | - |  | 1.78 | 1.07 | 0.71 | 1.66 |  |
| ENSBTAG00000020699 | 0.140 |  | - |  | 1.90 | 1.18 | 0.71 | 1.61 |  |
| ENSBTAG00000020350 | 0.173 |  | - |  | 1.50 | 0.79 | 0.72 | 1.90 |  |
| ENSBTAG00000039813 | 0.177 |  | - |  | 1.52 | 0.79 | 0.72 | 1.92 |  |
| ENSBTAG00000031458 | 0.110 |  | - |  | 1.91 | 1.19 | 0.73 | 1.61 |  |
| ENSBTAG00000003220 | 0.029 |  | - |  | 1.07 | 0.34 | 0.74 | 3.15 |  |
| ENSBTAG00000015551 | 0.195 |  | - |  | 1.36 | 0.62 | 0.75 | 2.19 |  |
| ENSBTAG00000012208 | 0.214 |  | - |  | 1.94 | 1.17 | 0.77 | 1.66 |  |
| ENSBTAG00000012186 | 0.045 |  | - |  | 1.52 | 0.73 | 0.79 | 2.08 |  |
| ENSBTAG00000003624 | 0.009 |  | - |  | 1.65 | 0.85 | 0.80 | 1.94 |  |
| ENSBTAG00000012809 | 0.160 |  | - |  | 1.86 | 1.04 | 0.82 | 1.79 |  |
| ENSBTAG00000001219 | 0.128 |  | - |  | 1.33 | 0.49 | 0.84 | 2.71 |  |
| ENSBTAG00000010458 | 0.184 |  | - |  | 2.54 | 1.67 | 0.87 | 1.52 |  |
| ENSBTAG00000015113 | 0.058 |  | - |  | 1.79 | 0.91 | 0.88 | 1.97 |  |
| ENSBTAG00000026917 | 0.495 |  | - |  | 2.36 | 1.48 | 0.88 | 1.59 |  |
| ENSBTAG00000009956 | 0.001 |  | - |  | 1.43 | 0.55 | 0.89 | 2.60 |  |
| ENSBTAG00000046922 | 0.148 |  | TF |  | 2.17 | 1.26 | 0.90 | 1.72 |  |
| ENSBTAG00000006730 | 0.171 |  | - |  | 2.10 | 1.19 | 0.91 | 1.76 |  |
| ENSBTAG00000047586 | 0.059 |  | - |  | 1.98 | 1.07 | 0.91 | 1.85 |  |
| ENSBTAG00000005269 | 0.105 |  | - |  | 2.03 | 1.11 | 0.92 | 1.83 |  |
| ENSBTAG00000014370 | 0.268 |  | - |  | 2.54 | 1.62 | 0.92 | 1.57 |  |
| ENSBTAG00000017896 | 0.166 |  | - |  | 2.08 | 1.15 | 0.93 | 1.81 |  |
| ENSBTAG00000046440 | 0.559 |  | - |  | 2.63 | 1.70 | 0.93 | 1.55 |  |
| ENSBTAG00000046308 | 0.517 |  | - |  | 2.65 | 1.70 | 0.95 | 1.56 |  |
| ENSBTAG00000043566 | 0.037 |  | - |  | 2.45 | 1.50 | 0.95 | 1.63 |  |
| ENSBTAG00000046583 | 0.114 |  | - |  | 2.44 | 1.48 | 0.96 | 1.65 |  |
| ENSBTAG00000046161 | 0.102 |  | - |  | 2.34 | 1.36 | 0.97 | 1.72 |  |
| ENSBTAG00000011693 | 0.147 |  | - |  | 2.36 | 1.39 | 0.97 | 1.70 |  |
| ENSBTAG00000030483 | 0.100 |  | - |  | 2.09 | 1.09 | 0.99 | 1.92 |  |
| ENSBTAG00000047947 | 0.523 |  | - |  | 2.37 | 1.37 | 1.00 | 1.73 |  |
| ENSBTAG00000047023 | 0.500 |  | - |  | 2.21 | 1.19 | 1.02 | 1.86 |  |
| ENSBTAG00000039462 | 0.139 |  | - |  | 1.66 | 0.64 | 1.02 | 2.59 |  |
| ENSBTAG00000038748 | 0.499 |  | - |  | 2.53 | 1.49 | 1.04 | 1.70 |  |
| ENSBTAG00000011666 | 0.136 |  | - |  | 2.47 | 1.43 | 1.04 | 1.73 |  |
| ENSBTAG00000018563 | 0.001 |  | - |  | 1.31 | 0.24 | 1.07 | 5.46 |  |
| ENSBTAG00000048020 | 0.466 |  | - |  | 2.48 | 1.40 | 1.08 | 1.77 |  |
| ENSBTAG00000044443 | 0.436 |  | - |  | 2.63 | 1.51 | 1.13 | 1.74 |  |
| ENSBTAG00000042499 | 0.437 |  | - |  | 2.23 | 1.06 | 1.18 | 2.10 |  |
| ENSBTAG00000047325 | 0.005 |  | - |  | 2.06 | 0.87 | 1.19 | 2.37 |  |
| ENSBTAG00000047529 | 0.327 |  | - |  | 2.51 | 1.31 | 1.20 | 1.92 |  |
| ENSBTAG00000004129 | 0.187 |  | - |  | 2.57 | 1.32 | 1.26 | 1.95 |  |
| ENSBTAG00000047277 | 0.416 |  | - |  | 1.99 | 0.73 | 1.26 | 2.73 |  |
| ENSBTAG00000000960 | 0.388 |  | - |  | 2.18 | 0.91 | 1.27 | 2.40 |  |
| ENSBTAG00000024991 | 0.413 |  | - |  | 1.86 | 0.57 | 1.29 | 3.26 |  |
| ENSBTAG00000006977 | 0.398 |  | - |  | 2.74 | 1.44 | 1.30 | 1.90 |  |
| ENSBTAG00000044236 | 0.403 |  | - |  | 2.00 | 0.69 | 1.31 | 2.90 |  |
| ENSBTAG00000025023 | 0.410 |  | - |  | 1.81 | 0.49 | 1.32 | 3.69 |  |
| ENSBTAG00000045916 | 0.393 |  | - |  | 2.14 | 0.79 | 1.34 | 2.71 |  |
| ENSBTAG00000043576 | 0.361 |  | - |  | 2.35 | 1.00 | 1.35 | 2.35 |  |
| ENSBTAG00000035064 | 0.378 |  | - |  | 2.29 | 0.93 | 1.36 | 2.46 |  |
| ENSBTAG00000011735 | 0.336 |  | - |  | 2.95 | 1.57 | 1.38 | 1.88 |  |
| ENSBTAG00000014239 | 0.309 |  | - |  | 3.00 | 1.61 | 1.39 | 1.86 |  |
| ENSBTAG00000012223 | 0.268 |  | - |  | 3.49 | 2.06 | 1.43 | 1.69 |  |
| ENSBTAG00000007642 | 0.233 |  | - |  | 4.21 | 2.77 | 1.43 | 1.52 |  |
| ENSBTAG00000008328 | 0.244 |  | - |  | 3.49 | 2.04 | 1.46 | 1.71 |  |
| ENSBTAG00000006152 | 0.499 |  | - |  | 3.94 | 2.48 | 1.46 | 1.59 |  |
| ENSBTAG00000035158 | 0.224 |  | - |  | 3.89 | 2.41 | 1.48 | 1.61 |  |
| ENSBTAG00000044132 | 0.295 |  | - |  | 3.19 | 1.71 | 1.49 | 1.87 |  |
| ENSBTAG00000012604 | 0.491 |  | - |  | 4.34 | 2.85 | 1.49 | 1.52 |  |
| ENSBTAG00000026497 | 0.328 |  | - |  | 2.16 | 0.67 | 1.49 | 3.22 |  |
| ENSBTAG00000022396 | 0.305 |  | - |  | 2.71 | 1.18 | 1.53 | 2.30 |  |
| ENSBTAG00000001063 | 0.221 |  | TF |  | 3.61 | 2.07 | 1.54 | 1.74 |  |
| ENSBTAG00000000892 | 0.184 |  | - |  | 4.25 | 2.71 | 1.54 | 1.57 |  |
| ENSBTAG00000010253 | 0.181 |  | - |  | 4.28 | 2.74 | 1.54 | 1.56 |  |
| ENSBTAG00000039012 | 0.191 |  | - |  | 4.16 | 2.59 | 1.56 | 1.61 |  |
| ENSBTAG00000001474 | 0.240 |  | - |  | 3.73 | 2.17 | 1.56 | 1.72 |  |
| ENSBTAG00000009976 | 0.297 |  | - |  | 2.44 | 0.87 | 1.57 | 2.80 |  |
| ENSBTAG00000004386 | 0.192 |  | - |  | 3.80 | 2.22 | 1.58 | 1.71 |  |
| ENSBTAG00000014113 | 0.251 |  | - |  | 3.38 | 1.77 | 1.61 | 1.91 |  |
| ENSBTAG00000004817 | 0.229 |  | - |  | 3.19 | 1.57 | 1.61 | 2.03 |  |
| ENSBTAG00000046232 | 0.258 |  | - |  | 2.79 | 1.18 | 1.62 | 2.36 |  |
| ENSBTAG00000015274 | 0.277 |  | - |  | 3.38 | 1.75 | 1.63 | 1.93 |  |
| ENSBTAG00000047591 | 0.231 |  | - |  | 3.46 | 1.82 | 1.63 | 1.90 |  |
| ENSBTAG00000012822 | 0.370 |  | - |  | 3.91 | 2.27 | 1.64 | 1.72 |  |
| ENSBTAG00000001341 | 0.181 |  | - |  | 3.87 | 2.23 | 1.64 | 1.74 |  |
| ENSBTAG00000043572 | 0.252 |  | - |  | 2.40 | 0.69 | 1.71 | 3.48 |  |
| ENSBTAG00000038368 | 0.268 |  | - |  | 2.21 | 0.50 | 1.71 | 4.42 |  |
| ENSBTAG00000021360 | 0.124 |  | - |  | 4.20 | 2.48 | 1.72 | 1.69 |  |
| ENSBTAG00000046901 | 0.110 |  | - |  | 4.57 | 2.78 | 1.79 | 1.64 |  |
| ENSBTAG00000042339 | 0.227 |  | - |  | 2.44 | 0.64 | 1.80 | 3.81 |  |
| ENSBTAG00000003691 | 0.147 |  | - |  | 3.98 | 2.18 | 1.80 | 1.83 |  |
| ENSBTAG00000037595 | 0.335 |  | - |  | 3.64 | 1.80 | 1.84 | 2.02 |  |
| ENSBTAG00000014514 | 0.124 |  | - |  | 4.24 | 2.39 | 1.85 | 1.77 |  |
| ENSBTAG00000047926 | 0.215 |  | - |  | 2.73 | 0.87 | 1.85 | 3.14 |  |
| ENSBTAG00000047548 | 0.269 |  | - |  | 4.47 | 2.57 | 1.90 | 1.74 |  |
| ENSBTAG00000030208 | 0.170 |  | - |  | 2.99 | 1.03 | 1.95 | 2.90 |  |
| ENSBTAG00000026088 | 0.296 |  | - |  | 4.02 | 2.04 | 1.97 | 1.97 |  |
| ENSBTAG00000011881 | 0.142 |  | - |  | 3.98 | 2.00 | 1.98 | 1.99 |  |
| ENSBTAG00000026417 | 0.257 |  | - |  | 4.40 | 2.42 | 1.98 | 1.82 |  |
| ENSBTAG00000002488 | 0.187 |  | - |  | 2.85 | 0.87 | 1.98 | 3.28 |  |
| ENSBTAG00000047508 | 0.354 |  | TF |  | 4.57 | 2.58 | 2.00 | 1.77 |  |
| ENSBTAG00000012319 | 0.166 |  | - |  | 3.09 | 1.05 | 2.04 | 2.94 |  |
| ENSBTAG00000012876 | 0.170 |  | - |  | 4.64 | 2.57 | 2.07 | 1.81 |  |
| ENSBTAG00000043258 | 0.290 |  | - |  | 3.40 | 1.33 | 2.08 | 2.56 |  |
| ENSBTAG00000005305 | 0.146 |  | - |  | 3.16 | 1.07 | 2.09 | 2.95 |  |
| ENSBTAG00000048167 | 0.376 |  | - |  | 4.82 | 2.68 | 2.14 | 1.80 |  |
| ENSBTAG00000039890 | 0.193 |  | - |  | 4.31 | 2.15 | 2.15 | 2.00 |  |
| ENSBTAG00000048269 | 0.328 |  | - |  | 6.77 | 4.58 | 2.20 | 1.48 |  |
| ENSBTAG00000031866 | 0.204 |  | - |  | 7.65 | 5.45 | 2.20 | 1.40 |  |
| ENSBTAG00000005235 | 0.024 |  | - |  | 8.65 | 6.45 | 2.21 | 1.34 |  |
| ENSBTAG00000047468 | 0.251 |  | - |  | 5.78 | 3.56 | 2.22 | 1.62 |  |
| ENSBTAG00000036659 | 0.256 |  | - |  | 3.54 | 1.32 | 2.22 | 2.68 |  |
| ENSBTAG00000013250 | 0.174 |  | - |  | 7.17 | 4.94 | 2.23 | 1.45 |  |
| ENSBTAG00000042989 | 0.405 |  | - |  | 5.91 | 3.66 | 2.25 | 1.61 |  |
| ENSBTAG00000042280 | 0.377 |  | - |  | 4.64 | 2.40 | 2.25 | 1.93 |  |
| ENSBTAG00000015632 | 0.190 |  | - |  | 4.20 | 1.94 | 2.26 | 2.16 |  |
| ENSBTAG00000031468 | 0.194 |  | - |  | 3.86 | 1.56 | 2.31 | 2.47 |  |
| ENSBTAG00000007956 | 0.141 |  | - |  | 4.71 | 2.40 | 2.31 | 1.96 |  |
| ENSBTAG00000048293 | 0.353 |  | - |  | 6.30 | 3.98 | 2.32 | 1.58 |  |
| ENSBTAG00000010032 | 0.125 |  | - |  | 6.04 | 3.71 | 2.33 | 1.63 |  |
| ENSBTAG00000009235 | 0.173 |  | - |  | 4.19 | 1.86 | 2.33 | 2.25 |  |
| ENSBTAG00000009599 | 0.191 |  | - |  | 4.06 | 1.68 | 2.38 | 2.42 |  |
| ENSBTAG00000009354 | 0.114 |  | - |  | 5.04 | 2.66 | 2.38 | 1.89 |  |
| ENSBTAG00000026495 | 0.229 |  | - |  | 3.38 | 0.99 | 2.39 | 3.41 |  |
| ENSBTAG00000021667 | 0.104 |  | - |  | 9.13 | 6.73 | 2.40 | 1.36 |  |
| ENSBTAG00000027172 | 0.130 |  | - |  | 4.76 | 2.34 | 2.42 | 2.03 |  |
| ENSBTAG00000002719 | 0.031 |  | - |  | 6.25 | 3.83 | 2.42 | 1.63 |  |
| ENSBTAG00000011420 | 0.083 |  | - |  | 5.41 | 2.98 | 2.44 | 1.82 |  |
| ENSBTAG00000046598 | 0.157 |  | - |  | 7.10 | 4.66 | 2.44 | 1.52 |  |
| ENSBTAG00000038532 | 0.256 |  | - |  | 5.50 | 3.05 | 2.45 | 1.80 |  |
| ENSBTAG00000006990 | 0.020 |  | TF |  | 7.08 | 4.62 | 2.46 | 1.53 |  |
| ENSBTAG00000010828 | 0.125 |  | - |  | 4.91 | 2.43 | 2.48 | 2.02 |  |
| ENSBTAG00000043530 | 0.213 |  | - |  | 3.24 | 0.73 | 2.51 | 4.44 |  |
| ENSBTAG00000003409 | 0.147 |  | - |  | 7.07 | 4.50 | 2.57 | 1.57 |  |
| ENSBTAG00000037578 | 0.179 |  | - |  | 4.16 | 1.59 | 2.57 | 2.62 |  |
| ENSBTAG00000019490 | 0.159 |  | - |  | 3.86 | 1.27 | 2.59 | 3.04 |  |
| ENSBTAG00000011700 | 0.130 |  | - |  | 4.40 | 1.76 | 2.64 | 2.50 |  |
| ENSBTAG00000021161 | 0.060 |  | - |  | 5.25 | 2.60 | 2.65 | 2.02 |  |
| ENSBTAG00000003398 | 0.043 |  | - |  | 5.52 | 2.86 | 2.66 | 1.93 |  |
| ENSBTAG00000046813 | 0.159 |  | - |  | 5.49 | 2.83 | 2.67 | 1.94 |  |
| ENSBTAG00000046076 | 0.080 |  | - |  | 3.95 | 1.28 | 2.68 | 3.09 |  |
| ENSBTAG00000037902 | 0.078 |  | - |  | 5.11 | 2.41 | 2.69 | 2.12 |  |
| ENSBTAG00000048185 | 0.210 |  | - |  | 7.37 | 4.62 | 2.75 | 1.60 |  |
| ENSBTAG00000001477 | 0.172 |  | - |  | 4.95 | 2.19 | 2.76 | 2.26 |  |
| ENSBTAG00000040347 | 0.152 |  | - |  | 6.53 | 3.77 | 2.76 | 1.73 |  |
| ENSBTAG00000042471 | 0.162 |  | - |  | 3.40 | 0.62 | 2.79 | 5.48 |  |
| ENSBTAG00000047250 | 0.235 |  | - |  | 5.18 | 2.37 | 2.80 | 2.19 |  |
| ENSBTAG00000034681 | 0.024 |  | - |  | 8.88 | 6.07 | 2.81 | 1.46 |  |
| ENSBTAG00000045792 | 0.162 |  | - |  | 3.36 | 0.54 | 2.82 | 6.22 |  |
| ENSBTAG00000001476 | 0.138 |  | - |  | 4.00 | 1.18 | 2.82 | 3.39 |  |
| ENSBTAG00000018077 | 0.049 |  | - |  | 7.95 | 5.03 | 2.92 | 1.58 |  |
| ENSBTAG00000044113 | 0.106 |  | - |  | 5.26 | 2.32 | 2.94 | 2.27 |  |
| ENSBTAG00000038843 | 0.109 |  | - |  | 6.75 | 3.74 | 3.01 | 1.80 |  |
| ENSBTAG00000031845 | 0.026 |  | - |  | 8.67 | 5.59 | 3.08 | 1.55 |  |
| ENSBTAG00000047113 | 0.051 |  | - |  | 5.85 | 2.74 | 3.11 | 2.14 |  |
| ENSBTAG00000003352 | 0.128 |  | - |  | 4.83 | 1.71 | 3.11 | 2.82 |  |
| ENSBTAG00000045973 | 0.140 |  | - |  | 7.53 | 4.40 | 3.13 | 1.71 |  |
| ENSBTAG00000002674 | 0.108 |  | - |  | 6.69 | 3.53 | 3.17 | 1.90 |  |
| ENSBTAG00000034337 | 0.188 |  | - |  | 6.06 | 2.89 | 3.17 | 2.10 |  |
| ENSBTAG00000046183 | 0.198 |  | - |  | 5.84 | 2.56 | 3.27 | 2.28 |  |
| ENSBTAG00000002009 | 0.104 |  | - |  | 8.51 | 5.18 | 3.33 | 1.64 |  |
| ENSBTAG00000043589 | 0.072 |  | - |  | 3.91 | 0.56 | 3.35 | 6.98 |  |
| ENSBTAG00000042679 | 0.159 |  | - |  | 7.19 | 3.77 | 3.42 | 1.91 |  |
| ENSBTAG00000038706 | 0.074 |  | - |  | 5.19 | 1.57 | 3.62 | 3.31 |  |
| ENSBTAG00000043268 | 0.161 |  | - |  | 5.97 | 2.34 | 3.63 | 2.55 |  |
| ENSBTAG00000046420 | 0.154 |  | - |  | 5.98 | 2.34 | 3.63 | 2.56 |  |
| ENSBTAG00000048156 | 0.025 |  | - |  | 8.61 | 4.26 | 4.35 | 2.02 |  |
| ENSBTAG00000043695 | 0.024 |  | - |  | 8.59 | 3.88 | 4.70 | 2.21 |  |
| ENSBTAG00000045039 | 0.033 |  | - |  | 7.40 | 2.55 | 4.85 | 2.90 |  |
| ENSBTAG00000043574 | 0.007 |  | - |  | 7.19 | 1.91 | 5.28 | 3.76 |  |

^†^ ENSB Tag: Ensembl gene identifier according to <http://www.ensembl.org/>.

^‡^ TF represents transcription factor identified with reference to the Animal TFDB classification: “TF” indicates that the gene is a transcription factor and “-“ represents otherwise.

^§^ PRE and POST are the average gene expression values reported as normalized base-2 log transformed RPKM values for pre- and post-pubertal heifers.

^¶^ FC (fold change) describes the ratio of expression between pre- and post-puberty; FC = POST/PRE. FC between 0 and 0.5 means post-pubertal expression has decreased more than 2 folds in comparison to pre-pubertal expression, FC between 0.5 to 1 means no difference in expression in both pubertal stages while FC of more than 1 indicates increase in expression at post-pubertal.

Table S2 A total of 1,452 transcription factors (TF) were identified in muscle of Bos indicus Brahman heifers, with reference to Animal TFDB3.0 database (<http://bioinfo.life.hust.edu.cn/AnimalTFDB/#!/>).

| ENSB Tag | Gene |
| --- | --- |
| ENSBTAG00000000011 | *ENSBTAG00000000011* |
| ENSBTAG00000000061 | *PCDH7* |
| ENSBTAG00000000070 | *F13B* |
| ENSBTAG00000000085 | *CYP1A2* |
| ENSBTAG00000000123 | *HAO2* |
| ENSBTAG00000000128 | *FGF18* |
| ENSBTAG00000000229 | *ENSBTAG00000000229* |
| ENSBTAG00000000259 | *CHIA* |
| ENSBTAG00000000396 | *PIM1* |
| ENSBTAG00000000533 | *ZNF684* |
| ENSBTAG00000000644 | *S100A5* |
| ENSBTAG00000000835 | *BCAS1* |
| ENSBTAG00000000859 | *SLC38A1* |
| ENSBTAG00000000892 | *CGAS* |
| ENSBTAG00000000960 | *SPATA25* |
| ENSBTAG00000001063 | *HOXA4* |
| ENSBTAG00000001079 | *ENSBTAG00000001079* |
| ENSBTAG00000001139 | *ACHE* |
| ENSBTAG00000001152 | *TLE6* |
| ENSBTAG00000001219 | *ENSBTAG00000001219* |
| ENSBTAG00000001341 | *ENSBTAG00000001341* |
| ENSBTAG00000001346 | *STRA8* |
| ENSBTAG00000001392 | *RDH16* |
| ENSBTAG00000001474 | *NRGN* |
| ENSBTAG00000001476 | *ENSBTAG00000001476* |
| ENSBTAG00000001477 | *ENSBTAG00000001477* |
| ENSBTAG00000001517 | *KRT18* |
| ENSBTAG00000001658 | *AKR1D1* |
| ENSBTAG00000001836 | *ENSBTAG00000001836* |
| ENSBTAG00000001852 | *BREH1* |
| ENSBTAG00000001886 | *FAM122B* |
| ENSBTAG00000001981 | *SLC6A17* |
| ENSBTAG00000002009 | *ENSBTAG00000002009* |
| ENSBTAG00000002255 | *BHMT* |
| ENSBTAG00000002488 | *LDHC* |
| ENSBTAG00000002642 | *SLC17A2* |
| ENSBTAG00000002674 | *GNGT1* |
| ENSBTAG00000002719 | *ORC1* |
| ENSBTAG00000002799 | *SLC39A5* |
| ENSBTAG00000002878 | *CHRD* |
| ENSBTAG00000002914 | *GALNT18* |
| ENSBTAG00000002963 | *SAA4* |
| ENSBTAG00000003200 | *ENSBTAG00000003200* |
| ENSBTAG00000003212 | *NNAT* |
| ENSBTAG00000003220 | *ENSBTAG00000003220* |
| ENSBTAG00000003236 | *ENSBTAG00000003236* |
| ENSBTAG00000003253 | *NPPC* |
| ENSBTAG00000003352 | *ENSBTAG00000003352* |
| ENSBTAG00000003398 | *KCNG1* |
| ENSBTAG00000003409 | *FKBP1B* |
| ENSBTAG00000003515 | *SNCG* |
| ENSBTAG00000003624 | *CNTF* |
| ENSBTAG00000003691 | *NTSR2* |
| ENSBTAG00000003774 | *C8A* |
| ENSBTAG00000003894 | *NDRG4* |
| ENSBTAG00000003898 | *HMGCS2* |
| ENSBTAG00000004003 | *ENSBTAG00000004003* |
| ENSBTAG00000004129 | *CCL11* |
| ENSBTAG00000004170 | *ENSBTAG00000004170* |
| ENSBTAG00000004224 | *C7H19ORF38* |
| ENSBTAG00000004303 | *SLC27A2* |
| ENSBTAG00000004384 | *APOF* |
| ENSBTAG00000004386 | *SOCS1* |
| ENSBTAG00000004817 | *ENSBTAG00000004817* |
| ENSBTAG00000005033 | *FAM57B* |
| ENSBTAG00000005099 | *ENSBTAG00000005099* |
| ENSBTAG00000005235 | *DPP10* |
| ENSBTAG00000005244 | *RASL11A* |
| ENSBTAG00000005251 | *ENSBTAG00000005251* |
| ENSBTAG00000005269 | *CCNB2* |
| ENSBTAG00000005305 | *NTS* |
| ENSBTAG00000005382 | *ENSBTAG00000005382* |
| ENSBTAG00000005479 | *LIPC* |
| ENSBTAG00000005596 | *IGFBP2* |
| ENSBTAG00000005647 | *GPR132* |
| ENSBTAG00000005722 | *ENSBTAG00000005722* |
| ENSBTAG00000006152 | *ENSBTAG00000006152* |
| ENSBTAG00000006231 | *ACSBG1* |
| ENSBTAG00000006424 | *ENSBTAG00000006424* |
| ENSBTAG00000006606 | *RFLNB* |
| ENSBTAG00000006730 | *SUSD2* |
| ENSBTAG00000006977 | *PLP1* |
| ENSBTAG00000006990 | *MYRF* |
| ENSBTAG00000007043 | *ENSBTAG00000007043* |
| ENSBTAG00000007049 | *MBL2* |
| ENSBTAG00000007402 | *ZFAND2A* |
| ENSBTAG00000007507 | *FAAH* |
| ENSBTAG00000007514 | *CPNE5* |
| ENSBTAG00000007642 | *ENSBTAG00000007642* |
| ENSBTAG00000007720 | *ENSBTAG00000007720* |
| ENSBTAG00000007816 | *ENSBTAG00000007816* |
| ENSBTAG00000007834 | *PPP1R16A* |
| ENSBTAG00000007881 | *IFIT1* |
| ENSBTAG00000007956 | *FRAT1* |
| ENSBTAG00000008026 | *OXT* |
| ENSBTAG00000008127 | *RBP5* |
| ENSBTAG00000008222 | *EEF2KMT* |
| ENSBTAG00000008238 | *S100A7* |
| ENSBTAG00000008259 | *ACOD1* |
| ENSBTAG00000008323 | *SNAP25* |
| ENSBTAG00000008328 | *ENSBTAG00000008328* |
| ENSBTAG00000008384 | *SLC25A47* |
| ENSBTAG00000008441 | *SOCS3* |
| ENSBTAG00000008551 | *TUSC1* |
| ENSBTAG00000008711 | *BOK* |
| ENSBTAG00000008726 | *NCS1* |
| ENSBTAG00000008900 | *CELA1* |
| ENSBTAG00000008913 | *TMEM98* |
| ENSBTAG00000009002 | *ENSBTAG00000009002* |
| ENSBTAG00000009235 | *KCNMB3* |
| ENSBTAG00000009350 | *PLA2G12B* |
| ENSBTAG00000009354 | *EVI2A* |
| ENSBTAG00000009493 | *BCL3* |
| ENSBTAG00000009501 | *KLKB1* |
| ENSBTAG00000009573 | *C15H11ORF49* |
| ENSBTAG00000009599 | *ENSBTAG00000009599* |
| ENSBTAG00000009735 | *A1BG* |
| ENSBTAG00000009956 | *CENPM* |
| ENSBTAG00000009976 | *ENSBTAG00000009976* |
| ENSBTAG00000010032 | *NTM* |
| ENSBTAG00000010161 | *CCL21* |
| ENSBTAG00000010177 | *GOLT1A* |
| ENSBTAG00000010236 | *CIDEB* |
| ENSBTAG00000010253 | *SYTL1* |
| ENSBTAG00000010458 | *ENSBTAG00000010458* |
| ENSBTAG00000010531 | *CYP1B1* |
| ENSBTAG00000010641 | *APH1B* |
| ENSBTAG00000010828 | *ENSBTAG00000010828* |
| ENSBTAG00000011420 | *CA9* |
| ENSBTAG00000011655 | *SAMD10* |
| ENSBTAG00000011666 | *THRSP* |
| ENSBTAG00000011693 | *LENG9* |
| ENSBTAG00000011700 | *PLLP* |
| ENSBTAG00000011735 | *ENSBTAG00000011735* |
| ENSBTAG00000011881 | *TDRD10* |
| ENSBTAG00000012164 | *CP* |
| ENSBTAG00000012184 | *ENSBTAG00000012184* |
| ENSBTAG00000012186 | *DKKL1* |
| ENSBTAG00000012208 | *ENSBTAG00000012208* |
| ENSBTAG00000012223 | *TNFSF14* |
| ENSBTAG00000012319 | *KCNE5* |
| ENSBTAG00000012540 | *ENSBTAG00000012540* |
| ENSBTAG00000012604 | *NCMAP* |
| ENSBTAG00000012640 | *S100A8* |
| ENSBTAG00000012692 | *ENSBTAG00000012692* |
| ENSBTAG00000012809 | *PHF24* |
| ENSBTAG00000012822 | *MGC137036* |
| ENSBTAG00000012876 | *ENSBTAG00000012876* |
| ENSBTAG00000013020 | *ENSBTAG00000013020* |
| ENSBTAG00000013131 | *FAM110D* |
| ENSBTAG00000013134 | *ENSBTAG00000013134* |
| ENSBTAG00000013250 | *PCSK1N* |
| ENSBTAG00000013294 | *DYRK3* |
| ENSBTAG00000013333 | *GYS2* |
| ENSBTAG00000013429 | *CLEC4G* |
| ENSBTAG00000013598 | *RSPO2* |
| ENSBTAG00000013736 | *PROM1* |
| ENSBTAG00000013907 | *CRP* |
| ENSBTAG00000013973 | *SERPIND1* |
| ENSBTAG00000014113 | *CCL8* |
| ENSBTAG00000014135 | *WDR17* |
| ENSBTAG00000014217 | *HHEX* |
| ENSBTAG00000014239 | *CCNB1* |
| ENSBTAG00000014250 | *ZC3HAV1L* |
| ENSBTAG00000014252 | *SPTLC3* |
| ENSBTAG00000014370 | *NETO2* |
| ENSBTAG00000014514 | *SEC1* |
| ENSBTAG00000014517 | *KLB* |
| ENSBTAG00000014705 | *HES4* |
| ENSBTAG00000014899 | *ENSBTAG00000014899* |
| ENSBTAG00000015047 | *ENSBTAG00000015047* |
| ENSBTAG00000015113 | *CITED4* |
| ENSBTAG00000015164 | *SLC27A5* |
| ENSBTAG00000015274 | *PRL* |
| ENSBTAG00000015347 | *WNT10B* |
| ENSBTAG00000015387 | *RAB40B* |
| ENSBTAG00000015402 | *GREB1* |
| ENSBTAG00000015551 | *ENSBTAG00000015551* |
| ENSBTAG00000015632 | *TNFRSF18* |
| ENSBTAG00000015836 | *ENSBTAG00000015836* |
| ENSBTAG00000015880 | *PRIMA1* |
| ENSBTAG00000016391 | *ENSBTAG00000016391* |
| ENSBTAG00000016430 | *METTL8* |
| ENSBTAG00000016494 | *RELT* |
| ENSBTAG00000016987 | *WDCP* |
| ENSBTAG00000017020 | *S100G* |
| ENSBTAG00000017155 | *TRIM32* |
| ENSBTAG00000017242 | *FADS6* |
| ENSBTAG00000017333 | *PRX* |
| ENSBTAG00000017335 | *HGFAC* |
| ENSBTAG00000017343 | *ENSBTAG00000017343* |
| ENSBTAG00000017405 | *RORC* |
| ENSBTAG00000017529 | *CA8* |
| ENSBTAG00000017670 | *ENSBTAG00000017670* |
| ENSBTAG00000017714 | *C4BPB* |
| ENSBTAG00000017896 | *ENSBTAG00000017896* |
| ENSBTAG00000018069 | *TNFSF15* |
| ENSBTAG00000018077 | *LYPD3* |
| ENSBTAG00000018161 | *TBX18* |
| ENSBTAG00000018481 | *ENSBTAG00000018481* |
| ENSBTAG00000018563 | *SFRP2* |
| ENSBTAG00000018634 | *ENSBTAG00000018634* |
| ENSBTAG00000018806 | *PSRC1* |
| ENSBTAG00000018869 | *IGSF6* |
| ENSBTAG00000018926 | *LEAP2* |
| ENSBTAG00000019490 | *ENSBTAG00000019490* |
| ENSBTAG00000019533 | *ADTRP* |
| ENSBTAG00000019616 | *APCS* |
| ENSBTAG00000019764 | *APOA5* |
| ENSBTAG00000019993 | *APLN* |
| ENSBTAG00000020049 | *CABP1* |
| ENSBTAG00000020076 | *ENSBTAG00000020076* |
| ENSBTAG00000020099 | *STMN2* |
| ENSBTAG00000020209 | *ENSBTAG00000020209* |
| ENSBTAG00000020350 | *DUSP2* |
| ENSBTAG00000020512 | *GJB1* |
| ENSBTAG00000020558 | *APOC2* |
| ENSBTAG00000020597 | *FMO3* |
| ENSBTAG00000020685 | *ENSBTAG00000020685* |
| ENSBTAG00000020699 | *TSPAN11* |
| ENSBTAG00000020790 | *ENSBTAG00000020790* |
| ENSBTAG00000020803 | *ENSBTAG00000020803* |
| ENSBTAG00000021161 | *CREG2* |
| ENSBTAG00000021219 | *Sep-01* |
| ENSBTAG00000021360 | *ENSBTAG00000021360* |
| ENSBTAG00000021437 | *GPR174* |
| ENSBTAG00000021649 | *RPL27A* |
| ENSBTAG00000021667 | *ENSBTAG00000021667* |
| ENSBTAG00000021887 | *DPYS* |
| ENSBTAG00000022246 | *C29H11ORF86* |
| ENSBTAG00000022329 | *SLCO1B3* |
| ENSBTAG00000022372 | *ENSBTAG00000022372* |
| ENSBTAG00000022394 | *ENSBTAG00000022394* |
| ENSBTAG00000022396 | *ENSBTAG00000022396* |
| ENSBTAG00000022471 | *TTPA* |
| ENSBTAG00000023007 | *ENSBTAG00000023007* |
| ENSBTAG00000023032 | *SFTPA1* |
| ENSBTAG00000023411 | *ENSBTAG00000023411* |
| ENSBTAG00000024272 | *ENSBTAG00000024272* |
| ENSBTAG00000024490 | *ENSBTAG00000024490* |
| ENSBTAG00000024545 | *ENSBTAG00000024545* |
| ENSBTAG00000024675 | *CYSLTR1* |
| ENSBTAG00000024851 | *TRIM14* |
| ENSBTAG00000024874 | *ENSBTAG00000024874* |
| ENSBTAG00000024983 | *FBXO47* |
| ENSBTAG00000024991 | *MKRN2OS* |
| ENSBTAG00000025023 | *ENSBTAG00000025023* |
| ENSBTAG00000025485 | *ENSBTAG00000025485* |
| ENSBTAG00000026070 | *ENSBTAG00000026070* |
| ENSBTAG00000026088 | *LYZ2* |
| ENSBTAG00000026417 | *ENSBTAG00000026417* |
| ENSBTAG00000026495 | *ENSBTAG00000026495* |
| ENSBTAG00000026497 | *A4GALT* |
| ENSBTAG00000026848 | *ENSBTAG00000026848* |
| ENSBTAG00000026882 | *ENSBTAG00000026882* |
| ENSBTAG00000026917 | *ENSBTAG00000026917* |
| ENSBTAG00000027172 | *ENSBTAG00000027172* |
| ENSBTAG00000027610 | *ENSBTAG00000027610* |
| ENSBTAG00000029828 | *ENSBTAG00000029828* |
| ENSBTAG00000030208 | *ERFE* |
| ENSBTAG00000030483 | *KLK7* |
| ENSBTAG00000030540 | *ZDHHC12* |
| ENSBTAG00000031388 | *DNAAF4* |
| ENSBTAG00000031458 | *ENSBTAG00000031458* |
| ENSBTAG00000031468 | *ENSBTAG00000031468* |
| ENSBTAG00000031600 | *ENSBTAG00000031600* |
| ENSBTAG00000031647 | *LRG1* |
| ENSBTAG00000031774 | *HIST1H1E* |
| ENSBTAG00000031845 | *ENSBTAG00000031845* |
| ENSBTAG00000031866 | *ENSBTAG00000031866* |
| ENSBTAG00000032017 | *ENSBTAG00000032017* |
| ENSBTAG00000032151 | *ENSBTAG00000032151* |
| ENSBTAG00000032198 | *ENSBTAG00000032198* |
| ENSBTAG00000032217 | *ENSBTAG00000032217* |
| ENSBTAG00000032350 | *ENSBTAG00000032350* |
| ENSBTAG00000033225 | *CNTN4* |
| ENSBTAG00000033365 | *METTL24* |
| ENSBTAG00000033740 | *ENSBTAG00000033740* |
| ENSBTAG00000034337 | *ENSBTAG00000034337* |
| ENSBTAG00000034681 | *CA10* |
| ENSBTAG00000035064 | *C4H7ORF57* |
| ENSBTAG00000035129 | *ENSBTAG00000035129* |
| ENSBTAG00000035158 | *TMEM249* |
| ENSBTAG00000035572 | *ENSBTAG00000035572* |
| ENSBTAG00000035654 | *ENSBTAG00000035654* |
| ENSBTAG00000035975 | *ENSBTAG00000035975* |
| ENSBTAG00000036061 | *ENSBTAG00000036061* |
| ENSBTAG00000036111 | *ENSBTAG00000036111* |
| ENSBTAG00000036659 | *ENSBTAG00000036659* |
| ENSBTAG00000037452 | *ENSBTAG00000037452* |
| ENSBTAG00000037578 | *ENSBTAG00000037578* |
| ENSBTAG00000037595 | *ENSBTAG00000037595* |
| ENSBTAG00000037634 | *ENSBTAG00000037634* |
| ENSBTAG00000037856 | *ENSBTAG00000037856* |
| ENSBTAG00000037890 | *CYP4A11* |
| ENSBTAG00000037902 | *FAM229A* |
| ENSBTAG00000038073 | *ENSBTAG00000038073* |
| ENSBTAG00000038171 | *CFHR5* |
| ENSBTAG00000038361 | *SERPINA11* |
| ENSBTAG00000038368 | *ENSBTAG00000038368* |
| ENSBTAG00000038532 | *ENSBTAG00000038532* |
| ENSBTAG00000038698 | *ENSBTAG00000038698* |
| ENSBTAG00000038706 | *MT1E* |
| ENSBTAG00000038710 | *ENSBTAG00000038710* |
| ENSBTAG00000038748 | *HBG* |
| ENSBTAG00000038843 | *NKG2C* |
| ENSBTAG00000038888 | *ZBTB9* |
| ENSBTAG00000039012 | *MFSD6L* |
| ENSBTAG00000039462 | *PCLAF* |
| ENSBTAG00000039477 | *TPBG* |
| ENSBTAG00000039550 | *ENSBTAG00000039550* |
| ENSBTAG00000039813 | *ENSBTAG00000039813* |
| ENSBTAG00000039890 | *RTL8C* |
| ENSBTAG00000040070 | *SCGB1D* |
| ENSBTAG00000040167 | *ENSBTAG00000040167* |
| ENSBTAG00000040337 | *ENSBTAG00000040337* |
| ENSBTAG00000040347 | *GPC6* |
| ENSBTAG00000042180 | *ENSBTAG00000042180* |
| ENSBTAG00000042217 | *ENSBTAG00000042217* |
| ENSBTAG00000042234 | *ENSBTAG00000042234* |
| ENSBTAG00000042280 | *ENSBTAG00000042280* |
| ENSBTAG00000042339 | *ENSBTAG00000042339* |
| ENSBTAG00000042447 | *ENSBTAG00000042447* |
| ENSBTAG00000042471 | *ENSBTAG00000042471* |
| ENSBTAG00000042499 | *ENSBTAG00000042499* |
| ENSBTAG00000042504 | *ENSBTAG00000042504* |
| ENSBTAG00000042678 | *ENSBTAG00000042678* |
| ENSBTAG00000042679 | *ENSBTAG00000042679* |
| ENSBTAG00000042723 | *ENSBTAG00000042723* |
| ENSBTAG00000042758 | *ENSBTAG00000042758* |
| ENSBTAG00000042950 | *ENSBTAG00000042950* |
| ENSBTAG00000042963 | *ENSBTAG00000042963* |
| ENSBTAG00000042974 | *ENSBTAG00000042974* |
| ENSBTAG00000042989 | *ENSBTAG00000042989* |
| ENSBTAG00000043000 | *ENSBTAG00000043000* |
| ENSBTAG00000043086 | *ENSBTAG00000043086* |
| ENSBTAG00000043258 | *ENSBTAG00000043258* |
| ENSBTAG00000043268 | *ENSBTAG00000043268* |
| ENSBTAG00000043378 | *ENSBTAG00000043378* |
| ENSBTAG00000043400 | *ENSBTAG00000043400* |
| ENSBTAG00000043519 | *ENSBTAG00000043519* |
| ENSBTAG00000043530 | *ENSBTAG00000043530* |
| ENSBTAG00000043566 | *ENSBTAG00000043566* |
| ENSBTAG00000043572 | *ENSBTAG00000043572* |
| ENSBTAG00000043574 | *ENSBTAG00000043574* |
| ENSBTAG00000043576 | *ENSBTAG00000043576* |
| ENSBTAG00000043580 | *ENSBTAG00000043580* |
| ENSBTAG00000043589 | *ENSBTAG00000043589* |
| ENSBTAG00000043695 | *ENSBTAG00000043695* |
| ENSBTAG00000043969 | *CALN1* |
| ENSBTAG00000044113 | *ENSBTAG00000044113* |
| ENSBTAG00000044132 | *NTN5* |
| ENSBTAG00000044236 | *ENSBTAG00000044236* |
| ENSBTAG00000044443 | *ENSBTAG00000044443* |
| ENSBTAG00000044712 | *ENSBTAG00000044712* |
| ENSBTAG00000044778 | *ENSBTAG00000044778* |
| ENSBTAG00000044962 | *ENSBTAG00000044962* |
| ENSBTAG00000045039 | *ENSBTAG00000045039* |
| ENSBTAG00000045080 | *ENSBTAG00000045080* |
| ENSBTAG00000045271 | *ENSBTAG00000045271* |
| ENSBTAG00000045327 | *ENSBTAG00000045327* |
| ENSBTAG00000045753 | *ENSBTAG00000045753* |
| ENSBTAG00000045792 | *ENSBTAG00000045792* |
| ENSBTAG00000045836 | *ENSBTAG00000045836* |
| ENSBTAG00000045916 | *ENSBTAG00000045916* |
| ENSBTAG00000045948 | *CSF2RA* |
| ENSBTAG00000045955 | *TSSK1B* |
| ENSBTAG00000045967 | *ENSBTAG00000045967* |
| ENSBTAG00000045973 | *ENSBTAG00000045973* |
| ENSBTAG00000046062 | *ENSBTAG00000046062* |
| ENSBTAG00000046076 | *ENSBTAG00000046076* |
| ENSBTAG00000046161 | *ENSBTAG00000046161* |
| ENSBTAG00000046183 | *ENSBTAG00000046183* |
| ENSBTAG00000046232 | *ENSBTAG00000046232* |
| ENSBTAG00000046277 | *RGS4* |
| ENSBTAG00000046308 | *ENSBTAG00000046308* |
| ENSBTAG00000046386 | *ENSBTAG00000046386* |
| ENSBTAG00000046420 | *ENSBTAG00000046420* |
| ENSBTAG00000046440 | *TRAM1L1* |
| ENSBTAG00000046506 | *ENSBTAG00000046506* |
| ENSBTAG00000046521 | *ENSBTAG00000046521* |
| ENSBTAG00000046544 | *SLC7A10* |
| ENSBTAG00000046583 | *TMEM61* |
| ENSBTAG00000046598 | *ENSBTAG00000046598* |
| ENSBTAG00000046628 | *LYZ3* |
| ENSBTAG00000046666 | *TTC9B* |
| ENSBTAG00000046744 | *PALM3* |
| ENSBTAG00000046813 | *ENSBTAG00000046813* |
| ENSBTAG00000046885 | *ENSBTAG00000046885* |
| ENSBTAG00000046901 | *ENSBTAG00000046901* |
| ENSBTAG00000046922 | *TWIST1* |
| ENSBTAG00000047023 | *ENSBTAG00000047023* |
| ENSBTAG00000047030 | *ENSBTAG00000047030* |
| ENSBTAG00000047040 | *SERPINA3-3* |
| ENSBTAG00000047113 | *ENSBTAG00000047113* |
| ENSBTAG00000047240 | *ENSBTAG00000047240* |
| ENSBTAG00000047250 | *ENSBTAG00000047250* |
| ENSBTAG00000047277 | *ENSBTAG00000047277* |
| ENSBTAG00000047302 | *ENSBTAG00000047302* |
| ENSBTAG00000047317 | *ENSBTAG00000047317* |
| ENSBTAG00000047325 | *GNGT2* |
| ENSBTAG00000047342 | *ENSBTAG00000047342* |
| ENSBTAG00000047357 | *RNF208* |
| ENSBTAG00000047361 | *ENSBTAG00000047361* |
| ENSBTAG00000047431 | *ENSBTAG00000047431* |
| ENSBTAG00000047449 | *NK2B* |
| ENSBTAG00000047468 | *FAM78B* |
| ENSBTAG00000047508 | *ZNF580* |
| ENSBTAG00000047529 | *ENSBTAG00000047529* |
| ENSBTAG00000047547 | *ENSBTAG00000047547* |
| ENSBTAG00000047548 | *ENSBTAG00000047548* |
| ENSBTAG00000047586 | *NPY1R* |
| ENSBTAG00000047591 | *C23H6ORF141* |
| ENSBTAG00000047772 | *FBXO48* |
| ENSBTAG00000047795 | *ENSBTAG00000047795* |
| ENSBTAG00000047866 | *ENSBTAG00000047866* |
| ENSBTAG00000047902 | *ULBP21* |
| ENSBTAG00000047926 | *OVCA2* |
| ENSBTAG00000047947 | *ENSBTAG00000047947* |
| ENSBTAG00000048020 | *ENSBTAG00000048020* |
| ENSBTAG00000048049 | *ENSBTAG00000048049* |
| ENSBTAG00000048080 | *ENSBTAG00000048080* |
| ENSBTAG00000048120 | *ENSBTAG00000048120* |
| ENSBTAG00000048156 | *ENSBTAG00000048156* |
| ENSBTAG00000048167 | *ENSBTAG00000048167* |
| ENSBTAG00000048185 | *ENSBTAG00000048185* |
| ENSBTAG00000048206 | *ENSBTAG00000048206* |
| ENSBTAG00000048226 | *ENSBTAG00000048226* |
| ENSBTAG00000048269 | *ENSBTAG00000048269* |
| ENSBTAG00000048287 | *ENSBTAG00000048287* |
| ENSBTAG00000048293 | *ENSBTAG00000048293* |
| ENSBTAG00000000009 | *FOXF1* |
| ENSBTAG00000000040 | *MAFG* |
| ENSBTAG00000000052 | *PKNOX2* |
| ENSBTAG00000000054 | *SNAPC4* |
| ENSBTAG00000000074 | *NFIA* |
| ENSBTAG00000000098 | *SETDB1* |
| ENSBTAG00000000176 | *SP3* |
| ENSBTAG00000000195 | *ENSBTAG00000000195* |
| ENSBTAG00000000222 | *ARID4B* |
| ENSBTAG00000000332 | *ERF* |
| ENSBTAG00000000340 | *ADNP* |
| ENSBTAG00000000375 | *SSRP1* |
| ENSBTAG00000000385 | *ZBTB18* |
| ENSBTAG00000000389 | *ZNF212* |
| ENSBTAG00000000422 | *TSHZ1* |
| ENSBTAG00000000455 | *CREBZF* |
| ENSBTAG00000000472 | *ZNF570* |
| ENSBTAG00000000507 | *NR4A1* |
| ENSBTAG00000000533 | *ZNF684* |
| ENSBTAG00000000536 | *ZNF395* |
| ENSBTAG00000000565 | *RFX7* |
| ENSBTAG00000000569 | *HES1* |
| ENSBTAG00000000602 | *RXRB* |
| ENSBTAG00000000623 | *ENSBTAG00000000623* |
| ENSBTAG00000000625 | *SMAD6* |
| ENSBTAG00000000656 | *NFATC1* |
| ENSBTAG00000000678 | *CSDE1* |
| ENSBTAG00000000753 | *PIAS4* |
| ENSBTAG00000000801 | *ZNF583* |
| ENSBTAG00000000803 | *ZNF667* |
| ENSBTAG00000000809 | *IRX1* |
| ENSBTAG00000000816 | *PRDM1* |
| ENSBTAG00000000819 | *RFX3* |
| ENSBTAG00000000868 | *ZFP1* |
| ENSBTAG00000000919 | *HEY2* |
| ENSBTAG00000000943 | *ZNF286A* |
| ENSBTAG00000001002 | *TCF7* |
| ENSBTAG00000001024 | *ARID1A* |
| ENSBTAG00000001042 | *MXD1* |
| ENSBTAG00000001063 | *HOXA4* |
| ENSBTAG00000001069 | *TP53* |
| ENSBTAG00000001146 | *HIVEP2* |
| ENSBTAG00000001160 | *GMEB1* |
| ENSBTAG00000001252 | *HIVEP1* |
| ENSBTAG00000001292 | *LTF* |
| ENSBTAG00000001333 | *PPARG* |
| ENSBTAG00000001385 | *ZSCAN2* |
| ENSBTAG00000001439 | *HESX1* |
| ENSBTAG00000001441 | *ENSBTAG00000001441* |
| ENSBTAG00000001455 | *HOXA7* |
| ENSBTAG00000001486 | *ENSBTAG00000001486* |
| ENSBTAG00000001509 | *ELK3* |
| ENSBTAG00000001511 | *BCL6* |
| ENSBTAG00000001512 | *ZFP64* |
| ENSBTAG00000001562 | *NFE2* |
| ENSBTAG00000001614 | *ZNF174* |
| ENSBTAG00000001616 | *ZNF592* |
| ENSBTAG00000001649 | *ZFPM2* |
| ENSBTAG00000001762 | *RFXANK* |
| ENSBTAG00000001818 | *MEF2B* |
| ENSBTAG00000001864 | *NR4A3* |
| ENSBTAG00000001904 | *ZNF276* |
| ENSBTAG00000001919 | *ZNF652* |
| ENSBTAG00000001947 | *ZNF514* |
| ENSBTAG00000001949 | *ENSBTAG00000001949* |
| ENSBTAG00000002020 | *CREBRF* |
| ENSBTAG00000002055 | *ZFP28* |
| ENSBTAG00000002113 | *ZNF41* |
| ENSBTAG00000002129 | *KLF5* |
| ENSBTAG00000002201 | *NFXL1* |
| ENSBTAG00000002256 | *YEATS4* |
| ENSBTAG00000002281 | *ZNF354C* |
| ENSBTAG00000002291 | *ZBTB41* |
| ENSBTAG00000002292 | *SMARCA1* |
| ENSBTAG00000002295 | *ATF2* |
| ENSBTAG00000002333 | *HOPX* |
| ENSBTAG00000002336 | *RCOR1* |
| ENSBTAG00000002341 | *ETS1* |
| ENSBTAG00000002368 | *TULP2* |
| ENSBTAG00000002370 | *ZNF792* |
| ENSBTAG00000002398 | *ZNF567* |
| ENSBTAG00000002445 | *ENSBTAG00000002445* |
| ENSBTAG00000002586 | *TCF12* |
| ENSBTAG00000002594 | *ZNF436* |
| ENSBTAG00000002613 | *MIS18BP1* |
| ENSBTAG00000002668 | *ZNF711* |
| ENSBTAG00000002687 | *TSHZ3* |
| ENSBTAG00000002690 | *BLZF1* |
| ENSBTAG00000002728 | *ARID1B* |
| ENSBTAG00000002810 | *ZNF777* |
| ENSBTAG00000002835 | *SMAD1* |
| ENSBTAG00000002904 | *ZNF787* |
| ENSBTAG00000002929 | *IRF4* |
| ENSBTAG00000002936 | *PRRX2* |
| ENSBTAG00000002956 | *ZNF674* |
| ENSBTAG00000003021 | *SP1* |
| ENSBTAG00000003027 | *EMX2* |
| ENSBTAG00000003034 | *GATAD2A* |
| ENSBTAG00000003047 | *FAM170A* |
| ENSBTAG00000003064 | *PAXBP1* |
| ENSBTAG00000003120 | *ZNF385B* |
| ENSBTAG00000003172 | *MEIS2* |
| ENSBTAG00000003238 | *MEOX2* |
| ENSBTAG00000003264 | *NR1H2* |
| ENSBTAG00000003267 | *ZNF132* |
| ENSBTAG00000003278 | *HOXC10* |
| ENSBTAG00000003304 | *HOXC4* |
| ENSBTAG00000003339 | *ZBTB10* |
| ENSBTAG00000003396 | *MAFB* |
| ENSBTAG00000003399 | *SMARCA5* |
| ENSBTAG00000003438 | *ZBTB43* |
| ENSBTAG00000003447 | *ENSBTAG00000003447* |
| ENSBTAG00000003456 | *ZNF606* |
| ENSBTAG00000003457 | *ATF5* |
| ENSBTAG00000003462 | *ZNF135* |
| ENSBTAG00000003465 | *ZNF329* |
| ENSBTAG00000003514 | *HSF4* |
| ENSBTAG00000003541 | *ZNF614* |
| ENSBTAG00000003546 | *TFAM* |
| ENSBTAG00000003553 | *ZFP36L2* |
| ENSBTAG00000003569 | *DZIP1* |
| ENSBTAG00000003575 | *ZKSCAN4* |
| ENSBTAG00000003602 | *RBPJ* |
| ENSBTAG00000003606 | *GLI4* |
| ENSBTAG00000003622 | *ZKSCAN7* |
| ENSBTAG00000003638 | *ZNF215* |
| ENSBTAG00000003650 | *NR4A2* |
| ENSBTAG00000003653 | *LYAR* |
| ENSBTAG00000003669 | *BNC2* |
| ENSBTAG00000003687 | *FOXK2* |
| ENSBTAG00000003699 | *ZFP3* |
| ENSBTAG00000003711 | *EPAS1* |
| ENSBTAG00000003784 | *DNAJC2* |
| ENSBTAG00000003801 | *MBD1* |
| ENSBTAG00000003843 | *SMARCAL1* |
| ENSBTAG00000003845 | *CSRNP1* |
| ENSBTAG00000003902 | *ZNF512* |
| ENSBTAG00000003953 | *ZNF316* |
| ENSBTAG00000003968 | *UBP1* |
| ENSBTAG00000003971 | *E2F1* |
| ENSBTAG00000004004 | *CSDC2* |
| ENSBTAG00000004022 | *PLAG1* |
| ENSBTAG00000004029 | *ENSBTAG00000004029* |
| ENSBTAG00000004037 | *JUN* |
| ENSBTAG00000004073 | *MTA3* |
| ENSBTAG00000004104 | *RUNX2* |
| ENSBTAG00000004136 | *NFE2L3* |
| ENSBTAG00000004139 | *BACH1* |
| ENSBTAG00000004159 | *SIX2* |
| ENSBTAG00000004189 | *MLXIP* |
| ENSBTAG00000004193 | *VEZF1* |
| ENSBTAG00000004262 | *ZNF454* |
| ENSBTAG00000004322 | *FOS* |
| ENSBTAG00000004361 | *ZBTB37* |
| ENSBTAG00000004368 | *NFATC3* |
| ENSBTAG00000004380 | *STAT2* |
| ENSBTAG00000004468 | *BARX1* |
| ENSBTAG00000004538 | *PRDM9* |
| ENSBTAG00000004570 | *PRRX1* |
| ENSBTAG00000004602 | *PITX1* |
| ENSBTAG00000004604 | *CCDC124* |
| ENSBTAG00000004620 | *ZNF133* |
| ENSBTAG00000004650 | *ZNF516* |
| ENSBTAG00000004675 | *ZNF18* |
| ENSBTAG00000004742 | *RUNX1* |
| ENSBTAG00000004822 | *SOX10* |
| ENSBTAG00000004835 | *HOXD3* |
| ENSBTAG00000004838 | *IRX5* |
| ENSBTAG00000004862 | *TUB* |
| ENSBTAG00000004879 | *FOXO4* |
| ENSBTAG00000004925 | *ENSBTAG00000004925* |
| ENSBTAG00000004939 | *ZNF569* |
| ENSBTAG00000004953 | *ELK4* |
| ENSBTAG00000004954 | *TOX* |
| ENSBTAG00000004989 | *IRF5* |
| ENSBTAG00000005010 | *MXD3* |
| ENSBTAG00000005017 | *ZNF169* |
| ENSBTAG00000005029 | *TAL1* |
| ENSBTAG00000005038 | *ZBTB42* |
| ENSBTAG00000005067 | *UBTF* |
| ENSBTAG00000005083 | *TULP4* |
| ENSBTAG00000005150 | *HMGB3* |
| ENSBTAG00000005195 | *ZNF200* |
| ENSBTAG00000005227 | *ATF6* |
| ENSBTAG00000005230 | *SHOX2* |
| ENSBTAG00000005240 | *ZNF605* |
| ENSBTAG00000005286 | *ENSBTAG00000005286* |
| ENSBTAG00000005354 | *LRRFIP1* |
| ENSBTAG00000005404 | *MSC* |
| ENSBTAG00000005425 | *GATA4* |
| ENSBTAG00000005443 | *MIER1* |
| ENSBTAG00000005461 | *SOX15* |
| ENSBTAG00000005474 | *CREB1* |
| ENSBTAG00000005478 | *PATZ1* |
| ENSBTAG00000005524 | *HBP1* |
| ENSBTAG00000005525 | *LHX6* |
| ENSBTAG00000005546 | *HOXB7* |
| ENSBTAG00000005564 | *PIAS1* |
| ENSBTAG00000005572 | *ZNF205* |
| ENSBTAG00000005606 | *HOXC9* |
| ENSBTAG00000005622 | *LITAF* |
| ENSBTAG00000005734 | *GATA6* |
| ENSBTAG00000005748 | *SOX17* |
| ENSBTAG00000005750 | *TCF15* |
| ENSBTAG00000005816 | *IRF9* |
| ENSBTAG00000005852 | *KLF8* |
| ENSBTAG00000005863 | *ENSBTAG00000005863* |
| ENSBTAG00000005866 | *ZNF81* |
| ENSBTAG00000005871 | *MECOM* |
| ENSBTAG00000005882 | *ENSBTAG00000005882* |
| ENSBTAG00000005916 | *ADNP2* |
| ENSBTAG00000005951 | *HIC2* |
| ENSBTAG00000005970 | *XBP1* |
| ENSBTAG00000005980 | *RREB1* |
| ENSBTAG00000006017 | *NFKB2* |
| ENSBTAG00000006068 | *CC2D1A* |
| ENSBTAG00000006083 | *KLF1* |
| ENSBTAG00000006143 | *CREB3L1* |
| ENSBTAG00000006170 | *ZNF76* |
| ENSBTAG00000006194 | *FOSL1* |
| ENSBTAG00000006212 | *GTF2IRD1* |
| ENSBTAG00000006222 | *TFDP2* |
| ENSBTAG00000006335 | *STAT6* |
| ENSBTAG00000006364 | *GLMP* |
| ENSBTAG00000006366 | *NFATC4* |
| ENSBTAG00000006404 | *CENPT* |
| ENSBTAG00000006411 | *THAP11* |
| ENSBTAG00000006428 | *ENSBTAG00000006428* |
| ENSBTAG00000006511 | *MTF1* |
| ENSBTAG00000006587 | *ZNF367* |
| ENSBTAG00000006618 | *HLF* |
| ENSBTAG00000006631 | *GLI1* |
| ENSBTAG00000006633 | *IRF3* |
| ENSBTAG00000006650 | *ZNF668* |
| ENSBTAG00000006678 | *GATAD2B* |
| ENSBTAG00000006679 | *MITF* |
| ENSBTAG00000006729 | *ENSBTAG00000006729* |
| ENSBTAG00000006754 | *DBP* |
| ENSBTAG00000006805 | *ZNF180* |
| ENSBTAG00000006844 | *LEF1* |
| ENSBTAG00000006862 | *MEIS3* |
| ENSBTAG00000006893 | *BBX* |
| ENSBTAG00000006919 | *SMAD4* |
| ENSBTAG00000006954 | *ZNF432* |
| ENSBTAG00000006990 | *MYRF* |
| ENSBTAG00000007012 | *ZNF3* |
| ENSBTAG00000007020 | *ZNF502* |
| ENSBTAG00000007053 | *ZFHX2* |
| ENSBTAG00000007074 | *ZKSCAN8* |
| ENSBTAG00000007080 | *PREB* |
| ENSBTAG00000007110 | *RCOR3* |
| ENSBTAG00000007122 | *ZFP2* |
| ENSBTAG00000007159 | *ESR1* |
| ENSBTAG00000007190 | *THAP6* |
| ENSBTAG00000007318 | *ZNF687* |
| ENSBTAG00000007329 | *SETDB2* |
| ENSBTAG00000007356 | *ELF1* |
| ENSBTAG00000007383 | *ZNF521* |
| ENSBTAG00000007485 | *GRHL1* |
| ENSBTAG00000007488 | *ZFP90* |
| ENSBTAG00000007498 | *ZGPAT* |
| ENSBTAG00000007589 | *SMAD9* |
| ENSBTAG00000007592 | *RARG* |
| ENSBTAG00000007617 | *TERF2* |
| ENSBTAG00000007629 | *THAP1* |
| ENSBTAG00000007660 | *ZNF414* |
| ENSBTAG00000007678 | *MKX* |
| ENSBTAG00000007714 | *ZNF235* |
| ENSBTAG00000007718 | *TGIF1* |
| ENSBTAG00000007730 | *ZFX* |
| ENSBTAG00000007746 | *AHR* |
| ENSBTAG00000007761 | *ZBTB5* |
| ENSBTAG00000007783 | *MYBL2* |
| ENSBTAG00000007802 | *BCLAF1* |
| ENSBTAG00000007827 | *RCOR2* |
| ENSBTAG00000007833 | *PIAS2* |
| ENSBTAG00000007867 | *STAT1* |
| ENSBTAG00000007884 | *SREBF1* |
| ENSBTAG00000007917 | *TSHZ2* |
| ENSBTAG00000007983 | *ZNF775* |
| ENSBTAG00000007986 | *NSD2* |
| ENSBTAG00000008048 | *GCFC2* |
| ENSBTAG00000008054 | *ZNF706* |
| ENSBTAG00000008062 | *DMRT2* |
| ENSBTAG00000008063 | *PPARA* |
| ENSBTAG00000008084 | *ZNF382* |
| ENSBTAG00000008113 | *OSR1* |
| ENSBTAG00000008132 | *SOX13* |
| ENSBTAG00000008138 | *HOXA2* |
| ENSBTAG00000008139 | *HOXA3* |
| ENSBTAG00000008175 | *ZNF713* |
| ENSBTAG00000008182 | *FOSB* |
| ENSBTAG00000008272 | *EBF3* |
| ENSBTAG00000008280 | *HNF4G* |
| ENSBTAG00000008283 | *FLI1* |
| ENSBTAG00000008289 | *ZBTB4* |
| ENSBTAG00000008313 | *KLF15* |
| ENSBTAG00000008333 | *ETV4* |
| ENSBTAG00000008349 | *ZNF311* |
| ENSBTAG00000008397 | *ZNF555* |
| ENSBTAG00000008409 | *MYC* |
| ENSBTAG00000008448 | *MEIS1* |
| ENSBTAG00000008482 | *SON* |
| ENSBTAG00000008520 | *NFIC* |
| ENSBTAG00000008523 | *TFE3* |
| ENSBTAG00000008545 | *ATF3* |
| ENSBTAG00000008556 | *POU2F2* |
| ENSBTAG00000008573 | *ZFP36* |
| ENSBTAG00000008591 | *CAMTA2* |
| ENSBTAG00000008607 | *ARID3A* |
| ENSBTAG00000008645 | *ESRRA* |
| ENSBTAG00000008649 | *ETV3* |
| ENSBTAG00000008688 | *HEYL* |
| ENSBTAG00000008695 | *TCF3* |
| ENSBTAG00000008756 | *ELF3* |
| ENSBTAG00000008771 | *MYEF2* |
| ENSBTAG00000008794 | *ATF6B* |
| ENSBTAG00000008810 | *GPBP1L1* |
| ENSBTAG00000008836 | *ZNF782* |
| ENSBTAG00000008881 | *PURA* |
| ENSBTAG00000008883 | *ZNF207* |
| ENSBTAG00000008902 | *ZNF345* |
| ENSBTAG00000008908 | *GZF1* |
| ENSBTAG00000008911 | *ENSBTAG00000008911* |
| ENSBTAG00000008943 | *ZSCAN12* |
| ENSBTAG00000009059 | *PITX2* |
| ENSBTAG00000009071 | *ENSBTAG00000009071* |
| ENSBTAG00000009072 | *ZNF572* |
| ENSBTAG00000009126 | *YBX2* |
| ENSBTAG00000009152 | *ZNF391* |
| ENSBTAG00000009153 | *MLXIPL* |
| ENSBTAG00000009199 | *GLIS2* |
| ENSBTAG00000009206 | *FOXS1* |
| ENSBTAG00000009210 | *ZBTB44* |
| ENSBTAG00000009214 | *ETS2* |
| ENSBTAG00000009215 | *NR1I3* |
| ENSBTAG00000009238 | *HOXC6* |
| ENSBTAG00000009265 | *NR5A2* |
| ENSBTAG00000009272 | *ELMSAN1* |
| ENSBTAG00000009378 | *E2F5* |
| ENSBTAG00000009451 | *JDP2* |
| ENSBTAG00000009460 | *ENSBTAG00000009460* |
| ENSBTAG00000009482 | *AFF4* |
| ENSBTAG00000009496 | *STAT5A* |
| ENSBTAG00000009521 | *ZNF565* |
| ENSBTAG00000009631 | *ZNF683* |
| ENSBTAG00000009634 | *HOXC5* |
| ENSBTAG00000009643 | *ZBTB25* |
| ENSBTAG00000009658 | *PLEK* |
| ENSBTAG00000009663 | *YBX3* |
| ENSBTAG00000009734 | *XPA* |
| ENSBTAG00000009780 | *GTF2I* |
| ENSBTAG00000009863 | *BHLHE40* |
| ENSBTAG00000009870 | *ENSBTAG00000009870* |
| ENSBTAG00000009874 | *ZXDC* |
| ENSBTAG00000009889 | *ZNF181* |
| ENSBTAG00000009895 | *ZNF599* |
| ENSBTAG00000009905 | *NFYA* |
| ENSBTAG00000009961 | *E4F1* |
| ENSBTAG00000009979 | *HOXB2* |
| ENSBTAG00000010002 | *IRF2* |
| ENSBTAG00000010046 | *ENSBTAG00000010046* |
| ENSBTAG00000010069 | *EGR1* |
| ENSBTAG00000010111 | *TCF7L1* |
| ENSBTAG00000010113 | *ZBTB24* |
| ENSBTAG00000010125 | *STAT5B* |
| ENSBTAG00000010130 | *ZNF335* |
| ENSBTAG00000010215 | *CREB3L3* |
| ENSBTAG00000010217 | *ZNF318* |
| ENSBTAG00000010252 | *ENSBTAG00000010252* |
| ENSBTAG00000010255 | *ZNF24* |
| ENSBTAG00000010291 | *RBAK* |
| ENSBTAG00000010392 | *ESRRG* |
| ENSBTAG00000010450 | *WIZ* |
| ENSBTAG00000010533 | *HMGXB4* |
| ENSBTAG00000010568 | *ZBED5* |
| ENSBTAG00000010649 | *MEF2A* |
| ENSBTAG00000010659 | *CUX1* |
| ENSBTAG00000010671 | *GLI3* |
| ENSBTAG00000010681 | *NR1H3* |
| ENSBTAG00000010745 | *THRA* |
| ENSBTAG00000010818 | *AEBP2* |
| ENSBTAG00000010819 | *HMBOX1* |
| ENSBTAG00000010829 | *ENSBTAG00000010829* |
| ENSBTAG00000010875 | *MSX1* |
| ENSBTAG00000010928 | *CSRNP2* |
| ENSBTAG00000010978 | *PROX1* |
| ENSBTAG00000011001 | *ERG* |
| ENSBTAG00000011003 | *IKZF3* |
| ENSBTAG00000011052 | *ENSBTAG00000011052* |
| ENSBTAG00000011074 | *BRD2* |
| ENSBTAG00000011087 | *ARID2* |
| ENSBTAG00000011136 | *NR2F6* |
| ENSBTAG00000011162 | *ZKSCAN2* |
| ENSBTAG00000011200 | *ZNF644* |
| ENSBTAG00000011214 | *THAP3* |
| ENSBTAG00000011234 | *FOXO3* |
| ENSBTAG00000011246 | *FOXP2* |
| ENSBTAG00000011262 | *ENSBTAG00000011262* |
| ENSBTAG00000011266 | *ZBTB16* |
| ENSBTAG00000011429 | *CREB3* |
| ENSBTAG00000011467 | *BATF2* |
| ENSBTAG00000011498 | *ZNF184* |
| ENSBTAG00000011518 | *RARB* |
| ENSBTAG00000011646 | *ZNF512B* |
| ENSBTAG00000011662 | *SOX18* |
| ENSBTAG00000011682 | *GLI2* |
| ENSBTAG00000011789 | *REST* |
| ENSBTAG00000011816 | *TBX10* |
| ENSBTAG00000011828 | *ARID3B* |
| ENSBTAG00000011844 | *ENSBTAG00000011844* |
| ENSBTAG00000011865 | *MTA2* |
| ENSBTAG00000011926 | *ENSBTAG00000011926* |
| ENSBTAG00000011928 | *ZBTB20* |
| ENSBTAG00000011957 | *CREBL2* |
| ENSBTAG00000011981 | *MYBL1* |
| ENSBTAG00000011982 | *BATF3* |
| ENSBTAG00000011997 | *ZMIZ2* |
| ENSBTAG00000012025 | *LMX1A* |
| ENSBTAG00000012046 | *JUNB* |
| ENSBTAG00000012063 | *E2F4* |
| ENSBTAG00000012064 | *ZNF473* |
| ENSBTAG00000012074 | *MYB* |
| ENSBTAG00000012083 | *IRX3* |
| ENSBTAG00000012102 | *TCF25* |
| ENSBTAG00000012111 | *PRDM5* |
| ENSBTAG00000012116 | *HNF4A* |
| ENSBTAG00000012124 | *GPBP1* |
| ENSBTAG00000012139 | *SIX1* |
| ENSBTAG00000012149 | *HOXC8* |
| ENSBTAG00000012159 | *CNBP* |
| ENSBTAG00000012178 | *NR1D1* |
| ENSBTAG00000012211 | *HOXA5* |
| ENSBTAG00000012242 | *MAF1* |
| ENSBTAG00000012271 | *ENSBTAG00000012271* |
| ENSBTAG00000012274 | *CIZ1* |
| ENSBTAG00000012353 | *ZNF34* |
| ENSBTAG00000012384 | *TFEB* |
| ENSBTAG00000012385 | *NFX1* |
| ENSBTAG00000012416 | *ZNF511* |
| ENSBTAG00000012436 | *HES7* |
| ENSBTAG00000012441 | *SMAD7* |
| ENSBTAG00000012449 | *AFF3* |
| ENSBTAG00000012460 | *ELF5* |
| ENSBTAG00000012463 | *ZMAT3* |
| ENSBTAG00000012465 | *FOXC2* |
| ENSBTAG00000012500 | *RARA* |
| ENSBTAG00000012522 | *ZNF283* |
| ENSBTAG00000012599 | *SMAD3* |
| ENSBTAG00000012600 | *GTF3A* |
| ENSBTAG00000012615 | *ZEB2* |
| ENSBTAG00000012629 | *ZNF362* |
| ENSBTAG00000012636 | *IKZF4* |
| ENSBTAG00000012738 | *ZNF827* |
| ENSBTAG00000012768 | *HMGXB3* |
| ENSBTAG00000012777 | *SRF* |
| ENSBTAG00000012868 | *ENSBTAG00000012868* |
| ENSBTAG00000012915 | *ZNF404* |
| ENSBTAG00000012936 | *THYN1* |
| ENSBTAG00000012938 | *JARID2* |
| ENSBTAG00000012946 | *HSF2* |
| ENSBTAG00000012999 | *PRDM4* |
| ENSBTAG00000013001 | *ZNF84* |
| ENSBTAG00000013029 | *ARNTL* |
| ENSBTAG00000013050 | *ZNF277* |
| ENSBTAG00000013142 | *MYNN* |
| ENSBTAG00000013213 | *OSR2* |
| ENSBTAG00000013227 | *SNAI2* |
| ENSBTAG00000013253 | *THAP4* |
| ENSBTAG00000013263 | *HOXA1* |
| ENSBTAG00000013271 | *NCOR1* |
| ENSBTAG00000013279 | *FOXP3* |
| ENSBTAG00000013306 | *ZBTB7C* |
| ENSBTAG00000013314 | *PBX3* |
| ENSBTAG00000013346 | *SIX5* |
| ENSBTAG00000013353 | *ZNF274* |
| ENSBTAG00000013412 | *NFAT5* |
| ENSBTAG00000013444 | *ETV2* |
| ENSBTAG00000013460 | *ZBTB11* |
| ENSBTAG00000013531 | *CENPS* |
| ENSBTAG00000013588 | *ZNF532* |
| ENSBTAG00000013592 | *ZNF248* |
| ENSBTAG00000013653 | *NFE2L1* |
| ENSBTAG00000013716 | *NR2C2* |
| ENSBTAG00000013740 | *SP2* |
| ENSBTAG00000013757 | *CTCF* |
| ENSBTAG00000013801 | *PBX1* |
| ENSBTAG00000013873 | *MSX2* |
| ENSBTAG00000013895 | *RELA* |
| ENSBTAG00000013949 | *AHCTF1* |
| ENSBTAG00000013961 | *MYSM1* |
| ENSBTAG00000013991 | *NR2E1* |
| ENSBTAG00000014003 | *MYF6* |
| ENSBTAG00000014016 | *IKZF1* |
| ENSBTAG00000014090 | *ARID5A* |
| ENSBTAG00000014123 | *ZNF507* |
| ENSBTAG00000014153 | *PKNOX1* |
| ENSBTAG00000014199 | *TBX21* |
| ENSBTAG00000014211 | *FOXP4* |
| ENSBTAG00000014217 | *HHEX* |
| ENSBTAG00000014228 | *TFEC* |
| ENSBTAG00000014248 | *MIER3* |
| ENSBTAG00000014265 | *SREBF2* |
| ENSBTAG00000014273 | *NRF1* |
| ENSBTAG00000014278 | *TBX2* |
| ENSBTAG00000014389 | *SP4* |
| ENSBTAG00000014396 | *KLF10* |
| ENSBTAG00000014415 | *ZNF653* |
| ENSBTAG00000014421 | *PBX2* |
| ENSBTAG00000014429 | *KMT2D* |
| ENSBTAG00000014435 | *TCF19* |
| ENSBTAG00000014478 | *ZSCAN29* |
| ENSBTAG00000014506 | *ZNF503* |
| ENSBTAG00000014554 | *SNAI1* |
| ENSBTAG00000014560 | *HLX* |
| ENSBTAG00000014593 | *ENSBTAG00000014593* |
| ENSBTAG00000014605 | *ETV6* |
| ENSBTAG00000014636 | *ZFHX3* |
| ENSBTAG00000014677 | *TADA2A* |
| ENSBTAG00000014680 | *ZBTB49* |
| ENSBTAG00000014692 | *CREB3L4* |
| ENSBTAG00000014697 | *SMARCC2* |
| ENSBTAG00000014705 | *HES4* |
| ENSBTAG00000014749 | *ZIC4* |
| ENSBTAG00000014751 | *ZIC1* |
| ENSBTAG00000014768 | *ZNF786* |
| ENSBTAG00000014769 | *GMEB2* |
| ENSBTAG00000014786 | *PBRM1* |
| ENSBTAG00000014790 | *ZBTB2* |
| ENSBTAG00000014813 | *ENSBTAG00000014813* |
| ENSBTAG00000014862 | *ENSBTAG00000014862* |
| ENSBTAG00000014915 | *ETV5* |
| ENSBTAG00000014967 | *PAX7* |
| ENSBTAG00000014977 | *ALX1* |
| ENSBTAG00000014995 | *THAP8* |
| ENSBTAG00000015007 | *NCOA1* |
| ENSBTAG00000015015 | *HINFP* |
| ENSBTAG00000015052 | *PROX2* |
| ENSBTAG00000015101 | *HMGB2* |
| ENSBTAG00000015138 | *MGA* |
| ENSBTAG00000015146 | *TOX4* |
| ENSBTAG00000015151 | *FOXN2* |
| ENSBTAG00000015209 | *MXD4* |
| ENSBTAG00000015308 | *SMARCE1* |
| ENSBTAG00000015321 | *TRERF1* |
| ENSBTAG00000015334 | *ZHX1* |
| ENSBTAG00000015348 | *ZNF189* |
| ENSBTAG00000015371 | *GABPB2* |
| ENSBTAG00000015386 | *REL* |
| ENSBTAG00000015462 | *BNC1* |
| ENSBTAG00000015504 | *ZNF131* |
| ENSBTAG00000015554 | *TMF1* |
| ENSBTAG00000015604 | *ZNF385A* |
| ENSBTAG00000015702 | *PITX3* |
| ENSBTAG00000015717 | *HEY1* |
| ENSBTAG00000015751 | *MEOX1* |
| ENSBTAG00000015766 | *ZFPM1* |
| ENSBTAG00000015802 | *CREB3L2* |
| ENSBTAG00000015808 | *ZNF609* |
| ENSBTAG00000015817 | *ELK1* |
| ENSBTAG00000015840 | *HOXD1* |
| ENSBTAG00000015866 | *ENSBTAG00000015866* |
| ENSBTAG00000015879 | *TULP3* |
| ENSBTAG00000015887 | *FOXJ3* |
| ENSBTAG00000015899 | *ENSBTAG00000015899* |
| ENSBTAG00000015904 | *RORA* |
| ENSBTAG00000015981 | *ETV1* |
| ENSBTAG00000016000 | *CARF* |
| ENSBTAG00000016028 | *ZNF691* |
| ENSBTAG00000016033 | *HOXD9* |
| ENSBTAG00000016060 | *CREM* |
| ENSBTAG00000016074 | *ZNF143* |
| ENSBTAG00000016077 | *ZNF317* |
| ENSBTAG00000016103 | *CBFB* |
| ENSBTAG00000016137 | *ZNF608* |
| ENSBTAG00000016169 | *ID1* |
| ENSBTAG00000016190 | *ZNF394* |
| ENSBTAG00000016191 | *ZNF32* |
| ENSBTAG00000016203 | *ZNF16* |
| ENSBTAG00000016229 | *KLF9* |
| ENSBTAG00000016257 | *ZNF74* |
| ENSBTAG00000016294 | *ZNF438* |
| ENSBTAG00000016299 | *ZNF784* |
| ENSBTAG00000016328 | *SFPQ* |
| ENSBTAG00000016349 | *TEAD2* |
| ENSBTAG00000016362 | *POU6F1* |
| ENSBTAG00000016414 | *VDR* |
| ENSBTAG00000016438 | *TERB1* |
| ENSBTAG00000016441 | *ZNF622* |
| ENSBTAG00000016448 | *ZBTB40* |
| ENSBTAG00000016462 | *TCF4* |
| ENSBTAG00000016496 | *ZNF641* |
| ENSBTAG00000016502 | *ENSBTAG00000016502* |
| ENSBTAG00000016513 | *ZNF331* |
| ENSBTAG00000016533 | *FOXP1* |
| ENSBTAG00000016546 | *PARP12* |
| ENSBTAG00000016580 | *TEF* |
| ENSBTAG00000016592 | *CAPN15* |
| ENSBTAG00000016651 | *ISL2* |
| ENSBTAG00000016664 | *SLC2A4RG* |
| ENSBTAG00000016667 | *ZBTB46* |
| ENSBTAG00000016684 | *ZNF202* |
| ENSBTAG00000016721 | *ENSBTAG00000016721* |
| ENSBTAG00000016723 | *GFI1* |
| ENSBTAG00000016735 | *ZBTB48* |
| ENSBTAG00000016757 | *ZKSCAN1* |
| ENSBTAG00000016760 | *LRRFIP2* |
| ENSBTAG00000016801 | *RXRG* |
| ENSBTAG00000016829 | *ZBTB22* |
| ENSBTAG00000017002 | *RBCK1* |
| ENSBTAG00000017035 | *USF2* |
| ENSBTAG00000017072 | *ZNF384* |
| ENSBTAG00000017120 | *PHB* |
| ENSBTAG00000017123 | *ZNF354A* |
| ENSBTAG00000017184 | *ZBTB33* |
| ENSBTAG00000017243 | *GATA3* |
| ENSBTAG00000017263 | *MXI1* |
| ENSBTAG00000017306 | *AKNA* |
| ENSBTAG00000017365 | *ZBTB47* |
| ENSBTAG00000017368 | *YBX1* |
| ENSBTAG00000017393 | *L3MBTL3* |
| ENSBTAG00000017397 | *ZNF423* |
| ENSBTAG00000017405 | *RORC* |
| ENSBTAG00000017409 | *DLX3* |
| ENSBTAG00000017419 | *ZZZ3* |
| ENSBTAG00000017462 | *ATF4* |
| ENSBTAG00000017488 | *KLF3* |
| ENSBTAG00000017517 | *ZNF236* |
| ENSBTAG00000017542 | *PPARD* |
| ENSBTAG00000017560 | *ENSBTAG00000017560* |
| ENSBTAG00000017580 | *RFX5* |
| ENSBTAG00000017594 | *ZHX3* |
| ENSBTAG00000017599 | *NR2F1* |
| ENSBTAG00000017610 | *NFRKB* |
| ENSBTAG00000017613 | *ZNF419* |
| ENSBTAG00000017651 | *ENSBTAG00000017651* |
| ENSBTAG00000017661 | *RFX2* |
| ENSBTAG00000017682 | *TET2* |
| ENSBTAG00000017694 | *TRPS1* |
| ENSBTAG00000017729 | *NR2C1* |
| ENSBTAG00000017731 | *ZFAT* |
| ENSBTAG00000017763 | *ENSBTAG00000017763* |
| ENSBTAG00000017800 | *DMRT3* |
| ENSBTAG00000017802 | *THRB* |
| ENSBTAG00000017824 | *IRF8* |
| ENSBTAG00000017836 | *ZFP57* |
| ENSBTAG00000017840 | *BAZ2A* |
| ENSBTAG00000017851 | *RXRA* |
| ENSBTAG00000017872 | *ZNF304* |
| ENSBTAG00000017873 | *USF1* |
| ENSBTAG00000017994 | *MAX* |
| ENSBTAG00000018007 | *NR2F2* |
| ENSBTAG00000018024 | *NR1D2* |
| ENSBTAG00000018057 | *ENSBTAG00000018057* |
| ENSBTAG00000018088 | *SETBP1* |
| ENSBTAG00000018090 | *ZNF449* |
| ENSBTAG00000018094 | *ZNF689* |
| ENSBTAG00000018103 | *HMGB1* |
| ENSBTAG00000018131 | *ATF1* |
| ENSBTAG00000018161 | *TBX18* |
| ENSBTAG00000018199 | *ZBTB8A* |
| ENSBTAG00000018229 | *NFIX* |
| ENSBTAG00000018270 | *NFATC2* |
| ENSBTAG00000018272 | *RERE* |
| ENSBTAG00000018334 | *PLAGL2* |
| ENSBTAG00000018402 | *TSC22D2* |
| ENSBTAG00000018456 | *ZNF7* |
| ENSBTAG00000018488 | *AFF1* |
| ENSBTAG00000018625 | *ZNF263* |
| ENSBTAG00000018645 | *DLX5* |
| ENSBTAG00000018710 | *TTF1* |
| ENSBTAG00000018730 | *ZBTB17* |
| ENSBTAG00000018795 | *PMS1* |
| ENSBTAG00000018898 | *ARID4A* |
| ENSBTAG00000018902 | *ZC2HC1A* |
| ENSBTAG00000018909 | *CREB5* |
| ENSBTAG00000018938 | *CDIP1* |
| ENSBTAG00000018960 | *ENSBTAG00000018960* |
| ENSBTAG00000018965 | *PRDM16* |
| ENSBTAG00000019001 | *PRDM10* |
| ENSBTAG00000019020 | *TAX1BP1* |
| ENSBTAG00000019039 | *ZNF239* |
| ENSBTAG00000019043 | *GABPA* |
| ENSBTAG00000019061 | *ELF4* |
| ENSBTAG00000019120 | *WDHD1* |
| ENSBTAG00000019133 | *ZNF326* |
| ENSBTAG00000019174 | *ZNF710* |
| ENSBTAG00000019208 | *C29H11ORF95* |
| ENSBTAG00000019255 | *NFE2L2* |
| ENSBTAG00000019293 | *EGR3* |
| ENSBTAG00000019303 | *MAFK* |
| ENSBTAG00000019310 | *FOXK1* |
| ENSBTAG00000019312 | *TFCP2* |
| ENSBTAG00000019313 | *ZMIZ1* |
| ENSBTAG00000019329 | *LYL1* |
| ENSBTAG00000019357 | *ZNF688* |
| ENSBTAG00000019359 | *ENSBTAG00000019359* |
| ENSBTAG00000019472 | *NR3C1* |
| ENSBTAG00000019495 | *TEAD3* |
| ENSBTAG00000019518 | *ZNF513* |
| ENSBTAG00000019555 | *ZSCAN20* |
| ENSBTAG00000019557 | *NR1I2* |
| ENSBTAG00000019615 | *PHTF1* |
| ENSBTAG00000019645 | *TFDP1* |
| ENSBTAG00000019695 | *RLF* |
| ENSBTAG00000019697 | *NPAS2* |
| ENSBTAG00000019707 | *GATA2* |
| ENSBTAG00000019742 | *FOXJ2* |
| ENSBTAG00000019767 | *MIER2* |
| ENSBTAG00000019785 | *CIC* |
| ENSBTAG00000019788 | *TEAD4* |
| ENSBTAG00000019800 | *ENSBTAG00000019800* |
| ENSBTAG00000019818 | *CASZ1* |
| ENSBTAG00000019844 | *ZNF467* |
| ENSBTAG00000019885 | *ZNF280C* |
| ENSBTAG00000020004 | *ZNF800* |
| ENSBTAG00000020012 | *CDC5L* |
| ENSBTAG00000020053 | *ZEB1* |
| ENSBTAG00000020117 | *ZBTB7A* |
| ENSBTAG00000020166 | *ZNFX1* |
| ENSBTAG00000020185 | *ENSBTAG00000020185* |
| ENSBTAG00000020270 | *NFKB1* |
| ENSBTAG00000020289 | *ZNF214* |
| ENSBTAG00000020327 | *ZNF410* |
| ENSBTAG00000020355 | *KLF4* |
| ENSBTAG00000020356 | *GON4L* |
| ENSBTAG00000020441 | *HMG20A* |
| ENSBTAG00000020445 | *ZNF398* |
| ENSBTAG00000020448 | *ENSBTAG00000020448* |
| ENSBTAG00000020552 | *PRDM2* |
| ENSBTAG00000020578 | *PRDM6* |
| ENSBTAG00000020594 | *ELF2* |
| ENSBTAG00000020601 | *ZNF366* |
| ENSBTAG00000020643 | *BARX2* |
| ENSBTAG00000020654 | *BAZ2B* |
| ENSBTAG00000020685 | *ENSBTAG00000020685* |
| ENSBTAG00000020701 | *MEF2C* |
| ENSBTAG00000020713 | *BACH2* |
| ENSBTAG00000020737 | *SOX8* |
| ENSBTAG00000020751 | *HSF1* |
| ENSBTAG00000020754 | *ZNF526* |
| ENSBTAG00000020819 | *YY1* |
| ENSBTAG00000020854 | *BCL6B* |
| ENSBTAG00000020878 | *DMTF1* |
| ENSBTAG00000020935 | *HIF1A* |
| ENSBTAG00000020966 | *ALX3* |
| ENSBTAG00000021000 | *USF3* |
| ENSBTAG00000021008 | *ZNF219* |
| ENSBTAG00000021018 | *KHSRP* |
| ENSBTAG00000021037 | *ARNT* |
| ENSBTAG00000021045 | *E2F3* |
| ENSBTAG00000021073 | *ENSBTAG00000021073* |
| ENSBTAG00000021115 | *ZNF692* |
| ENSBTAG00000021158 | *SATB1* |
| ENSBTAG00000021187 | *ID2* |
| ENSBTAG00000021210 | *HES2* |
| ENSBTAG00000021222 | *ZNF771* |
| ENSBTAG00000021253 | *PRDM15* |
| ENSBTAG00000021329 | *ZBTB3* |
| ENSBTAG00000021357 | *ZNF518A* |
| ENSBTAG00000021413 | *ENSBTAG00000021413* |
| ENSBTAG00000021427 | *HOXB3* |
| ENSBTAG00000021433 | *ENSBTAG00000021433* |
| ENSBTAG00000021435 | *MAFF* |
| ENSBTAG00000021444 | *TWIST2* |
| ENSBTAG00000021494 | *EN1* |
| ENSBTAG00000021512 | *ZBTB7B* |
| ENSBTAG00000021523 | *STAT3* |
| ENSBTAG00000021574 | *TCF7L2* |
| ENSBTAG00000021634 | *PIAS3* |
| ENSBTAG00000021645 | *MBD4* |
| ENSBTAG00000021706 | *TBX3* |
| ENSBTAG00000021709 | *SPI1* |
| ENSBTAG00000021742 | *ZNF568* |
| ENSBTAG00000021743 | *ZNF704* |
| ENSBTAG00000021772 | *ENSBTAG00000021772* |
| ENSBTAG00000021789 | *ZNF574* |
| ENSBTAG00000021827 | *TCF21* |
| ENSBTAG00000021869 | *THAP5* |
| ENSBTAG00000021943 | *NFYC* |
| ENSBTAG00000021965 | *SUB1* |
| ENSBTAG00000021996 | *GATAD1* |
| ENSBTAG00000022227 | *PLSCR2* |
| ENSBTAG00000022255 | *AR* |
| ENSBTAG00000022360 | *SOX5* |
| ENSBTAG00000022450 | *THAP9* |
| ENSBTAG00000022887 | *ENSBTAG00000022887* |
| ENSBTAG00000023338 | *ENSBTAG00000023338* |
| ENSBTAG00000023464 | *ENSBTAG00000023464* |
| ENSBTAG00000023611 | *ZBTB32* |
| ENSBTAG00000023675 | *ZNF654* |
| ENSBTAG00000023847 | *IKZF5* |
| ENSBTAG00000023885 | *ZNF79* |
| ENSBTAG00000023929 | *FOSL2* |
| ENSBTAG00000023938 | *CENPA* |
| ENSBTAG00000024000 | *ATOH8* |
| ENSBTAG00000024115 | *FOXO6* |
| ENSBTAG00000024199 | *KMT2C* |
| ENSBTAG00000024341 | *HOXA6* |
| ENSBTAG00000024534 | *POU2F1* |
| ENSBTAG00000024603 | *NCOR2* |
| ENSBTAG00000024641 | *ENSBTAG00000024641* |
| ENSBTAG00000024648 | *PGR* |
| ENSBTAG00000024657 | *ENSBTAG00000024657* |
| ENSBTAG00000025124 | *ZNF300* |
| ENSBTAG00000025129 | *MNT* |
| ENSBTAG00000025130 | *HIC1* |
| ENSBTAG00000025146 | *ENSBTAG00000025146* |
| ENSBTAG00000025405 | *BATF* |
| ENSBTAG00000025434 | *ZFP36L1* |
| ENSBTAG00000025659 | *ZNF618* |
| ENSBTAG00000026263 | *ZNF45* |
| ENSBTAG00000026286 | *GTF2IRD2* |
| ENSBTAG00000026307 | *ZNF629* |
| ENSBTAG00000026309 | *ZHX2* |
| ENSBTAG00000026344 | *MAFA* |
| ENSBTAG00000026408 | *ZNF213* |
| ENSBTAG00000026523 | *PLAGL1* |
| ENSBTAG00000026769 | *ASCL2* |
| ENSBTAG00000026772 | *ZNF142* |
| ENSBTAG00000027058 | *MESP2* |
| ENSBTAG00000027182 | *NR3C2* |
| ENSBTAG00000027431 | *ZNF227* |
| ENSBTAG00000027442 | *NFIB* |
| ENSBTAG00000027557 | *ZNF408* |
| ENSBTAG00000030348 | *ZNF582* |
| ENSBTAG00000030403 | *FIZ1* |
| ENSBTAG00000030425 | *ID3* |
| ENSBTAG00000030470 | *ENSBTAG00000030470* |
| ENSBTAG00000030556 | *ZNF217* |
| ENSBTAG00000030575 | *BHLHE41* |
| ENSBTAG00000030744 | *NFYB* |
| ENSBTAG00000030836 | *ZBTB21* |
| ENSBTAG00000030929 | *ENSBTAG00000030929* |
| ENSBTAG00000030933 | *ZNF576* |
| ENSBTAG00000030939 | *ZNF575* |
| ENSBTAG00000030956 | *ENSBTAG00000030956* |
| ENSBTAG00000031071 | *ZNF624* |
| ENSBTAG00000031106 | *ZNF746* |
| ENSBTAG00000031214 | *ENSBTAG00000031214* |
| ENSBTAG00000031231 | *IRF1* |
| ENSBTAG00000031352 | *ZNF554* |
| ENSBTAG00000031523 | *ENSBTAG00000031523* |
| ENSBTAG00000031544 | *DDIT3* |
| ENSBTAG00000031567 | *SMARCC1* |
| ENSBTAG00000031609 | *THAP12* |
| ENSBTAG00000031682 | *ZNF484* |
| ENSBTAG00000031686 | *ZNF566* |
| ENSBTAG00000031687 | *ENSBTAG00000031687* |
| ENSBTAG00000031741 | *ZNF322* |
| ENSBTAG00000031806 | *ZNF48* |
| ENSBTAG00000031869 | *ZSCAN26* |
| ENSBTAG00000031873 | *ZSCAN16* |
| ENSBTAG00000031874 | *ZNF165* |
| ENSBTAG00000031895 | *TFB1M* |
| ENSBTAG00000032018 | *ZNF19* |
| ENSBTAG00000032031 | *ZBTB12* |
| ENSBTAG00000032187 | *ZNF697* |
| ENSBTAG00000032657 | *TEAD1* |
| ENSBTAG00000032686 | *HOMEZ* |
| ENSBTAG00000032982 | *TERF1* |
| ENSBTAG00000033174 | *TFAP4* |
| ENSBTAG00000033268 | *ZFHX4* |
| ENSBTAG00000033315 | *DNAJC1* |
| ENSBTAG00000033445 | *ENSBTAG00000033445* |
| ENSBTAG00000033563 | *ZNF529* |
| ENSBTAG00000033642 | *ENSBTAG00000033642* |
| ENSBTAG00000034005 | *ZNF35* |
| ENSBTAG00000034529 | *HMGA1* |
| ENSBTAG00000034597 | *ENSBTAG00000034597* |
| ENSBTAG00000036343 | *ENSBTAG00000036343* |
| ENSBTAG00000037375 | *ENSBTAG00000037375* |
| ENSBTAG00000037393 | *ZNF639* |
| ENSBTAG00000037440 | *ENSBTAG00000037440* |
| ENSBTAG00000037508 | *EBF1* |
| ENSBTAG00000037566 | *ZNF500* |
| ENSBTAG00000037581 | *MZF1* |
| ENSBTAG00000037721 | *ENSBTAG00000037721* |
| ENSBTAG00000037757 | *EBF4* |
| ENSBTAG00000037803 | *ZNF197* |
| ENSBTAG00000037804 | *IKZF2* |
| ENSBTAG00000037882 | *ZNF584* |
| ENSBTAG00000037906 | *ENSBTAG00000037906* |
| ENSBTAG00000037981 | *ENSBTAG00000037981* |
| ENSBTAG00000037988 | *ZSCAN31* |
| ENSBTAG00000038034 | *ZNF628* |
| ENSBTAG00000038050 | *ZNF420* |
| ENSBTAG00000038055 | *KLF16* |
| ENSBTAG00000038088 | *ENSBTAG00000038088* |
| ENSBTAG00000038178 | *ZNF25* |
| ENSBTAG00000038240 | *ENSBTAG00000038240* |
| ENSBTAG00000038241 | *REPIN1* |
| ENSBTAG00000038284 | *ENSBTAG00000038284* |
| ENSBTAG00000038428 | *RELB* |
| ENSBTAG00000038487 | *ZNF613* |
| ENSBTAG00000038498 | *ZBTB39* |
| ENSBTAG00000038541 | *ZNF879* |
| ENSBTAG00000038577 | *ENSBTAG00000038577* |
| ENSBTAG00000038610 | *ZBTB26* |
| ENSBTAG00000038635 | *ENSBTAG00000038635* |
| ENSBTAG00000038674 | *ENSBTAG00000038674* |
| ENSBTAG00000038702 | *ENSBTAG00000038702* |
| ENSBTAG00000038710 | *ENSBTAG00000038710* |
| ENSBTAG00000038715 | *ENSBTAG00000038715* |
| ENSBTAG00000038869 | *ZNF383* |
| ENSBTAG00000038926 | *ENSBTAG00000038926* |
| ENSBTAG00000038951 | *ENSBTAG00000038951* |
| ENSBTAG00000038970 | *ZBTB14* |
| ENSBTAG00000039023 | *ENSBTAG00000039023* |
| ENSBTAG00000039075 | *ZNF527* |
| ENSBTAG00000039111 | *ENSBTAG00000039111* |
| ENSBTAG00000039242 | *ZNF10* |
| ENSBTAG00000039287 | *ZFP69* |
| ENSBTAG00000039313 | *ZNF341* |
| ENSBTAG00000039316 | *ZNF268* |
| ENSBTAG00000039328 | *PURG* |
| ENSBTAG00000039341 | *ENSBTAG00000039341* |
| ENSBTAG00000039343 | *ZBTB34* |
| ENSBTAG00000039453 | *ENSBTAG00000039453* |
| ENSBTAG00000039493 | *ENSBTAG00000039493* |
| ENSBTAG00000039512 | *ZSCAN25* |
| ENSBTAG00000039581 | *HOXD4* |
| ENSBTAG00000039594 | *ZNF862* |
| ENSBTAG00000039599 | *HOXB4* |
| ENSBTAG00000039711 | *ZFP62* |
| ENSBTAG00000039770 | *CEBPZ* |
| ENSBTAG00000039782 | *ZNF12* |
| ENSBTAG00000039815 | *ZBTB6* |
| ENSBTAG00000039871 | *ENSBTAG00000039871* |
| ENSBTAG00000039916 | *SMAD2* |
| ENSBTAG00000039969 | *ENSBTAG00000039969* |
| ENSBTAG00000040031 | *ENSBTAG00000040031* |
| ENSBTAG00000040046 | *ENSBTAG00000040046* |
| ENSBTAG00000040061 | *ZBTB38* |
| ENSBTAG00000040063 | *ENSBTAG00000040063* |
| ENSBTAG00000040072 | *ZNF140* |
| ENSBTAG00000040082 | *HOXA10* |
| ENSBTAG00000040108 | *ENSBTAG00000040108* |
| ENSBTAG00000040169 | *ENSBTAG00000040169* |
| ENSBTAG00000040206 | *ZNF770* |
| ENSBTAG00000040209 | *ZNF112* |
| ENSBTAG00000040244 | *APOL3* |
| ENSBTAG00000040358 | *ENSBTAG00000040358* |
| ENSBTAG00000040381 | *ENSBTAG00000040381* |
| ENSBTAG00000040411 | *ENSBTAG00000040411* |
| ENSBTAG00000040442 | *ENSBTAG00000040442* |
| ENSBTAG00000040551 | *ZBTB1* |
| ENSBTAG00000040568 | *ENSBTAG00000040568* |
| ENSBTAG00000040585 | *NR6A1* |
| ENSBTAG00000040603 | *ZNF175* |
| ENSBTAG00000043949 | *PAWR* |
| ENSBTAG00000043953 | *SPIB* |
| ENSBTAG00000043974 | *ENSBTAG00000043974* |
| ENSBTAG00000043985 | *DACH1* |
| ENSBTAG00000044007 | *KLF12* |
| ENSBTAG00000044044 | *CLOCK* |
| ENSBTAG00000044074 | *MLLT10* |
| ENSBTAG00000044097 | *KLF7* |
| ENSBTAG00000044105 | *FOXO1* |
| ENSBTAG00000044123 | *HAND2* |
| ENSBTAG00000044185 | *SOX6* |
| ENSBTAG00000044192 | *MAF* |
| ENSBTAG00000045569 | *ENSBTAG00000045569* |
| ENSBTAG00000045572 | *ZNF319* |
| ENSBTAG00000045581 | *ENSBTAG00000045581* |
| ENSBTAG00000045643 | *FOXD2* |
| ENSBTAG00000045702 | *ENSBTAG00000045702* |
| ENSBTAG00000045744 | *NKRF* |
| ENSBTAG00000045791 | *ZNF623* |
| ENSBTAG00000045835 | *HOXB5* |
| ENSBTAG00000045864 | *ZNF581* |
| ENSBTAG00000045868 | *ZFP37* |
| ENSBTAG00000045877 | *TSC22D3* |
| ENSBTAG00000045912 | *ENSBTAG00000045912* |
| ENSBTAG00000045932 | *ENSBTAG00000045932* |
| ENSBTAG00000045985 | *ENSBTAG00000045985* |
| ENSBTAG00000046035 | *ENSBTAG00000046035* |
| ENSBTAG00000046101 | *ZNF470* |
| ENSBTAG00000046111 | *ZNF444* |
| ENSBTAG00000046204 | *ZNF705A* |
| ENSBTAG00000046218 | *KLF11* |
| ENSBTAG00000046258 | *ENSBTAG00000046258* |
| ENSBTAG00000046265 | *ENSBTAG00000046265* |
| ENSBTAG00000046301 | *ZNF445* |
| ENSBTAG00000046367 | *ZNF383* |
| ENSBTAG00000046409 | *EGR2* |
| ENSBTAG00000046481 | *ZNF740* |
| ENSBTAG00000046545 | *MEF2D* |
| ENSBTAG00000046556 | *ENSBTAG00000046556* |
| ENSBTAG00000046561 | *LCORL* |
| ENSBTAG00000046612 | *ZNF292* |
| ENSBTAG00000046625 | *ENSBTAG00000046625* |
| ENSBTAG00000046670 | *MLX* |
| ENSBTAG00000046684 | *FOXN3* |
| ENSBTAG00000046774 | *ZNF182* |
| ENSBTAG00000046837 | *ZNF358* |
| ENSBTAG00000046866 | *ENSBTAG00000046866* |
| ENSBTAG00000046922 | *TWIST1* |
| ENSBTAG00000046984 | *ZNF672* |
| ENSBTAG00000047120 | *ENSBTAG00000047120* |
| ENSBTAG00000047133 | *ENSBTAG00000047133* |
| ENSBTAG00000047164 | *ENSBTAG00000047164* |
| ENSBTAG00000047186 | *NME2* |
| ENSBTAG00000047219 | *ZNF793* |
| ENSBTAG00000047268 | *WT1* |
| ENSBTAG00000047405 | *ENSBTAG00000047405* |
| ENSBTAG00000047499 | *IRX2* |
| ENSBTAG00000047508 | *ZNF580* |
| ENSBTAG00000047572 | *ZNF789* |
| ENSBTAG00000047606 | *MTA1* |
| ENSBTAG00000047668 | *ENSBTAG00000047668* |
| ENSBTAG00000047680 | *IRF7* |
| ENSBTAG00000047718 | *ENSBTAG00000047718* |
| ENSBTAG00000047739 | *TSC22D1* |
| ENSBTAG00000047761 | *ENSBTAG00000047761* |
| ENSBTAG00000047855 | *MECP2* |
| ENSBTAG00000047944 | *GATA5* |
| ENSBTAG00000047954 | *ZNF75D* |
| ENSBTAG00000047955 | *ZNF774* |
| ENSBTAG00000048023 | *ENSBTAG00000048023* |
| ENSBTAG00000048286 | *E2F6* |

Table S3 Summary of transcription factors binding sites (TFBSs) significantly associated with differentially expressed (DEx) genes in muscle of Bos indicus Brahman heifers (P-value < 0.05).

| Rank | Matrix | Transcription Factor | Association Score | *P*-value | TF symbol |
| --- | --- | --- | --- | --- | --- |
| 1 | HNF1_01 | Hnf-1alpha,  Hnf-1alpha-a | 6.72 | 1.10x10^-5^ | HNF1A |
| 2 | HNF1_Q6_01 | Hnf-1alpha,  Hnf-1alpha-a | 6.33 | 2.20x10^-5^ | HNF1A |
| 3 | HNF1_Q6 | Hnf-1alpha,  Hnf-1alpha-a | 5.83 | 7.10x10^-5^ | HNF1A |
| 4 | MTATA_B | N/A | 5.43 | 1.61x10^-4^ | N/A |
| 5 | LEF1_Q2 | Lef-1, Lef-1s | 5.31 | 2.04x10^-4^ | LEF1 |
| 6 | VMAF_01 | V-maf | 4.84 | 5.72x10^-4^ | MAFK |
| 7 | SF1_Q6_01 | N/A | 4.71 | 7.13x10^-4^ | N/A |
| 8 | CEBP_Q2_01 | C/ebpalpha,  C/ebpalpha(p30) | 4.55 | 1.07x10^-3^ | PIN 1 |
| 9 | STAT5A_01 | Stat5a | 4.35 | 1.56x10^-3^ | STAT5 |
| 10 | NF1_Q6_01 | Ctf-1, Ctf-2 | 4.31 | 1.72x10^-3^ | NFIC |
| 11 | EBOX_Q6_01 | Alf1a, Bhlhb2 | 4.21 | 2.07x10^-3^ | BHLHE40 |
| 12 | VDR_Q6 | Vdr | 4.21 | 2.08x10^-3^ | VDR |
| 13 | MYOGNF1_01 | Nf-1, Nf-1/l | 4.20 | 2.11x10^-3^ | NF1 |
| 14 | CMAF_01 | C-maf | 4.14 | 2.30x10^-3^ | MAF |
| 15 | NFKB_C | N/A | 4.13 | 2.33x10^-3^ | N/A |
| 16 | NFKB_Q6 | N/A | 4.08 | 2.72x10^-3^ | N/A |
| 17 | TFIII_Q6 | Tfii-i | 4.05 | 2.84x10^-3^ | GTF21 |
| 18 | P300_01 | P300 | 3.87 | 4.14x10^-3^ | EP300 |
| 19 | CEBP_Q3 | C/ebp, C/ebpalpha | 3.77 | 4.97x10^-3^ | CEBPA |
| 20 | ETS_Q4 | Erf, Elf-1 | 3.76 | 5.26x10^-3^ | ELF1 |
| 21 | ERR1_Q2 | Err1 | 3.75 | 5.35x10^-3^ | ESRRA |
| 22 | GCNF_01 | Gcnf, Gcnf-1 | 3.74 | 5.43x10^-3^ | NR5A1 |
| 23 | E47_01 | E47 | 3.72 | 5.63x10^-3^ | TCF3 |
| 24 | NFKB_Q6_01 | Nf-kappab1,  Nf-kappab2 | 3.72 | 5.63x10^-3^ | NFKB2 |
| 25 | GZF1_01 | Gzf1 | 3.63 | 6.83x10^-3^ | ZNF336 |
| 26 | PPARA_01 | Ppar-alpha | 3.60 | 7.19x10^-3^ | PPARA |
| 27 | NFKAPPAB_01 | Rela | 3.49 | 8.97x10^-3^ | NFKB3 |
| 28 | AP2ALPHA_02 | Ap-2alphaa | 3.47 | 9.25x10^-3^ | Ap2A |
| 29 | STAT5B_01 | Stat5a, Stat5b | 3.46 | 9.42x10^-3^ | STAT5B |
| 30 | PPARG_03 | Ppar-gamma,  Ppar-gamma1 | 3.44 | 9.76x10^-3^ | PPARG |
| 31 | RORA_Q4 | N/A | 3.40 | 1.10x10^-2^ | N/A |
| 32 | HNF4_01 | Hnf-4alpha1,  Hnf-4alpha2 | 3.38 | 1.11x10^-2^ | HNF4A |
| 33 | LFA1_Q6 | N/A | 3.36 | 1.16x10^-2^ | N/A |
| 34 | PPARG_01 | Ppar-gamma,  Ppar-gamma1 | 3.32 | 1.23x10^-2^ | PPARG |
| 35 | AP2_Q3 | Ap-2alpha,  Ap-2alphaa | 3.19 | 1.59x10^-2^ | TFAP2A |
| 36 | SF1_Q6 | Sf-1 | 3.19 | 1.62x10^-2^ | NR5A1 |
| 37 | ETS2_B | C-ets-1, C-ets-2 | 3.16 | 1.72x10^-2^ | ETS2 |
| 38 | PU1_Q6 | Pu.1 | 3.16 | 1.75x10^-2^ | Spi1 |
| 39 | COUPTF_Q6 | Coup, Coup-tf1 | 3.15 | 1.77x10^-2^ | NR2F1 |
| 40 | AP2_Q6_01 | Ap-2, Ap-2alpha | 3.08 | 1.95x10^-2^ | TFAP2A |
| 41 | NFKAPPAB50_01 | N/A | 3.07 | 1.99x10^-2^ | N/A |
| 42 | FXR_IR1_Q6 | For1, For2 | 3.04 | 2.18x10^-2^ | TP53 |
| 43 | T3R_Q6 | Rar-alpha1, Rar-beta | 2.99 | 2.45x10^-2^ | RARB |
| 44 | MAZ_Q6 | Maz | 2.98 | 2.47x10^-2^ | ZNF801 |
| 45 | CEBPB_02 | C/ebpbeta(lap),  C/ebpbeta(p35) | 2.90 | 2.84x10^-2^ | CEBPB |
| 46 | ALPHACP1_01 | N/A | 2.83 | 3.21x10^-2^ | N/A |
| 47 | STAT5A_02 | Stat5a | 2.82 | 3.29x10^-2^ | STAT5 |
| 48 | STAT_01 | Stat1alpha,  Stat1beta | 2.81 | 3.36x10^-2^ | STAT1 |
| 49 | NF1_Q6 | Nf-1 | 2.78 | 3.55x10^-2^ | NF1 |
| 50 | HNF4_Q6_03 | Hnf-4, Hnf-4alpha1 | 2.73 | 3.79x10^-2^ | HNF4A |
| 51 | CP2_01 | Cp2 | 2.73 | 3.96x10^-2^ | TFCP2 |
| 52 | NFKAPPAB65_01 | Rela | 2.71 | 4.12x10^-2^ | NFKB3 |
| 53 | HIC1_02 | Hic-1 | 2.70 | 4.25x10^-2^ | ZBTB29 |
| 54 | AP2GAMMA_01 | Ap-2gamma | 2.67 | 4.46x10^-2^ | TFAP2C |
| 55 | TGIF_01 | Tgif | 2.65 | 4.57x10^-2^ | TGIF1 |
| 56 | COUP_01 | Coup-tf1,  Hnf-4alpha1 | 2.64 | 4.60x10^-2^ | HNF4A |
| 57 | PAX1_B | Pax-1 | 2.60 | 4.99x10^-2^ | PAX1 |

**Table S4** Differentially abundant proteins in comparison between pre- and post-pubertal Brahman heifers in longissimus dorsi muscle, sorted according to fold change in ascending order (P < 1.0x 10^-5^).

| Ensembl Gene ID | Uniprot ID | Gene symbol | Fold change | Adjusted *P*-value |
| --- | --- | --- | --- | --- |
| ENSBTAG00000008195 | Q29RI2 | *PHKG1* | -0.74 | 1.72x10^-7^ |
| ENSBTAG00000017121 | A0A140T897 | *ALB* | -0.65 | 1.15x10^-7^ |
| ENSBTAG00000008103 | P48644 | *ALDH1A1* | -0.64 | 1.14x10^-9^ |
| ENSBTAG00000017616 | A5PJR4 | *ADSSL1* | -0.59 | 0 |
| ENSBTAG00000031217 | Q148H2 | *MYL6B* | -0.56 | 0 |
| N/A | L8IEP3 | *M91_14325* | -0.54 | 6.41x10^-9^ |
| ENSBTAG00000051545 | Q32S29 | *H2B* | -0.41 | 1.14x10^-6^ |
| N/A | L8IL73 | *M91_02429* | -0.40 | 9.45x10^-11^ |
| N/A | L8IR03 | *M91_10531* | -0.31 | 7.04x10^-11^ |
| N/A | D4QBE8 | *HBB* | -0.29 | 1.63x10^-09^ |
| ENSBTAG00000011400 | Q3SX40 | *PDLIM7* | -0.27 | 1.53x10^-10^ |
| N/A | P28801 | *GSTP1* | -0.26 | 1.62x10^-08^ |
| N/A | M5FI55 | *SRL* | -0.25 | 4.25x10^-08^ |
| N/A | L8HQ36 | *M91_14151* | -0.23 | 1.14x10^-06^ |
| ENSBTAG00000046725 | Q148C2 | *TNNC2* | -0.23 | 1.14x10^-06^ |
| N/A | L8IV51 | *M91_15600* | -0.23 | 1.79x10^-06^ |
| ENSBTAG00000011104 | Q7YRW9 | *RTN4* | -0.21 | 3.07x10^-06^ |
| N/A | L8HRG2 | *M91_09340* | -0.19 | 3.10x10^-06^ |
| N/A | V6F832 | *CRYAB* | -0.16 | 1.72x10^-07^ |
| N/A | L8HZG4 | *M91_17580* | -0.11 | 1.11x10^-09^ |
| ENSBTAG00000001032 | F1MJ28 | *PYGM* | 0.11 | 1.89x10^-08^ |
| ENSBTAG00000016079 | P00423 | *COX4I1* | 0.13 | 1.14x10^-09^ |
| ENSBTAG00000026199 | P60712 | *ACTB* | 0.15 | 7.67x10^-07^ |
| N/A | L8IQG1 | *M91_01007* | 0.17 | 1.33x10^-07^ |
| N/A | A0A1K0FUD3 | *GLNC1* | 0.26 | 2.87x10^-13^ |
| ENSBTAG00000005431 | Q17QE2 | *LMCD1* | 0.37 | 1.53x10^-10^ |
| N/A | L8HLP9 | *M91_16830* | 0.41 | 9.33x10^-06^ |
| N/A | L8IEV9 | *M91_00825* | 0.49 | 1.04x10^-09^ |
| N/A | L8IA11 | *M91_05774* | 0.64 | 2.43x10^-08^ |

**Table S5** Enriched gene ontology (GO) terms of downregulated proteins in the comparison between pre- and post-puberty of longissimus dorsi muscle (P < 0.05). Results obtained by GOstats functional enrichment. BP: biological processes, CC: cellular component, MF: molecular function.

| Ontology | GOID | Description | Count | *P*-value | Adjusted *P*-value |
| --- | --- | --- | --- | --- | --- |
| BP | GO:0005976 | polysaccharide metabolic process | 2 | 3.5x10^-2^ | 3.0x10^-1^ |
| BP | GO:0005977 | glycogen metabolic process | 2 | 3.5x10^-2^ | 3.0x10^-1^ |
| BP | GO:0006112 | energy reserve metabolic process | 2 | 3.5x10^-2^ | 3.0x10^-1^ |
| BP | GO:0044264 | cellular polysaccharide metabolic process | 2 | 3.5x10^-2^ | 3.0x10^-1^ |
| BP | GO:0044042 | glucan metabolic process | 2 | 3.5x10^-2^ | 3.0x10^-1^ |
| BP | GO:0006073 | cellular glucan metabolic process | 2 | 3.5x10^-2^ | 3.0x10^-1^ |
| BP | GO:0009991 | response to extracellular stimulus | 1 | 5.4x10^-2^ | 3.0x10^-1^ |
| BP | GO:0031667 | response to nutrient levels | 1 | 5.4x10^-2^ | 3.0x10^-1^ |
| BP | GO:0031668 | cellular response to extracellular stimulus | 1 | 5.4x10^-2^ | 3.0x10^-1^ |
| BP | GO:0031669 | cellular response to nutrient levels | 1 | 5.4x10^-2^ | 3.0x10^-1^ |
| BP | GO:0045786 | negative regulation of cell cycle | 1 | 5.4x10^-2^ | 3.0x10^-1^ |
| BP | GO:0009267 | cellular response to starvation | 1 | 5.4x10^-2^ | 3.0x10^-1^ |
| BP | GO:0071496 | cellular response to external stimulus | 1 | 5.4x10^-2^ | 3.0x10^-1^ |
| BP | GO:0006167 | AMP biosynthetic process | 1 | 5.4x10^-2^ | 3.0x10^-1^ |
| BP | GO:0016101 | diterpenoid metabolic process | 1 | 5.4x10^-2^ | 3.0x10^-1^ |
| BP | GO:0044208 | 'de novo' AMP biosynthetic process | 1 | 5.4x10^-2^ | 3.0x10^-1^ |
| BP | GO:0032460 | negative regulation of protein oligomerization | 1 | 5.4x10^-2^ | 3.0x10^-1^ |
| BP | GO:0032459 | regulation of protein oligomerization | 1 | 5.4x10^-2^ | 3.0x10^-1^ |
| BP | GO:0034308 | primary alcohol metabolic process | 1 | 5.4x10^-2^ | 3.0x10^-1^ |
| BP | GO:0044773 | mitotic DNA damage checkpoint | 1 | 5.4x10^-2^ | 3.0x10^-1^ |
| BP | GO:0044774 | mitotic DNA integrity checkpoint | 1 | 5.4x10^-2^ | 3.0x10^-1^ |
| BP | GO:0006720 | isoprenoid metabolic process | 1 | 5.4x10^-2^ | 3.0x10^-1^ |
| BP | GO:0006721 | terpenoid metabolic process | 1 | 5.4x10^-2^ | 3.0x10^-1^ |
| BP | GO:0042594 | response to starvation | 1 | 5.4x10^-2^ | 3.0x10^-1^ |
| BP | GO:0007093 | mitotic cell cycle checkpoint | 1 | 5.4x10^-2^ | 3.0x10^-1^ |
| BP | GO:0042572 | retinol metabolic process | 1 | 5.4x10^-2^ | 3.0x10^-1^ |
| BP | GO:0034754 | cellular hormone metabolic process | 1 | 5.4x10^-2^ | 3.0x10^-1^ |
| BP | GO:0001523 | retinoid metabolic process | 1 | 5.4x10^-2^ | 3.0x10^-1^ |
| BP | GO:0001503 | ossification | 1 | 5.4x10^-2^ | 3.0x10^-1^ |
| BP | GO:0045930 | negative regulation of mitotic cell cycle | 1 | 5.4x10^-2^ | 3.0x10^-1^ |
| BP | GO:0000075 | cell cycle checkpoint | 1 | 5.4x10^-2^ | 3.0x10^-1^ |
| BP | GO:0000077 | DNA damage checkpoint | 1 | 5.4x10^-2^ | 3.0x10^-1^ |
| BP | GO:0031570 | DNA integrity checkpoint | 1 | 5.4x10^-2^ | 3.0x10^-1^ |
| BP | GO:0044262 | cellular carbohydrate metabolic process | 2 | 6.1x10^-2^ | 3.3x10^-1^ |
| MF | GO:0070279 | vitamin B6 binding | 2 | 3.9x10^-2^ | 3.7x10^-1^ |
| MF | GO:0030170 | pyridoxal phosphate binding (pyroxidal phosphate is the active form of vitamin B6) | 2 | 3.9x10^-2^ | 3.7x10^-1^ |
| MF | GO:0046872 | metal ion binding | 9 | 4.1x10^-2^ | 3.7x10^-1^ |
| MF | GO:0043169 | cation binding | 9 | 4.6x10^-2^ | 3.7x10^-1^ |
| MF | GO:0019825 | oxygen binding | 2 | 6.1x10^-2^ | 3.7x10^-1^ |
| MF | GO:0019842 | vitamin binding | 2 | 6.1x10^-2^ | 3.7x10^-1^ |
| MF | GO:0018479 | benzaldehyde dehydrogenase (NAD+) activity | 1 | 8.7x10^-2^ | 3.7x10^-1^ |
| MF | GO:0031406 | carboxylic acid binding | 1 | 8.7x10^-2^ | 3.7x10^-1^ |
| MF | GO:0004029 | aldehyde dehydrogenase (NAD) activity | 1 | 8.7x10^-2^ | 3.7x10^-1^ |
| MF | GO:0005201 | extracellular matrix structural constituent | 1 | 8.7x10^-2^ | 3.7x10^-1^ |
| MF | GO:0004019 | adenylosuccinate synthase activity | 1 | 8.7x10^-2^ | 3.7x10^-1^ |
| MF | GO:0001758 | retinal dehydrogenase activity | 1 | 8.7x10^-2^ | 3.7x10^-1^ |
| MF | GO:0033293 | monocarboxylic acid binding | 1 | 8.7x10^-2^ | 3.7x10^-1^ |
| MF | GO:0004689 | phosphorylase kinase activity | 1 | 8.7x10^-2^ | 3.7x10^-1^ |
| MF | GO:0005504 | fatty acid binding | 1 | 8.7x10^-2^ | 3.7x10^-1^ |
| MF | GO:0051087 | chaperone binding | 1 | 8.7x10^-2^ | 3.7x10^-1^ |
| MF | GO:0043177 | organic acid binding | 1 | 8.7x10^-2^ | 3.7x10^-1^ |
| MF | GO:0015643 | toxic substance binding | 1 | 8.7x10^-2^ | 3.7x10^-1^ |
| MF | GO:0003677 | DNA binding | 2 | 8.7x10^-2^ | 3.7x10^-1^ |
| MF | GO:0005516 | calmodulin binding | 2 | 1.2x10^-1^ | 3.7x10^-1^ |
| MF | GO:0043167 | ion binding | 12 | 1.3x10^-1^ | 3.7x10^-1^ |
| MF | GO:0050662 | coenzyme binding | 3 | 1.5x10^-1^ | 3.7x10^-1^ |
| MF | GO:0048037 | cofactor binding | 4 | 1.6x10^-1^ | 3.7x10^-1^ |
| MF | GO:0008483 | transaminase activity | 1 | 1.7x10^-1^ | 3.7x10^-1^ |
| MF | GO:0005212 | structural constituent of eye lens | 1 | 1.7x10^-1^ | 3.7x10^-1^ |
| MF | GO:0004069 | L-aspartate:2-oxoglutarate aminotransferase activity | 1 | 1.7x10^-1^ | 3.7x10^-1^ |
| MF | GO:0016765 | transferase activity, transferring alkyl or aryl (other than methyl) groups | 1 | 1.7x10^-1^ | 3.7x10^-1^ |
| MF | GO:0004364 | glutathione transferase activity | 1 | 1.7x10^-1^ | 3.7x10^-1^ |
| MF | GO:0004683 | calmodulin-dependent protein kinase activity | 1 | 1.7x10^-1^ | 3.7x10^-1^ |
| MF | GO:0004674 | protein serine/threonine kinase activity | 1 | 1.7x10^-1^ | 3.7x10^-1^ |
| MF | GO:0016879 | ligase activity, forming carbon-nitrogen bonds | 1 | 1.7x10^-1^ | 3.7x10^-1^ |
| MF | GO:0032550 | purine ribonucleoside binding | 2 | 1.8x10^-1^ | 3.7x10^-1^ |
| MF | GO:0032549 | ribonucleoside binding | 2 | 1.8x10^-1^ | 3.7x10^-1^ |
| MF | GO:0005525 | GTP binding | 2 | 1.8x10^-1^ | 3.7x10^-1^ |
| MF | GO:0019001 | guanyl nucleotide binding | 2 | 1.8x10^-1^ | 3.7x10^-1^ |
| MF | GO:0001882 | nucleoside binding | 2 | 1.8x10^-1^ | 3.7x10^-1^ |
| MF | GO:0001883 | purine nucleoside binding | 2 | 1.8x10^-1^ | 3.7x10^-1^ |
| MF | GO:0032561 | guanyl ribonucleotide binding | 2 | 1.8x10^-1^ | 3.7x10^-1^ |
| MF | GO:1901363 | heterocyclic compound binding | 8 | 1.9x10^-1^ | 3.7x10^-1^ |
| MF | GO:0097159 | organic cyclic compound binding | 8 | 1.9x10^-1^ | 3.7x10^-1^ |
| BP | GO:0007346 | regulation of mitotic cell cycle | 1 | 1.1x10^-1^ | 4.2x10^-1^ |
| BP | GO:0046040 | IMP metabolic process | 1 | 1.1x10^-1^ | 4.2x10^-1^ |
| BP | GO:0046033 | AMP metabolic process | 1 | 1.1x10^-1^ | 4.2x10^-1^ |
| BP | GO:0042770 | signal transduction in response to DNA damage | 1 | 1.1x10^-1^ | 4.2x10^-1^ |
| BP | GO:0043066 | negative regulation of apoptotic process | 1 | 1.1x10^-1^ | 4.2x10^-1^ |
| BP | GO:0043069 | negative regulation of programmed cell death | 1 | 1.1x10^-1^ | 4.2x10^-1^ |
| BP | GO:0051726 | regulation of cell cycle | 1 | 1.1x10^-1^ | 4.2x10^-1^ |
| BP | GO:0051657 | maintenance of organelle location | 1 | 1.1x10^-1^ | 4.2x10^-1^ |
| BP | GO:0051659 | maintenance of mitochondrion location | 1 | 1.1x10^-1^ | 4.2x10^-1^ |
| BP | GO:0051640 | organelle localization | 1 | 1.1x10^-1^ | 4.2x10^-1^ |
| BP | GO:0051646 | mitochondrion localization | 1 | 1.1x10^-1^ | 4.2x10^-1^ |
| BP | GO:0042445 | hormone metabolic process | 1 | 1.1x10^-1^ | 4.2x10^-1^ |
| MF | GO:0005488 | binding | 15 | 2.2x10^-1^ | 4.2x10^-1^ |
| MF | GO:0036094 | small molecule binding | 7 | 2.4x10^-1^ | 4.3x10^-1^ |
| MF | GO:0016769 | transferase activity, transferring nitrogenous groups | 1 | 2.4x10^-1^ | 4.3x10^-1^ |
| MF | GO:0016620 | oxidoreductase activity, acting on the aldehyde or oxo group of donors, NAD or NADP as acceptor | 1 | 2.4x10^-1^ | 4.3x10^-1^ |
| MF | GO:0016874 | ligase activity | 1 | 2.4x10^-1^ | 4.3x10^-1^ |
| MF | GO:0003676 | nucleic acid binding | 2 | 2.5x10^-1^ | 4.3x10^-1^ |
| BP | GO:0055114 | oxidation-reduction process | 3 | 1.3x10^-1^ | 4.3x10^-1^ |
| BP | GO:0033554 | cellular response to stress | 2 | 1.5x10^-1^ | 4.3x10^-1^ |
| BP | GO:0065004 | protein-DNA complex assembly | 1 | 1.5x10^-1^ | 4.3x10^-1^ |
| BP | GO:0033692 | cellular polysaccharide biosynthetic process | 1 | 1.5x10^-1^ | 4.3x10^-1^ |
| BP | GO:0009250 | glucan biosynthetic process | 1 | 1.5x10^-1^ | 4.3x10^-1^ |
| BP | GO:0005978 | glycogen biosynthetic process | 1 | 1.5x10^-1^ | 4.3x10^-1^ |
| BP | GO:0031497 | chromatin assembly | 1 | 1.5x10^-1^ | 4.3x10^-1^ |
| BP | GO:0051276 | chromosome organization | 1 | 1.5x10^-1^ | 4.3x10^-1^ |
| BP | GO:0071103 | DNA conformation change | 1 | 1.5x10^-1^ | 4.3x10^-1^ |
| BP | GO:0060548 | negative regulation of cell death | 1 | 1.5x10^-1^ | 4.3x10^-1^ |
| BP | GO:0000271 | polysaccharide biosynthetic process | 1 | 1.5x10^-1^ | 4.3x10^-1^ |
| BP | GO:0071824 | protein-DNA complex subunit organization | 1 | 1.5x10^-1^ | 4.3x10^-1^ |
| BP | GO:0051651 | maintenance of location in cell | 1 | 1.5x10^-1^ | 4.3x10^-1^ |
| BP | GO:1903047 | mitotic cell cycle process | 1 | 1.5x10^-1^ | 4.3x10^-1^ |
| BP | GO:0034728 | nucleosome organization | 1 | 1.5x10^-1^ | 4.3x10^-1^ |
| BP | GO:0006323 | DNA packaging | 1 | 1.5x10^-1^ | 4.3x10^-1^ |
| BP | GO:0006325 | chromatin organization | 1 | 1.5x10^-1^ | 4.3x10^-1^ |
| BP | GO:0006333 | chromatin assembly or disassembly | 1 | 1.5x10^-1^ | 4.3x10^-1^ |
| BP | GO:0006334 | nucleosome assembly | 1 | 1.5x10^-1^ | 4.3x10^-1^ |
| MF | GO:0016740 | transferase activity | 4 | 2.7x10^-1^ | 4.7x10^-1^ |
| BP | GO:1901615 | organic hydroxy compound metabolic process | 1 | 2.0x10^-1^ | 4.9x10^-1^ |
| BP | GO:0006974 | cellular response to DNA damage stimulus | 1 | 2.0x10^-1^ | 4.9x10^-1^ |
| BP | GO:0034637 | cellular carbohydrate biosynthetic process | 1 | 2.0x10^-1^ | 4.9x10^-1^ |
| BP | GO:0044255 | cellular lipid metabolic process | 1 | 2.0x10^-1^ | 4.9x10^-1^ |
| BP | GO:0051259 | protein complex oligomerization | 1 | 2.0x10^-1^ | 4.9x10^-1^ |
| BP | GO:0006066 | alcohol metabolic process | 1 | 2.0x10^-1^ | 4.9x10^-1^ |
| BP | GO:0022402 | cell cycle process | 1 | 2.0x10^-1^ | 4.9x10^-1^ |
| BP | GO:0006629 | lipid metabolic process | 1 | 2.0x10^-1^ | 4.9x10^-1^ |
| BP | GO:0010817 | regulation of hormone levels | 1 | 2.0x10^-1^ | 4.9x10^-1^ |
| MF | GO:0140104 | molecular carrier activity | 1 | 3.1x10^-1^ | 5.0x10^-1^ |
| MF | GO:0005344 | oxygen carrier activity | 1 | 3.1x10^-1^ | 5.0x10^-1^ |
| MF | GO:0016301 | kinase activity | 2 | 3.1x10^-1^ | 5.0x10^-1^ |
| MF | GO:0016903 | oxidoreductase activity, acting on the aldehyde or oxo group of donors | 1 | 3.7x10^-1^ | 5.4x10^-1^ |
| MF | GO:0046982 | protein heterodimerization activity | 1 | 3.7x10^-1^ | 5.4x10^-1^ |
| MF | GO:0046906 | tetrapyrrole binding | 1 | 3.7x10^-1^ | 5.4x10^-1^ |
| MF | GO:0020037 | heme binding | 1 | 3.7x10^-1^ | 5.4x10^-1^ |
| MF | GO:0004672 | protein kinase activity | 1 | 3.7x10^-1^ | 5.4x10^-1^ |
| MF | GO:0016772 | transferase activity, transferring phosphorus-containing groups | 2 | 3.8x10^-1^ | 5.5x10^-1^ |
| BP | GO:0031333 | negative regulation of protein complex assembly | 1 | 2.5x10^-1^ | 5.9x10^-1^ |
| BP | GO:0006520 | cellular amino acid metabolic process | 1 | 2.5x10^-1^ | 5.4x10^-1^ |
| BP | GO:0015980 | energy derivation by oxidation of organic compounds | 2 | 2.7x10^-1^ | 6.2x10^-1^ |
| BP | GO:0044238 | primary metabolic process | 5 | 2.9x10^-1^ | 6.2x10^-1^ |
| BP | GO:0042981 | regulation of apoptotic process | 1 | 2.9x10^-1^ | 6.2x10^-1^ |
| BP | GO:0051129 | negative regulation of cellular component organization | 1 | 2.9x10^-1^ | 6.2x10^-1^ |
| BP | GO:0043067 | regulation of programmed cell death | 1 | 2.9x10^-1^ | 6.2x10^-1^ |
| BP | GO:0016051 | carbohydrate biosynthetic process | 1 | 2.9x10^-1^ | 6.2x10^-1^ |
| BP | GO:0007049 | cell cycle | 1 | 2.9x10^-1^ | 6.2x10^-1^ |
| BP | GO:0000278 | mitotic cell cycle | 1 | 2.9x10^-1^ | 6.2x10^-1^ |
| BP | GO:0009605 | response to external stimulus | 1 | 2.9x10^-1^ | 6.2x10^-1^ |
| BP | GO:0065003 | protein-containing complex assembly | 2 | 3.0x10^-1^ | 6.2x10^-1^ |
| BP | GO:0007154 | cell communication | 2 | 3.0x10^-1^ | 6.2x10^-1^ |
| MF | GO:0008144 | drug binding | 4 | 4.5x10^-1^ | 6.3x10^-1^ |
| MF | GO:0016773 | phosphotransferase activity, alcohol group as acceptor | 1 | 4.8x10^-1^ | 6.4x10^-1^ |
| MF | GO:0000287 | magnesium ion binding | 1 | 4.8x10^-1^ | 6.4x10^-1^ |
| MF | GO:0005198 | structural molecule activity | 2 | 4.8x10^-1^ | 6.4x10^-1^ |
| BP | GO:0009058 | biosynthetic process | 3 | 3.2x10^-1^ | 6.6x10^-1^ |
| BP | GO:0043254 | regulation of protein complex assembly | 1 | 3.3x10^-1^ | 6.6x10^-1^ |
| BP | GO:0010941 | regulation of cell death | 1 | 3.2x10^-1^ | 6.6x10^-1^ |
| BP | GO:0043933 | protein-containing complex subunit organization | 2 | 3.4x10^-1^ | 6.6x10^-1^ |
| BP | GO:0048523 | negative regulation of cellular process | 2 | 3.4x10^-1^ | 6.6x10^-1^ |
| BP | GO:0048519 | negative regulation of biological process | 2 | 3.4x10^-1^ | 6.6x10^-1^ |
| MF | GO:0051287 | NAD binding | 1 | 5.2x10^-1^ | 6.9x10^-1^ |
| BP | GO:0005975 | carbohydrate metabolic process | 2 | 3.6x10^-1^ | 6.9x10^-1^ |
| BP | GO:0006950 | response to stress | 2 | 3.6x10^-1^ | 6.9x10^-1^ |
| BP | GO:0044237 | cellular metabolic process | 5 | 3.7x10^-1^ | 7.0x10^-1^ |
| BP | GO:0071704 | organic substance metabolic process | 5 | 3.7x10^-1^ | 7.0x10^-1^ |
| BP | GO:0006915 | apoptotic process | 1 | 4.0x10^-1^ | 7.2x10^-1^ |
| BP | GO:0051235 | maintenance of location | 1 | 4.0x10^-1^ | 7.2x10^-1^ |
| BP | GO:0044087 | regulation of cellular component biogenesis | 1 | 4.0x10^-1^ | 7.2x10^-1^ |
| BP | GO:0012501 | programmed cell death | 1 | 4.0x10^-1^ | 7.2x10^-1^ |
| BP | GO:0008152 | metabolic process | 5 | 4.1x10^-1^ | 7.2x10^-1^ |
| MF | GO:0008289 | lipid binding | 1 | 5.7x10^-1^ | 7.3x10^-1^ |
| MF | GO:0043168 | anion binding | 5 | 5.9x10^-1^ | 7.4x10^-1^ |
| BP | GO:0044260 | cellular macromolecule metabolic process | 2 | 4.3x10^-1^ | 7.5x10^-1^ |
| BP | GO:0008219 | cell death | 1 | 4.4x10^-1^ | 7.6x10^-1^ |
| BP | GO:0051716 | cellular response to stimulus | 2 | 4.5x10^-1^ | 7.7x10^-1^ |
| CC | GO:0043230 | extracellular organelle | 1 | 7.0x10^-1^ | 7.8x10^-1^ |
| CC | GO:0070062 | extracellular exosome | 1 | 7.0x10^-1^ | 7.8x10^-1^ |
| CC | GO:1903561 | extracellular vesicle | 1 | 7.0x10^-1^ | 7.8x10^-1^ |
| CC | GO:0005783 | endoplasmic reticulum | 2 | 1.2x10^-1^ | 7.8x10^-1^ |
| CC | GO:0005964 | phosphorylase kinase complex | 1 | 1.3x10^-1^ | 7.8x10^-1^ |
| CC | GO:1902911 | protein kinase complex | 1 | 1.3x10^-1^ | 7.8x10^-1^ |
| CC | GO:1902554 | serine/threonine protein kinase complex | 1 | 1.3x10^-1^ | 7.8x10^-1^ |
| CC | GO:0005833 | hemoglobin complex | 1 | 1.9x10^-1^ | 7.8x10^-1^ |
| CC | GO:0061695 | transferase complex, transferring phosphorus-containing groups | 1 | 1.9x10^-1^ | 7.8x10^-1^ |
| CC | GO:0000786 | nucleosome | 1 | 2.5x10^-1^ | 7.8x10^-1^ |
| CC | GO:0044815 | DNA packaging complex | 1 | 2.5x10^-1^ | 7.8x10^-1^ |
| CC | GO:0032993 | protein-DNA complex | 1 | 2.5x10^-1^ | 7.8x10^-1^ |
| CC | GO:0005581 | collagen trimer | 1 | 2.5x10^-1^ | 7.8x10^-1^ |
| CC | GO:0005794 | Golgi apparatus | 1 | 3.0x10^-1^ | 7.8x10^-1^ |
| CC | GO:0000785 | chromatin | 1 | 3.0x10^-1^ | 7.8x10^-1^ |
| CC | GO:1990234 | transferase complex | 1 | 3.0x10^-1^ | 7.8x10^-1^ |
| CC | GO:0012505 | endomembrane system | 2 | 3.1x10^-1^ | 7.8x10^-1^ |
| CC | GO:0005634 | nucleus | 3 | 3.4x10^-1^ | 7.8x10^-1^ |
| CC | GO:0031984 | organelle subcompartment | 1 | 3.5x10^-1^ | 7.8x10^-1^ |
| CC | GO:0042175 | nuclear outer membrane-endoplasmic reticulum membrane network | 1 | 3.5x10^-1^ | 7.8x10^-1^ |
| CC | GO:0005694 | chromosome | 1 | 3.5x10^-1^ | 7.8x10^-1^ |
| CC | GO:0005789 | endoplasmic reticulum membrane | 1 | 3.5x10^-1^ | 7.8x10^-1^ |
| CC | GO:0044427 | chromosomal part | 1 | 3.5x10^-1^ | 7.8x10^-1^ |
| CC | GO:0044445 | cytosolic part | 1 | 3.5x10^-1^ | 7.8x10^-1^ |
| CC | GO:0098827 | endoplasmic reticulum subcompartment | 1 | 3.5x10^-1^ | 7.8x10^-1^ |
| MF | GO:0046983 | protein dimerization activity | 1 | 6.4x10^-1^ | 8.0x10^-1^ |
| MF | GO:0005509 | calcium ion binding | 2 | 6.5x10^-1^ | 8.0x10^-1^ |
| BP | GO:0006091 | generation of precursor metabolites and energy | 2 | 4.7x10^-1^ | 8.0x10^-1^ |
| CC | GO:0032991 | protein-containing complex | 5 | 3.9x10^-1^ | 8.1x10^-1^ |
| CC | GO:0031982 | vesicle | 1 | 4.4x10^-1^ | 8.1x10^-1^ |
| CC | GO:0044421 | extracellular region part | 1 | 4.4x10^-1^ | 8.1x10^-1^ |
| CC | GO:0005615 | extracellular space | 1 | 4.4x10^-1^ | 8.1x10^-1^ |
| CC | GO:0044432 | endoplasmic reticulum part | 1 | 4.4x10^-1^ | 8.1x10^-1^ |
| BP | GO:0043170 | macromolecule metabolic process | 2 | 4.9x10^-1^ | 8.2x10^-1^ |
| BP | GO:0022607 | cellular component assembly | 2 | 4.9x10^-1^ | 8.2x10^-1^ |
| BP | GO:0009987 | cellular process | 8 | 5.0x10^-1^ | 8.2x10^-1^ |
| BP | GO:0034645 | cellular macromolecule biosynthetic process | 1 | 5.0x10^-1^ | 8.2x10^-1^ |
| BP | GO:0009059 | macromolecule biosynthetic process | 1 | 5.0x10^-1^ | 8.2x10^-1^ |
| MF | GO:1901265 | nucleoside phosphate binding | 4 | 6.9x10^-1^ | 8.3x10^-1^ |
| MF | GO:0000166 | nucleotide binding | 4 | 6.9x10^-1^ | 8.3x10^-1^ |
| BP | GO:0044085 | cellular component biogenesis | 2 | 5.1x10^-1^ | 8.3x10^-1^ |
| MF | GO:0035639 | purine ribonucleoside triphosphate binding | 3 | 7.2x10^-1^ | 8.3x10^-1^ |
| MF | GO:0042802 | identical protein binding | 1 | 7.3x10^-1^ | 8.3x10^-1^ |
| MF | GO:0032555 | purine ribonucleotide binding | 3 | 7.3x10^-1^ | 8.3x10^-1^ |
| MF | GO:0017076 | purine nucleotide binding | 3 | 7.3x10^-1^ | 8.3x10^-1^ |
| MF | GO:0032553 | ribonucleotide binding | 3 | 7.5x10^-1^ | 8.3x10^-1^ |
| MF | GO:0140096 | catalytic activity, acting on a protein | 1 | 7.6x10^-1^ | 8.3x10^-1^ |
| MF | GO:0097367 | carbohydrate derivative binding | 3 | 7.8x10^-1^ | 8.5x10^-1^ |
| CC | GO:0044464 | cell part | 10 | 4.8x10^-1^ | 8.5x10^-1^ |
| CC | GO:0005829 | cytosol | 2 | 4.9x10^-1^ | 8.5x10^-1^ |
| BP | GO:0051641 | cellular localization | 1 | 5.3x10^-1^ | 8.5x10^-1^ |
| BP | GO:0051128 | regulation of cellular component organization | 1 | 5.6x10^-1^ | 8.9x10^-1^ |
| BP | GO:0044281 | small molecule metabolic process | 3 | 5.6x10^-1^ | 8.9x10^-1^ |
| BP | GO:0034622 | cellular protein-containing complex assembly | 1 | 5.8x10^-1^ | 9.0x10^-1^ |
| BP | GO:0035556 | intracellular signal transduction | 1 | 5.8x10^-1^ | 9.0x10^-1^ |
| BP | GO:0065008 | regulation of biological quality | 2 | 5.9x10^-1^ | 9.0x10^-1^ |
| BP | GO:0044249 | cellular biosynthetic process | 2 | 5.9x10^-1^ | 9.0x10^-1^ |
| BP | GO:1901576 | organic substance biosynthetic process | 2 | 6.0x10^-1^ | 9.1x10^-1^ |
| BP | GO:0007165 | signal transduction | 1 | 6.3x10^-1^ | 9.1x10^-1^ |
| BP | GO:0050896 | response to stimulus | 2 | 6.4x10^-1^ | 9.1x10^-1^ |
| BP | GO:0065007 | biological regulation | 3 | 6.7x10^-1^ | 9.1x10^-1^ |
| BP | GO:0009168 | purine ribonucleoside monophosphate biosynthetic process | 1 | 6.8x10^-1^ | 9.1x10^-1^ |
| BP | GO:0009156 | ribonucleoside monophosphate biosynthetic process | 1 | 6.8x10^-1^ | 9.1x10^-1^ |
| BP | GO:0009124 | nucleoside monophosphate biosynthetic process | 1 | 6.8x10^-1^ | 9.1x10^-1^ |
| BP | GO:0009127 | purine nucleoside monophosphate biosynthetic process | 1 | 6.8x10^-1^ | 9.1x10^-1^ |
| BP | GO:0023052 | signaling | 1 | 6.8x10^-1^ | 9.1x10^-1^ |
| BP | GO:0030154 | cell differentiation | 1 | 6.8x10^-1^ | 9.1x10^-1^ |
| BP | GO:0009165 | nucleotide biosynthetic process | 1 | 7.0x10^-1^ | 9.1x10^-1^ |
| BP | GO:0009152 | purine ribonucleotide biosynthetic process | 1 | 7.0x10^-1^ | 9.1x10^-1^ |
| BP | GO:0009260 | ribonucleotide biosynthetic process | 1 | 7.0x10^-1^ | 9.1x10^-1^ |
| BP | GO:0006164 | purine nucleotide biosynthetic process | 1 | 7.0x10^-1^ | 9.1x10^-1^ |
| BP | GO:0048869 | cellular developmental process | 1 | 7.0x10^-1^ | 9.1x10^-1^ |
| BP | GO:0007275 | multicellular organism development | 1 | 7.0x10^-1^ | 9.1x10^-1^ |
| BP | GO:1901137 | carbohydrate derivative biosynthetic process | 1 | 7.0x10^-1^ | 9.1x10^-1^ |
| BP | GO:1901293 | nucleoside phosphate biosynthetic process | 1 | 7.0x10^-1^ | 9.1x10^-1^ |
| BP | GO:0046390 | ribose phosphate biosynthetic process | 1 | 7.0x10^-1^ | 9.1x10^-1^ |
| BP | GO:0072522 | purine-containing compound biosynthetic process | 1 | 7.0x10^-1^ | 9.1x10^-1^ |
| BP | GO:0090407 | organophosphate biosynthetic process | 1 | 7.2x10^-1^ | 9.1x10^-1^ |
| BP | GO:1901566 | organonitrogen compound biosynthetic process | 1 | 7.7x10^-1^ | 9.1x10^-1^ |
| BP | GO:0009161 | ribonucleoside monophosphate metabolic process | 1 | 7.8x10^-1^ | 9.1x10^-1^ |
| BP | GO:0009167 | purine ribonucleoside monophosphate metabolic process | 1 | 7.8x10^-1^ | 9.1x10^-1^ |
| BP | GO:0009123 | nucleoside monophosphate metabolic process | 1 | 7.8x10^-1^ | 9.1x10^-1^ |
| BP | GO:0009126 | purine nucleoside monophosphate metabolic process | 1 | 7.8x10^-1^ | 9.1x10^-1^ |
| BP | GO:0032502 | developmental process | 1 | 7.8x10^-1^ | 9.1x10^-1^ |
| BP | GO:0048856 | anatomical structure development | 1 | 7.8x10^-1^ | 9.1x10^-1^ |
| BP | GO:1901564 | organonitrogen compound metabolic process | 2 | 7.9x10^-1^ | 9.1x10^-1^ |
| BP | GO:0034654 | nucleobase-containing compound biosynthetic process | 1 | 8.0x10^-1^ | 9.1x10^-1^ |
| BP | GO:0050794 | regulation of cellular process | 2 | 8.0x10^-1^ | 9.1x10^-1^ |
| BP | GO:0009150 | purine ribonucleotide metabolic process | 1 | 8.1x10^-1^ | 9.1x10^-1^ |
| BP | GO:0009117 | nucleotide metabolic process | 1 | 8.1x10^-1^ | 9.1x10^-1^ |
| BP | GO:0009259 | ribonucleotide metabolic process | 1 | 8.1x10^-1^ | 9.1x10^-1^ |
| BP | GO:0006163 | purine nucleotide metabolic process | 1 | 8.1x10^-1^ | 9.1x10^-1^ |
| BP | GO:0006753 | nucleoside phosphate metabolic process | 1 | 8.1x10^-1^ | 9.1x10^-1^ |
| BP | GO:0019693 | ribose phosphate metabolic process | 1 | 8.1x10^-1^ | 9.1x10^-1^ |
| BP | GO:0072521 | purine-containing compound metabolic process | 1 | 8.1x10^-1^ | 9.1x10^-1^ |
| BP | GO:0019438 | aromatic compound biosynthetic process | 1 | 8.1x10^-1^ | 9.1x10^-1^ |
| BP | GO:0016043 | cellular component organization | 2 | 8.2x10^-1^ | 9.1x10^-1^ |
| BP | GO:0043436 | oxoacid metabolic process | 1 | 8.2x10^-1^ | 9.1x10^-1^ |
| BP | GO:1901362 | organic cyclic compound biosynthetic process | 1 | 8.2x10^-1^ | 9.1x10^-1^ |
| BP | GO:0019752 | carboxylic acid metabolic process | 1 | 8.2x10^-1^ | 9.1x10^-1^ |
| BP | GO:0018130 | heterocycle biosynthetic process | 1 | 8.2x10^-1^ | 9.1x10^-1^ |
| BP | GO:0006082 | organic acid metabolic process | 1 | 8.2x10^-1^ | 9.1x10^-1^ |
| BP | GO:0071840 | cellular component organization or biogenesis | 2 | 8.3x10^-1^ | 9.1x10^-1^ |
| BP | GO:0044271 | cellular nitrogen compound biosynthetic process | 1 | 8.3x10^-1^ | 9.1x10^-1^ |
| BP | GO:0006807 | nitrogen compound metabolic process | 2 | 8.4x10^-1^ | 9.1x10^-1^ |
| BP | GO:0051179 | localization | 1 | 8.4x10^-1^ | 9.1x10^-1^ |
| BP | GO:0055086 | nucleobase-containing small molecule metabolic process | 1 | 8.4x10^-1^ | 9.1x10^-1^ |
| CC | GO:0005576 | extracellular region | 1 | 5.6x10^-1^ | 9.1x10^-1^ |
| CC | GO:0043231 | intracellular membrane-bounded organelle | 5 | 5.6x10^-1^ | 9.1x10^-1^ |
| BP | GO:0019637 | organophosphate metabolic process | 1 | 8.6x10^-1^ | 9.1x10^-1^ |
| BP | GO:0050789 | regulation of biological process | 2 | 8.6x10^-1^ | 9.1x10^-1^ |
| BP | GO:1901135 | carbohydrate derivative metabolic process | 1 | 8.7x10^-1^ | 9.2x10^-1^ |
| BP | GO:0017144 | drug metabolic process | 1 | 8.7x10^-1^ | 9.2x10^-1^ |
| BP | GO:0006796 | phosphate-containing compound metabolic process | 1 | 8.9x10^-1^ | 9.3x10^-1^ |
| BP | GO:0032501 | multicellular organismal process | 1 | 9.0x10^-1^ | 9.3x10^-1^ |
| BP | GO:0006793 | phosphorus metabolic process | 1 | 9.0x10^-1^ | 9.3x10^-1^ |
| BP | GO:0006139 | nucleobase-containing compound metabolic process | 1 | 9.1x10^-1^ | 9.3x10^-1^ |
| BP | GO:0006725 | cellular aromatic compound metabolic process | 1 | 9.1x10^-1^ | 9.3x10^-1^ |
| BP | GO:0046483 | heterocycle metabolic process | 1 | 9.1x10^-1^ | 9.3x10^-1^ |
| BP | GO:1901360 | organic cyclic compound metabolic process | 1 | 9.2x10^-1^ | 9.3x10^-1^ |
| BP | GO:0006996 | organelle organization | 1 | 9.3x10^-1^ | 9.3x10^-1^ |
| BP | GO:0034641 | cellular nitrogen compound metabolic process | 1 | 9.3x10^-1^ | 9.3x10^-1^ |
| MF | GO:0005515 | protein binding | 4 | 8.8x10^-1^ | 9.3x10^-1^ |
| CC | GO:0043227 | membrane-bounded organelle | 5 | 6.0x10^-1^ | 9.4x10^-1^ |
| CC | GO:1902494 | catalytic complex | 1 | 6.2x10^-1^ | 9.5x10^-1^ |
| CC | GO:0005737 | cytoplasm | 8 | 6.7x10^-1^ | 9.6x10^-1^ |
| CC | GO:0005623 | cell | 10 | 7.0x10^-1^ | 9.6x10^-1^ |
| CC | GO:0044424 | intracellular part | 9 | 7.0x10^-1^ | 9.6x10^-1^ |
| CC | GO:0005622 | intracellular | 9 | 7.0x10^-1^ | 9.6x10^-1^ |
| CC | GO:0005886 | plasma membrane | 1 | 7.2x10^-1^ | 9.7x10^-1^ |
| CC | GO:0071944 | cell periphery | 1 | 7.5x10^-1^ | 9.7x10^-1^ |
| CC | GO:0044444 | cytoplasmic part | 6 | 7.7x10^-1^ | 9.7x10^-1^ |
| MF | GO:0005524 | ATP binding | 1 | 9.5x10^-1^ | 9.8x10^-1^ |
| MF | GO:0032559 | adenyl ribonucleotide binding | 1 | 9.6x10^-1^ | 9.8x10^-1^ |
| MF | GO:0030554 | adenyl nucleotide binding | 1 | 9.6x10^-1^ | 9.8x10^-1^ |
| MF | GO:0003824 | catalytic activity | 7 | 9.7x10^-1^ | 9.8x10^-1^ |
| CC | GO:0031224 | intrinsic component of membrane | 1 | 8.0x10^-1^ | 9.8x10^-1^ |
| CC | GO:0016021 | integral component of membrane | 1 | 8.0x10^-1^ | 9.8x10^-1^ |
| MF | GO:0016491 | oxidoreductase activity | 1 | 9.8x10^-1^ | 9.8x10^-1^ |
| CC | GO:0043226 | organelle | 6 | 8.7x10^-1^ | 1.00 |
| CC | GO:0043229 | intracellular organelle | 6 | 8.7x10^-1^ | 1.00 |
| CC | GO:0005739 | mitochondrion | 1 | 9.5x10^-1^ | 1.00 |
| CC | GO:0043228 | non-membrane-bounded organelle | 2 | 9.5x10^-1^ | 1.00 |
| CC | GO:0043232 | intracellular non-membrane-bounded organelle | 2 | 9.5x10^-1^ | 1.00 |
| CC | GO:0005856 | cytoskeleton | 1 | 9.6x10^-1^ | 1.00 |
| CC | GO:0044425 | membrane part | 1 | 9.7x10^-1^ | 1.00 |
| CC | GO:0016020 | membrane | 2 | 9.7x10^-1^ | 1.00 |
| CC | GO:0044446 | intracellular organelle part | 2 | 1.00 | 1.00 |
| CC | GO:0044422 | organelle part | 2 | 1.00 | 1.00 |
